# Three Distinct Binding Modes Govern High-Affinity Ligand Interactions with Amyloid Fibrils

**DOI:** 10.1021/acs.biochem.6c00489

**Published:** 2026-10-02

**Authors:** Timothy S. Chisholm

**Affiliations:** Yusuf Hamied Department of Chemistry, 2152 University of Cambridge, Cambridge CB2 1EW, U.K.

## Abstract

Fibrillar protein aggregates are a defining feature of neurodegenerative diseases and represent attractive biomarkers and therapeutic targets. However, the rational design of small-molecule ligands and probes targeting these aggregates is limited by a poor understanding of how ligands bind to amyloid fibrils. This work outlines an experimental approach to determine the binding mode of ligands to amyloid fibrils using previously overlooked information contained within standard binding assays. Using a mathematical framework to analyze binding behavior, three distinct high-affinity binding modes were identified: discrete, overlapping, and interacting binding. Reanalysis of published binding data indicates that these binding modes are widespread among common amyloid-binding ligand scaffolds. Two ligands were designed using principles informed by these binding modes, yielding improved binding affinities and characteristic fluorescence responses. These findings establish a practical framework for determining how small molecules bind amyloid fibrils using straightforward binding assays and for guiding the design of future fibril-binding ligands and probes.

## Graphical abstract

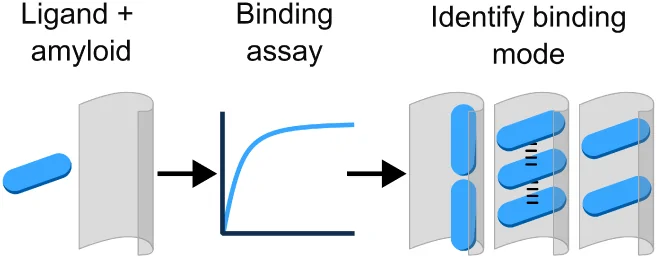

## Introduction

Many neurodegenerative diseases, such as Alzheimer’s disease (AD) and Parkinson’s disease (PD), are characterized by fibrillar protein aggregates that form in the brain.^1–3^ These aggregates, commonly referred to as amyloid fibrils, are composed primarily of amyloid-β and tau in AD and α-synuclein (αSyn) in PD. Extensive research efforts have gone toward developing high-affinity small-molecule ligands targeting amyloid fibrils for diagnostic and therapeutic applications.^4–7^ Ligands appear to bind to β-sheet-rich grooves along the fibril surface with a regular orientation and spacing, aligned either parallel or perpendicular to the length of the fibril.^8–12^ While ligands appear to bind multiple types of sites on amyloid fibrils^4,13–15^ or bind via multiple binding mechanisms,^16,17^ their precise binding mode remains obscure.

Nevertheless, recent structural studies suggest that ligand binding may occur through specific arrangements of ligands bound to the fibril surface.^18–22^ Figure 1 illustrates four such candidate binding modes for the interaction of ligands with amyloid fibrils. If a ligand binds to *discrete* sites on the fibrils, the process can be described by a simple 1:1 binding isotherm with an association constant, *K* (Figure 1a). In contrast, cryo-EM structures of fibrils with bound ligands report two distinct binding modes.^18–22^ An *interacting* binding mode, also referred to as horizontal or diagonal binding, is defined by bound ligands in close contact with each other (Figure 1b). Binding occurs with one ligand per protein monomer, and interactions between adjacent bound ligands are quantified by the cooperativity parameter ω. An *overlapping* binding mode, also referred to as vertical binding, is defined by ligands binding noncooperatively and aligned along the fibril axis spanning multiple protein monomers (Figure 1c). An interacting-overlapping binding mode is a combination of interacting and overlapping binding, where ligands bind across multiple protein monomers and also interact cooperatively (Figure 1d).

**Figure 1:**
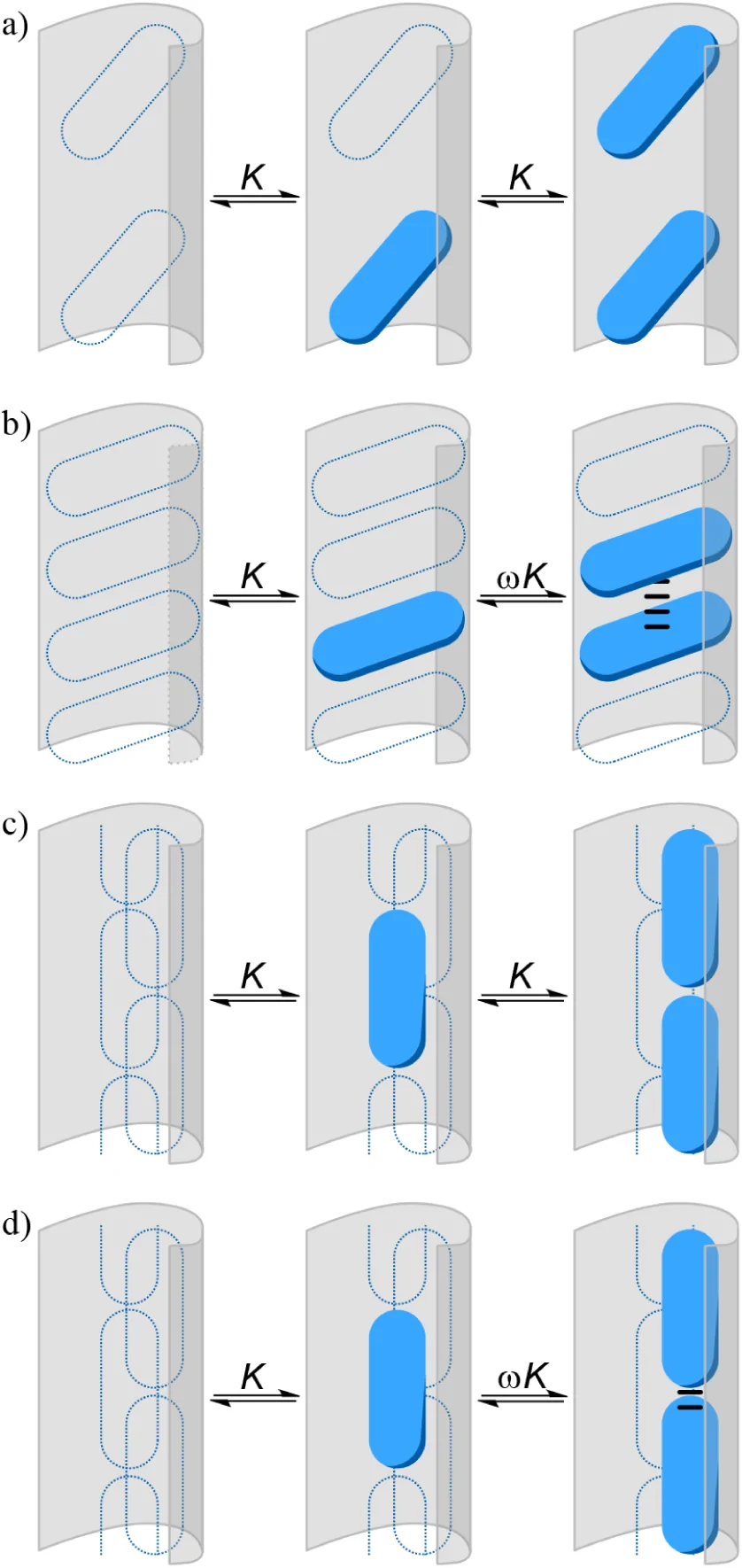
Four different binding modes for ligand (blue) interactions with an amyloid fibril (gray). (a) Discrete. (b) Interacting. (c) Overlapping. (d) Interacting-overlapping. Ligand binding sites on the fibril are indicated with dashed outlines, *K* is the association constant, and ω is the cooperativity parameter.

Cryo-EM structures are usually obtained using concentrations of ligand (50–500 μM) at least an order of magnitude more concentrated than their dissociation constants.^18–22^ Binding modes reported at high micromolar ligand concentrations therefore do not necessarily represent the structure of complexes formed at lower concentrations, where only high affinity binding sites are populated. Further evidence is therefore needed to establish which binding modes dominate under conditions more relevant to diagnostic and therapeutic applications.

One potential source of such evidence is standard ligand-binding assays. Different binding modes would be expected to alter how ligand binding evolves with ligand concentration, potentially producing characteristic features in binding isotherms. However, these data are typically analyzed using models based on the Langmuir isotherm or variations thereof. Such models, developed to analyze canonical protein–ligand interactions involving discrete binding sites, are not applicable to interacting or overlapping binding modes where ligands bind to a continuum of possibly interacting sites along an extended fibril surface.

A binding model is therefore required that can account for a continuum of adjacent sites; distinguish among discrete, interacting, overlapping, and interacting-overlapping binding modes; and incorporate multiple types of ligands or binding sites simultaneously. The model must also capture cooperative interactions between adjacent ligands and account for ligand size, where a single ligand may bind multiple protein monomers and alter the accessibility of nearby sites.

Previous work has provided a model that addresses interacting ligand binding to fibrils but that could not account for overlapping binding nor multiple types of ligands and binding sites.^23^ Here, this limitation is addressed by implementing a model using the McGhee and von Hippel procedure,^24^ enabling direct evaluation of interacting and overlapping binding modes in nanomolar binding regimes. This model enables the binding mode of a ligand to be determined from straightforward binding assays, yielding effective structural information. As a proof of principle, ligands were designed on the basis of this information to yield improved binding properties.

## Results and Discussion

### Derivation of a Binding Model

A binding model was first derived for the interacting overlapping binding of a single type of ligand to a single type of site on a 1D polymer, representing a binding groove on an amyloid fibril, while accounting for both ligand size and cooperativity. This approach is readily generalizable to a model describing the binding of multiple types of ligands to multiple types of sites on a 1D polymer (see SI).

As a reference, for a discrete binding mode, the concentration of binding sites is conserved according to the equation

1 $${[s]}_{t}=\left[s\right]+\left[L\cdot s\right]$$

where [*s*]_*t*_ is the total concentration of binding sites, [*s*] is the concentration of free binding sites, and [*L* · *s*] is the concentration of bound ligand. This equation is not valid for overlapping binding on a 1D polymer, where the concentration of available binding sites also depends on the size and spatial distribution of bound ligands (Figure 2a). This challenge can be addressed following a procedure described by McGhee and von Hippel^24^ and later expanded by Villaluenga et al.,^25^ to count the number of possible binding sites on a 1D polymer by describing ligand binding using conditional probabilities.

**Figure 2:**
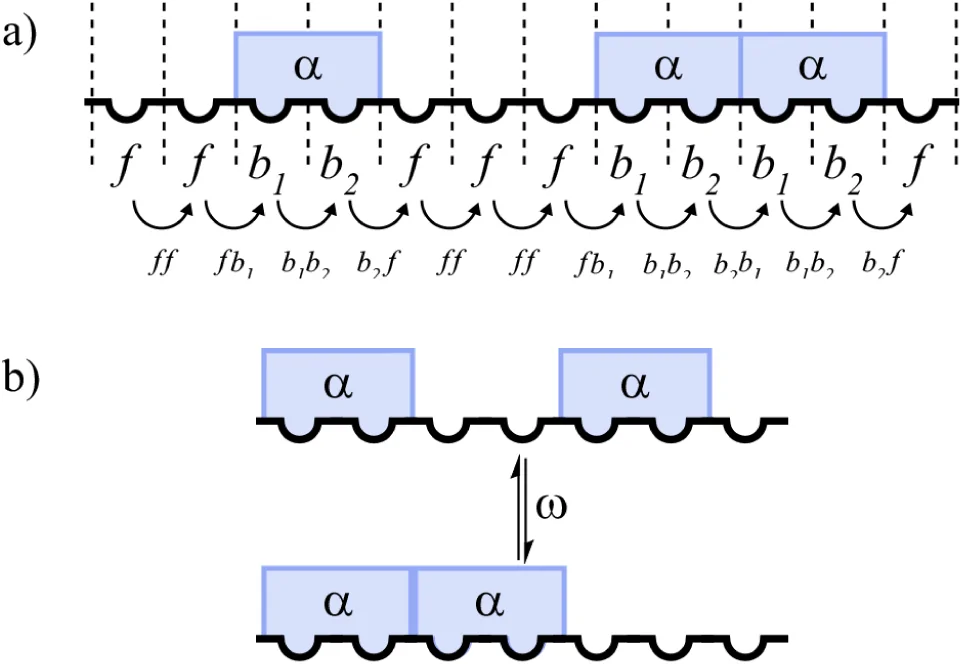
(a) Schematic illustrating the binding of a ligand (α) to lattice residues, the type of lattice residue (*f* or *b_k_*), and the conditional probabilities. (b) Definition of the cooperativity parameter ω used in this model.

In this procedure, a linear 1D polymer is modeled as a lattice composed of *N* individual residues (Figure 2a). In the context of amyloid fibrils, each fibril is typically composed of two protofilaments, each with potentially multiple binding grooves. This model represents a single type of binding groove, where each lattice residue is equivalent to a protein monomer. A ligand, α, covers *m* lattice residues when bound and is described as consisting of *m* segments labeled 1, 2, ..., *m* from left to right. There are, therefore, 1 + *m* possible types of residues in the linear polymer. Free residues are denoted *f*, and residues bound to a ligand are denoted by *b_k_* for segment *k* of the ligand.

Conditional probabilities are then defined as sequences of two types of lattice residues. There are four relevant conditional probabilities: (*ff*) is the probability that, given a free residue, there is an adjacent free residue to the right; (*fb*_1_) is the probability that, given a free residue, there is an adjacent residue bound to the first section of a ligand to the right; (*b_m_ f*) is the probability that, given a residue bound to the final section of a ligand, that there is an adjacent free residue to the right; (*b_m_ b_1_*) is the probability that, given a residue bound to the final section of a ligand, that there is an adjacent residue bound to the first section of a ligand to the right.

Using these conditional probabilities, equations can be derived to model an interacting-overlapping binding mode.

First, only two types of residues can lie to the immediate right of a free residue, yielding

2 $$\left(ff\right)+\left(fb_{1}\right)=1$$

If the final segment of a bound ligand is selected, only a free site or a second bound ligand can lie to the immediate right, giving

3 $$\left(b_{m}f\right)+\left(b_{m}b_{1}\right)=1$$

The coverage of a ligand is then defined as the fraction of lattice residues bound to a ligand,

4 $$\theta =\frac{nm}{N}$$

where *n* ligands are bound. If a residue is selected at random, the next residue will be free with a probability (1 – θ), giving

5 $$\left(1-\theta \right)\left(ff\right)+\frac{\theta }{m}\left(b_{m}f\right)=1-\theta$$

Cooperative binding affects these conditional probabilities and can be accounted for by introducing a cooperativity parameter, ω, describing the equilibrium between binding configurations where two ligands are adjacent or nonadjacent (Figure 2b). While these conditional probabilities describe local adjacency, the number of available binding sites depends on stretches of free residues. A gap is defined as a sequence of *g* free lattice residues, where *g* = 0 corresponds to adjacent ligands, and a ligand can only bind if *g* ≥ *m*. Note that while conditional probabilities are defined per lattice residue, binding site availability is most naturally expressed per gap, following the statistical treatment introduced by McGhee and von Hippel.^24^ This approach allows for the average number of free ligand binding sites per gap, *s̅*, to be computed (see SI for the full derivation),

6 $$\mathrm{s̅}={[\omega +\frac{\left(ff\right)}{1-\left(ff\right)}]}^{2}\left(b_{m}f\right)\left(fb_{1}\right){\left(ff\right)}^{m-1}$$

The total number of free ligand-binding sites can then be written as *s̅* · *g*. As the total number of bound ligands is equal to the number of gaps plus one, the concentration of free binding sites [*s*] is *s̅*[*L* · *s*] for *g* ≫ 1, leading to the mass action equation,

7 1=Ks̅[L·s]

These equations form the basis of a binding model that models all four binding modes shown in Figure 1. This model describes overlapping binding when ω = 1, interacting binding when *m* = 1, and a discrete binding mode when ω = 1 and *m* = 1. The discrete binding mode (Figure 1a) will be used as a reference point in this work and is equivalent to a noncooperative interacting binding mode (ω = 1) or an overlapping binding mode where the ligand size is 1 (*m* = 1). In practice, an interacting-overlapping model is overparameterized for fitting experimental data and is physically unlikely as axially aligned ligands will have a relatively small contact area for cooperativity. Analysis hereon therefore focuses on discrete (*m* = 1, ω = 1), interacting (*m* = 1), or overlapping (ω = 1) binding modes only.

To enable this approach for modeling binding to a groove on an amyloid fibril, this model assumes a static and infinitely long fibril, with uniform lattice sites along the binding groove length, and ligands that are either symmetric or exclusively bind in a single orientation.

Electron microscopy suggests that fibrils in the brain may be several hundred nanometers in length,^26^ with a β-strand separation within a protofilament of approximately 4.8 Å.^27,28^ Each binding groove on a protofilament, therefore, represents several hundred lattice units. The structures of individual monomers likely differ at the protofilament ends, but these represent a minority of the overall lattice sites present. However, on highly fragmented or sonicated fibrils that are shorter in length, the structural differences at protofilament ends may have a larger effect on the bulk binding properties measured.

The uniformity of protein fibrils, and the frequency of structural defects or post-translational modifications along their length, is not well characterized. The success of cryo-EM helical reconstructions implies that the cross-β lattice of fibrils is regular over distances greatly exceeding the footprint of a bound ligand, suggesting fibrils are sufficiently uniform for the purposes of this model. However, cryo-EM structures are biased toward more ordered fibrils, and there may exist fibrils with structural and chemical variation along their length, which are poorly represented by a uniform lattice model.

This model also assumes that binding mode does not change along the fibril length, nor as a function of lattice occupancy. Ligand binding is known to perturb local fibril structure,^22^ but any perturbation affecting adjacent sites is absorbed into ω. Perturbations extending beyond adjacent sites are not captured. It is also assumed that cooperative interactions do not extend beyond adjacent sites.

### Modeling Saturation Binding

The developed mathematical model was applied to compute isotherms for a single type of ligand binding to a single type of site via a discrete binding mode (*m* = 1, ω = 1), an overlapping binding mode (*m* = 4, ω = 1), and an interacting binding mode (*m* = 1, ω = 4).

The simulated binding isotherms for all three binding modes display a similar shape (Figure 3a). However, the Scatchard plots (Figure 3b), Hill plots (Figure 3c), and plots of the local Hill coefficient (*n*_*H*_) against coverage (θ) (Figure 3d) reveal diagnostic differences. Interacting binding produces a concave-down Scatchard plot, a slope greater than one in the Hill plot, and a concave-down Hill coefficient plot. Overlapping binding leads to a concave-up Scatchard plot, a slope below one in the Hill plot, and a monotonically decreasing Hill coefficient plot. The shape of the Scatchard and Hill plots generated by the overlapping binding mode mimics characteristics of negative cooperativity as a ligand binding to one site blocks access to other sites, meaning that increasing binding occupancy reduces the apparent binding affinity. The binding mode-specific features of these plots are summarized in Table 1. In practice, local Hill coefficient plots generated from experimental data are typically obscured by experimental noise. Subsequent analyses will, therefore, be based on Scatchard and Hill plots.

**Figure 3:**
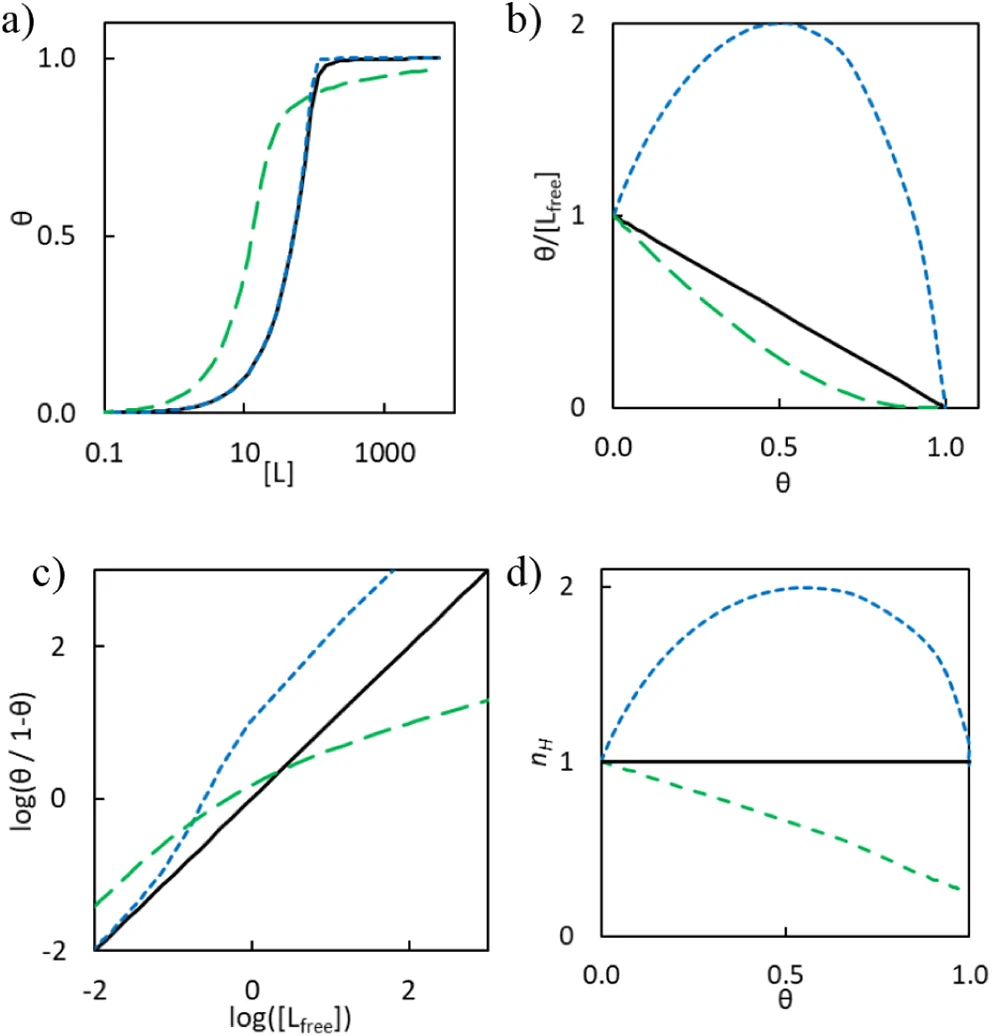
Representative (a) binding isotherms, (b) Scatchard plots, (c) Hill plots, and (d) local Hill coefficient (*n_H_*) plot for discrete binding (black line), interacting binding (*m* = 1, ω = 4, blue short-dashed line), and overlapping binding (*m* = 4, ω = 1, green long-dashed line), calculated with *K* = 1 and a lattice residue concentration of 100.

**Table 1:** Diagnostic Features of Different Binding Modes

Scatchard Plot Features	Hill Plot Features	Local Hill Coefficient Features	Binding Mode
Concave down	Slope > 1	Maxima	Interacting
Concave up	Slope < 1	Decreasing	Overlapping
Linear, slope = 1	Slope = 1	nH = 1	Discrete

An interacting-overlapping binding mode yields Scatchard and Hill plots with features resembling interacting binding if ω dominates and overlapping binding if *m* dominates (Figure S26, Table S1). At comparable magnitudes of *m* and ω, a concave-down Scatchard plot resembling interacting binding combined with a Hill plot with a decreasing slope at high occupancy resembling overlapping binding will be observed. Additionally, this is the only binding mode that will produce a Hill coefficient plot that exceeds one at low coverage and falls below one at high coverage.

In addition to this qualitative analysis of the shape of Scatchard and Hill plots, a quantitative treatment to determine binding mode is implemented using the open source software tool, Musketeer.^29^ Musketeer can be used to evaluate binding isotherms with an arbitrary number of free and bound species while simultaneously optimizing unknown parameters, such as the optical brightness of spectroscopically active species or the concentration of different equilibrium components. The mathematical framework developed here is incorporated into Musketeer to fit ligand binding to an amyloid fibril using the described binding modes (see SI). To test for overlapping binding, experimental data are fitted using Musketeer across a range of integer values of *m*. The RMSE of the generated fits is then used to find an optimal value for *m*, with a value of *m* > 1 suggesting that overlapping binding occurs. To test for interacting binding, the cooperativity parameter ω is directly optimized during the fitting procedure. As incorporating ω introduces an additional fitted parameter and increases model complexity, competing models are compared using the Akaike Information Criterion with small-sample correction (AICc).^30,31^ A value of ω > 1 suggests interacting binding occurs. By determining ω and *m*, the binding mode of a ligand can, therefore, be determined. For the interacting and overlapping binding modes, different optical properties for ligands bound adjacently and nonadjacently can also be considered.

Not all diagnostic features of interacting and overlapping binding modes are unique. Notably, binding to two discrete binding sites will also generate a concave up Scatchard plot (Figure S27). More generally, Scatchard plot features do not report the number of binding sites present, and experimental noise can obscure Hill plot features indicative of multiple sites (Table S1). In these instances, quantitative analysis and comparisons using AICc are more reliable. However, binding sites with low abundance or poor binding affinity will remain challenging to identify regardless of the analysis used.

### Reanalysis of Fluorescence Binding Assays

This approach was used to reanalyze previously reported data for the binding of the fluorescent ligand Thioflavin T (**ThT**) to patient-derived Parkinson’s disease αSyn fibrils (PD fibrils, Figure 4), and recombinant αSyn fibrils aggregated at pH 6.5 (f65 fibrils, Figure 5).^32^ Generating Scatchard and Hill plots requires knowledge of the lattice binding site concentration, which is typically undetermined in binding assays using amyloid fibrils. However, the shapes of the Scatchard and Hill plots were qualitatively similar across different lattice residue concentrations (Figure S16).

**Figure 4:**
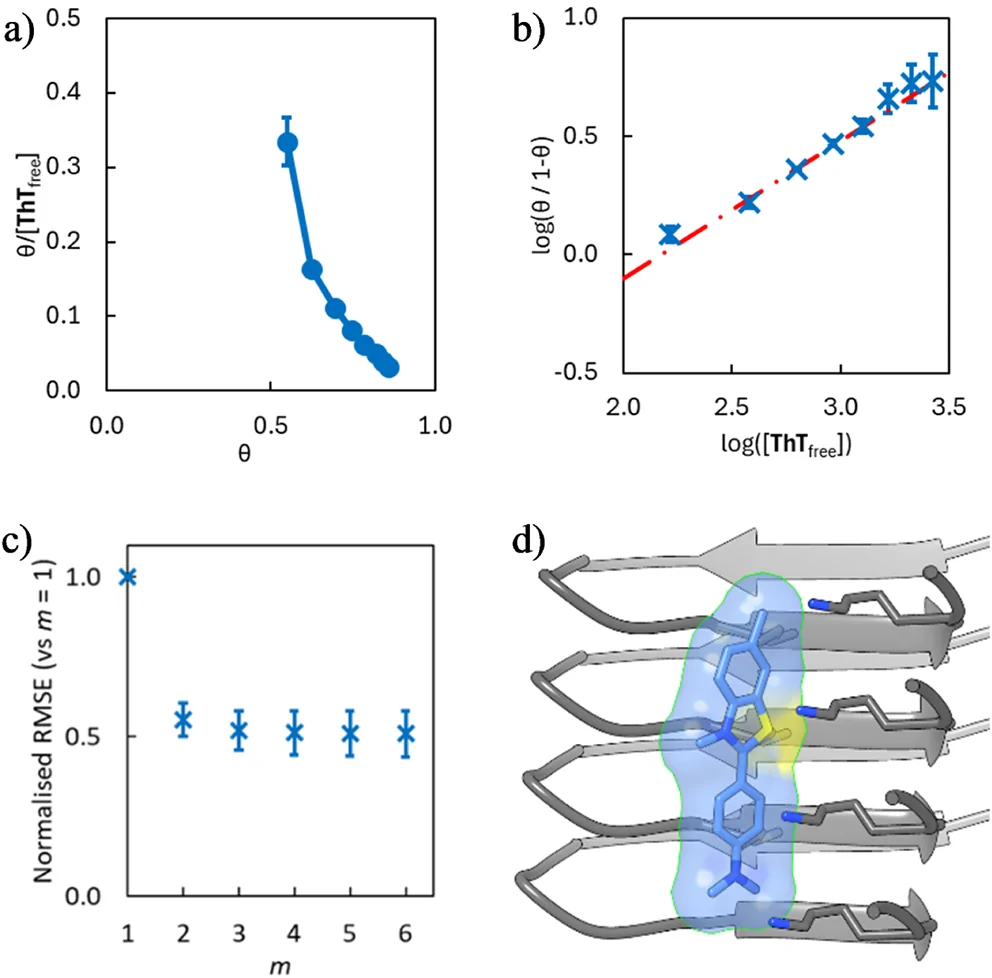
Data from a fluorescence binding assay of **ThT** (λ_ex_ = 440 nm, λ_em_ = 483 nm) into PD fibrils (500 nM). The (a) Scatchard plot and (b) Hill plot, showing a linear line of best fit (red dashed-dotted line; slope = 0.58 ± 0.03). (c) Normalized RMSE (relative to *m* = 1) for fits to overlapping binding modes with different values of *m*. (d) Cryo-EM structure of **ThT** bound to αSyn fibrils (PDB: 8X7B).^19^ Data points are the average of at least three experimental measurements, with 95% confidence intervals shown.

**Figure 5:**
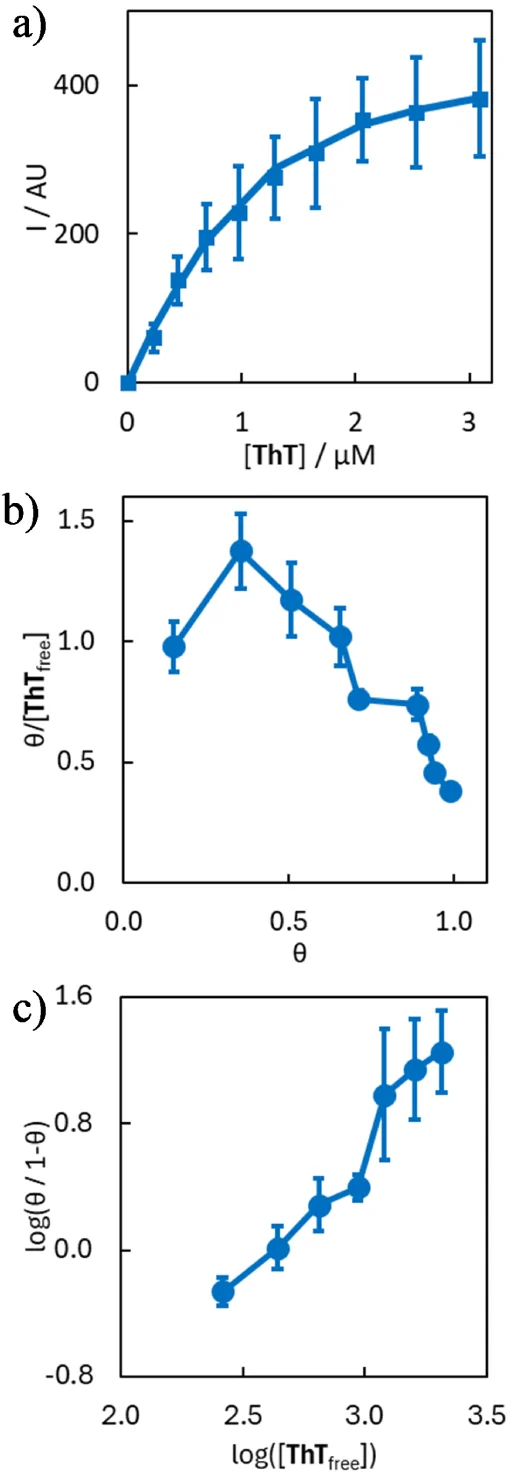
(a) Data from a fluorescence binding assay of **ThT** (λ_ex_ = 440 nm, λ_em_ = 483 nm) into f65 fibrils (500 nM) with a line of best fit to a discrete binding model (*m* = 1, ω = 1), with the corresponding (b) Scatchard plot and (c) Hill plot. Data points are the average of at least three experimental measurements, with 95% confidence intervals shown.

For **ThT** binding to PD fibrils, a concave-up Scatchard plot and a Hill plot with a slope less than one were generated, indicative of an overlapping binding mode (Figure 4a,b). Screening different values of *m* during fitting of the raw data showed that *m* = 1 gave an unacceptable fit (RMSE = 0.17), while larger values of *m* gave statistically indistinguishable fits (RMSE ≤ 0.09 for *m* ≥ 2; Figure 4c). This result is consistent with an overlapping binding mode (*m* > 1). Notably, *m* = 4 is consistent with reported cryo-EM structures showing **ThT** bound in an overlapping mode spanning four protein monomers (Figure 4d; PDB ID: 8X7B).^19^ A concave-up Scatchard plot can also arise from binding to two discrete sites. Fitting this two-site model gave a poorer fit (ΔAICc = 10) and did not converge to two physically meaningful sites, with the second dissociation constant outside the titrated concentration range with an uncertainty equal to its magnitude (*K*_*d,1*_ = 200 ± 50 nM; *K*_*d,2*_ = 20 ± 20 M).

For **ThT** binding to f65 fibrils, both a concave-down Scatchard plot and a Hill plot with a slope greater than one were generated, consistent with an interacting binding mode (Figure 5a–c). Fitting the raw data with an interacting binding mode yielded a cooperativity parameter ω of 1.73, but did not significantly improve the fit compared to a discrete binding model (ΔAICc = 0.9).^30,31^ Together, these data demonstrate the plausibility of both overlapping and interacting binding modes and show that the same ligand can engage in different binding modes to different fibril structures.

### Radioligand Self-Displacement Assays

Data indicative of different binding modes can also be obtained by comparing radioligand saturation and self-displacement assays fitted with a simple Langmuir model.

For an overlapping binding mode, ligand binding progressively reduces the number of accessible binding sites, as each bound ligand blocks binding to adjacent lattice residues. During a radioligand saturation assay, binding, therefore, becomes progressively less favorable with increasing occupancy. In contrast, during a radioligand self-displacement assay, a bound radiolabeled ligand is displaced by a structurally identical unlabeled ligand, resulting in little net change to lattice occupancy or site blocking. These occupancy-dependent effects contribute to the apparent dissociation constant measured in a saturation assay (*K*_*d*_) but are largely absent when measuring the dissociation constant measured by a self-displacement assay (*K*_*i*_). Self-displacement assays are, therefore, expected to yield stronger apparent binding than saturation assays for an overlapping binding mode (*K*_*i*_ < *K*_*d*_).

For an interacting binding mode, ligand binding alters the favorability of adjacent binding events through cooperativity. During a saturation assay, increasing occupancy increases the fraction of available binding sites adjacent to bound ligands, meaning that cooperativity contributes to the measured apparent dissociation constant. During a self-displacement assay, exchange of a radiolabeled ligand for an identical unlabeled ligand minimally affects the total cooperative interactions present. An interacting binding mode is, therefore, expected to result in stronger apparent binding when measured by a saturation assay than by a self-displacement assay (*K*_*i*_ > *K*_*d*_). For both binding modes, the magnitude of the difference will depend on the radioligand concentration used in the self-displacement assay and the corresponding lattice occupancy.

There are 23 examples from the literature where the dissociation constant of a radioligand has been measured using both saturation and self-displacement assays and screened with the same fibril target in the same publication (Figure 6a).^33–55^ Previous work has found that the median variation in error of reported binding constants in single studies was 0.35 log units.^4^ A difference of 1.0 log units between *K*_*i*_ and *K*_*d*_ was chosen to constitute a significant difference, and four examples meeting this threshold were found (Figure 6b).^33–35^ **TZDM** and **THK523** had a higher dissociation constant measured from self-displacement assays, indicative of cooperative binding, while **PiB** and **MODAG-001** had a higher dissociation constant measured from saturation assays, indicative of either overlapping binding or binding to two discrete sites with different binding affinities. While **TZDM** and **PiB** are structurally similar, they appear to bind via different modes to different fibril morphologies. These different binding modes are, therefore, generalizable beyond specific ligand scaffolds and targets.

**Figure 6:**
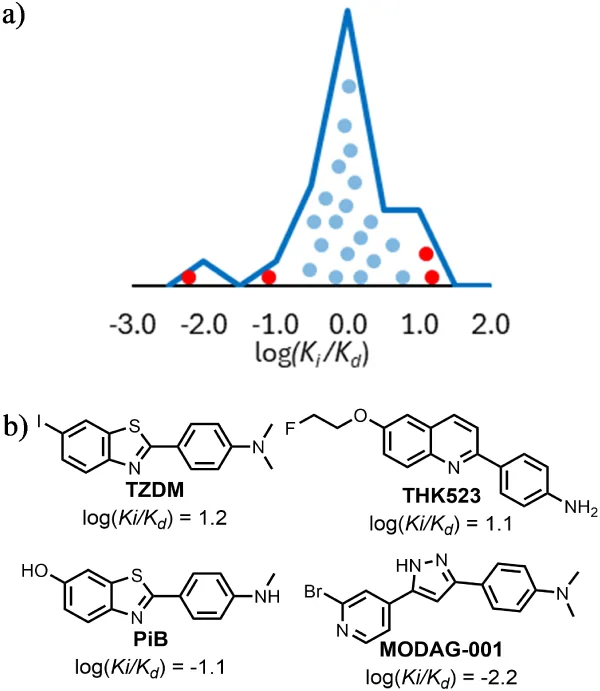
(a) Distribution of log­(*K_i_*/*K_d_*) values for radioligands with dissociation constants measured from saturation and self-displacement assays in the same publication. (b) Ligands with a difference in dissociation constants of at least an order of magnitude, shown in red in panel (a).

### Binding Mode-Informed Ligand Design

The presence of overlapping and interacting binding modes suggests that new design principles can be applied for developing high-affinity ligands. The binding mode is a property of the ligand-fibril complex, rather than the ligand alone, and reflects the geometry and interactions of both components. The ligand size *m* is based on the orientation of the ligand within the binding groove. As protein monomers within a protofilament are separated by a β-strand spacing of approximately 4.8 Å,^27,28^ narrow grooves that enforce alignment along the fibril axis require ligands to span multiple monomers, producing overlapping binding. Wider grooves can accommodate ligands bound at an angle to the fibril axis or within a single monomer layer, allowing discrete or interacting binding.

Cooperativity ω is then determined by whether adjacent ligands interact favorably. Interacting binding is favored by planar ligands capable of stacking and disfavored by bulky substituents or interligand charge repulsion. These effects are modulated by hydrophobic, polar, and charged residues lining the binding groove, the degree of desolvation of both ligand and fibril upon binding, and, for charged ligands, the ionic strength of the buffer. The same ligand can therefore adopt different binding modes to different polymorphs,^19^ and a given groove may be bound via different modes by different ligands.

The binding mode present also determines the maximum number of ligands that can bind to a target, which may be an important consideration in applications such as imaging. To test these principles, ligands were prepared based on **ThT** as a known binding motif (Figure 7). Ligand **1** incorporates two **ThT** headgroups joined to increase the length of the ligand. A short linker was chosen to minimize the ability of each headgroup to bind in a different groove and to minimize any added entropic penalty upon binding.^56^ Ligand **2** incorporates a naphthalene diimide (NDI) moiety that self-associates in aqueous media to form supramolecular polymers driven by π–π interactions, thereby favoring interacting binding to allow these stacking interactions to occur.^57–59^

**Figure 7:**
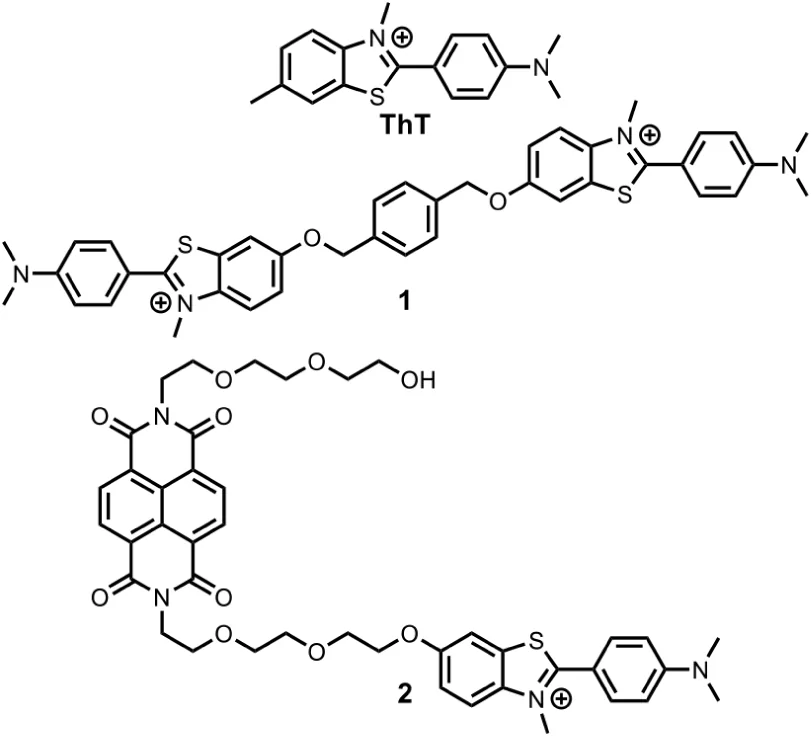
Structures of the **ThT** derivatives used.

Binding assays were performed using **ThT**, **1**, and **2** against αSyn fibrils (Figure 7). Two fibril morphologies were used: αSyn-PBS fibrils were prepared in 1× PBS buffer, and αSyn-Tris fibrils were prepared in Tris buffer. Different fibril morphologies, while composed of the same protein, have different chemical and biological properties, including distinct ligand-binding sites.^13^ The fluorescence emission of the ThT headgroups (λ_ex_ = 440 nm) was monitored, alongside the fluorescence of the NDI moiety of **2** (λ_ex_ = 332 nm). Models with the same number of fitted parameters were evaluated by comparing the RMSE of the fit, and models with different numbers of fitted parameters were evaluated and compared using AICc.

Binding of **ThT** to αSyn fibrils produced an enhanced emission at 483 nm upon excitation at 440 nm (Figure 8a). Data fitting supported a discrete binding mode to αSyn-PBS fibrils and an overlapping binding mode to αSyn-Tris fibrils (Figure 8b). These results are supported by Scatchard and Hill analyses (Figures 8c–f and S17–S18). Binding of **ThT** to αSyn-PBS fibrils yielded a linear Scatchard plot and a Hill plot with a slope of 0.9 ± 0.1, consistent with discrete binding (Figure 8g). Binding of **ThT** to αSyn-Tris fibrils yielded a concave-up Scatchard plot and a Hill plot with a slope of 0.8 ± 0.1, consistent with overlapping binding (Figure 8h). Fitting the data to an overlapping binding mode with *m* = 4, consistent with the length of **ThT** in reported cryo-EM structures (Figure 4d), yielded relatively weak binding (*K*_*d*_ = 8,000 ± 1,000 nM) compared to the discrete binding of **ThT** to αSyn-PBS fibrils (*m* = 1, *K*_*d*_ = 1,600 ± 500 nM).

**Figure 8:**
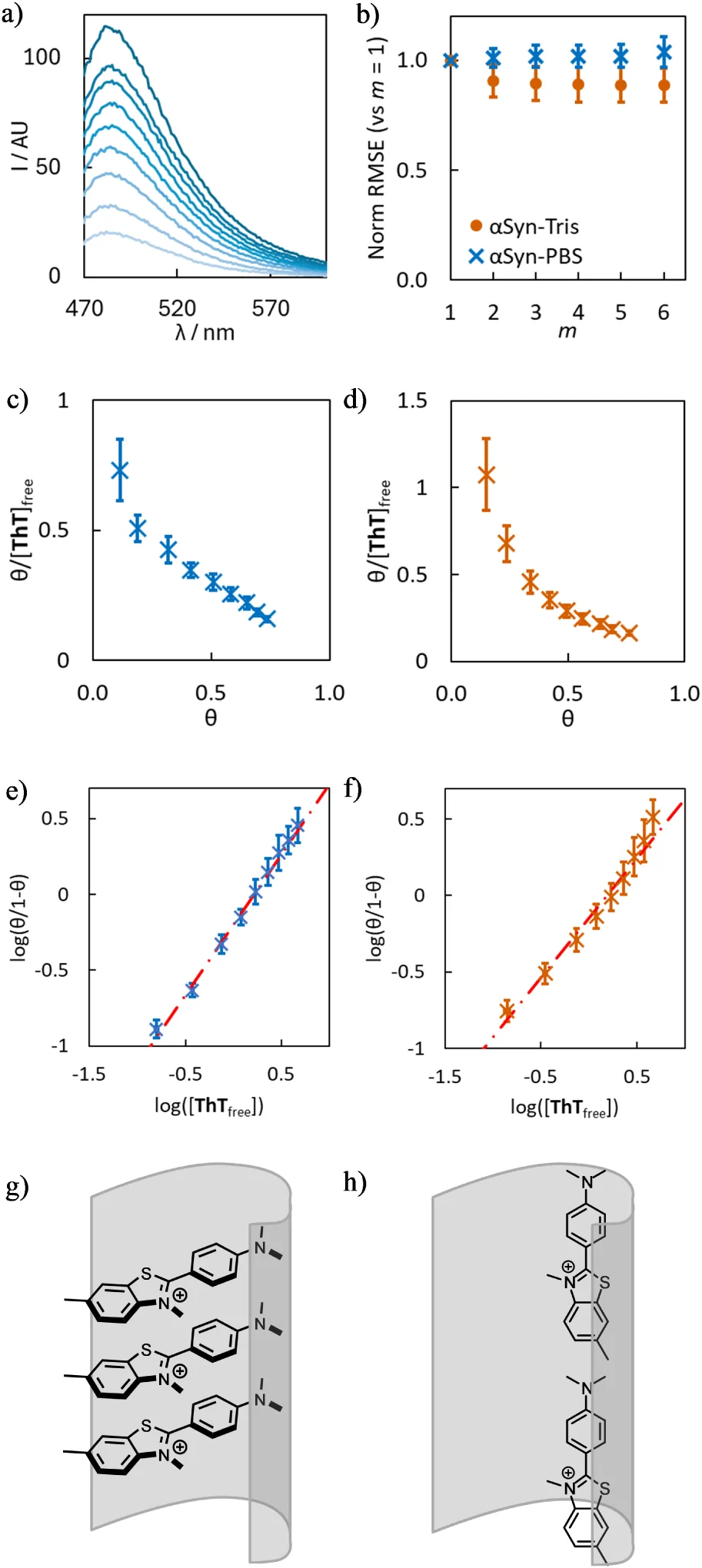
Data from fluorescence binding assays of **ThT** (λ_ex_ = 440 nm) with αSyn-Tris and αSyn-PBS fibrils (500 nM) in 1× PBS (pH 7.4). (a) Representative emission spectra showing increasing **ThT** concentrations (0–5 μM) added to αSyn-Tris fibrils. (b) The RMSE of binding assay data fitted to an overlapping binding mode with different values of *m*, normalized to the RMSE of a fit to a discrete binding mode (*m* = 1) (blue crosses: αSyn-PBS; orange circles: αSyn-Tris). Scatchard plots for **ThT** binding to (c) αSyn-PBS or (d) αSyn-Tris fibrils, and Hill plots with a line of best fit (red dashed-dotted line) for **ThT** binding to (e) αSyn-PBS (fitted slope = 0.9 ± 0.1) or (f) αSyn-Tris fibrils (fitted slope = 0.8 ± 0.1). Schematics illustrating the proposed binding modes of **ThT** to (g) αSyn-PBS and (h) αSyn-Tris fibrils. Data points are the average of at least three experimental measurements, with 95% confidence intervals shown.

Upon titration of the dimer **1** into either αSyn fibril preparation, the fluorescent emission at 483 nm from the ThT headgroup decreased concomitantly with the emergence of a new emission band at 582 nm (Figure 9a–d). This red-shifted emission is indicative of ThT excimers.^60,61^ Excimer formation was modeled by fitting different emission spectra for adjacently bound ligands and nonadjacently bound ligands.

**Figure 9:**
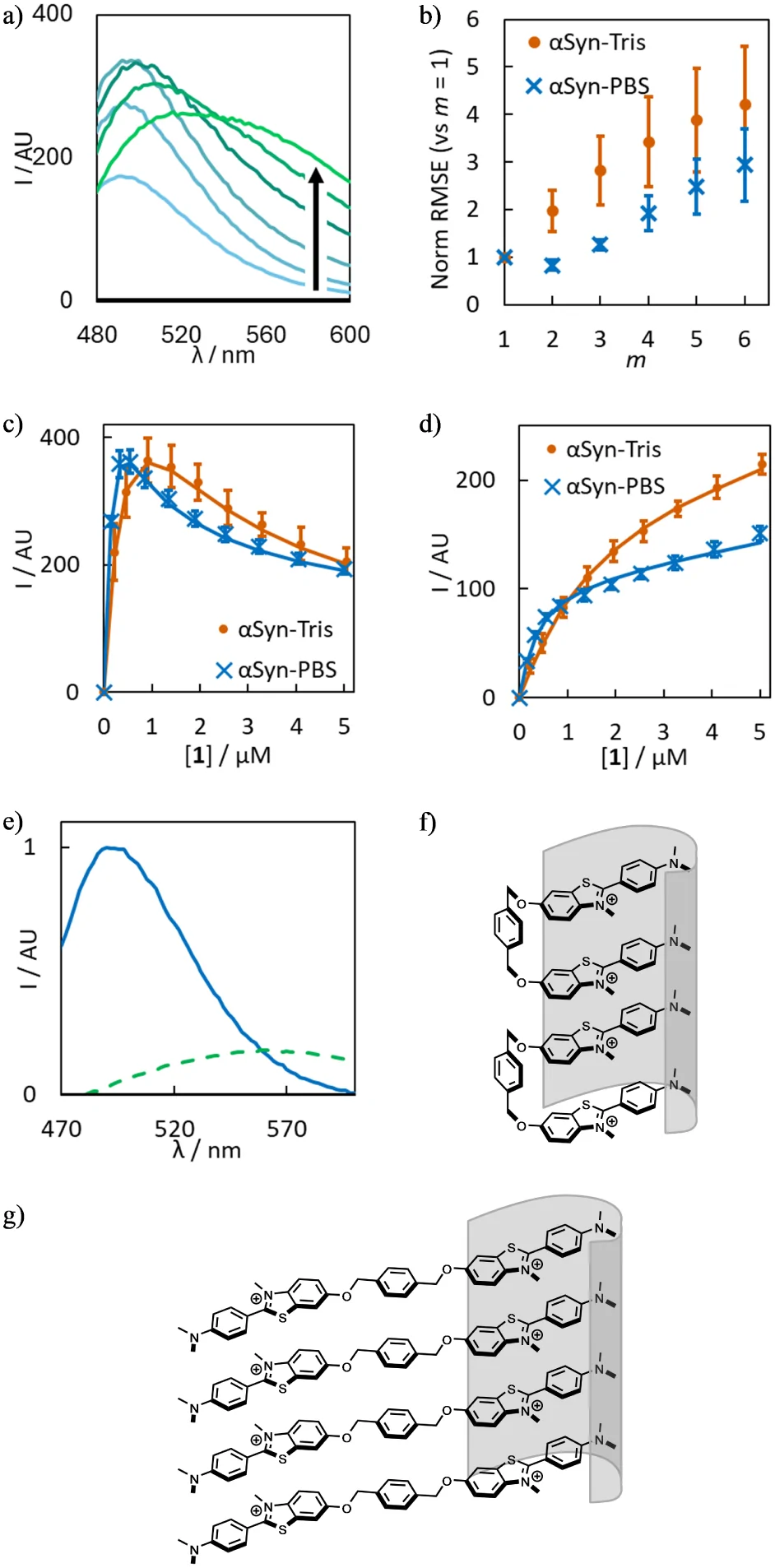
Data from fluorescence binding assays of **1** (λ_ex_ = 440 nm) into αSyn-Tris and αSyn-PBS fibrils (500 nM) in 1× PBS (pH 7.4). (a) Representative emission spectra for increasing concentrations of **1** added to αSyn-Tris fibrils. (b) The RMSE of binding assay data fitted to an overlapping binding mode with different values of *m*, normalized to the RMSE of a fit to a discrete binding mode (*m* = 1) (blue crosses: αSyn-PBS; orange circles: αSyn-Tris). Fluorescence titration data monitored at (c) λ_em_ = 483 nm and (d) λ_em_ = 582 nm. Lines are the best fit to an overlapping binding mode for binding to αSyn-PBS fibrils (*m* = 2, ω = 1) and a discrete binding mode for binding to αSyn-Tris fibrils (*m* = 1, ω = 1). (e) Representative fitted emission spectra for nonadjacently bound **1** (solid blue line) and adjacently bound **1** (dashed green line) to αSyn-PBS fibrils. Schematics illustrating the proposed binding modes of **1** to (f) αSyn-PBS and (g) αSyn-Tris fibrils. Data points are the average of at least three experimental measurements, with 95% confidence intervals shown.

Fits with the lowest RMSE were obtained for ligand **1** binding to αSyn-PBS in an overlapping binding mode (*m* = 2, ω = 1, *K*_*d*_ = 200 ± 40 nM) and to αSyn-Tris in a discrete binding mode (*m* = 1, ω = 1, *K*_*d*_ = 840 ± 80 nM) (Figure 9b). Fitted emission spectra for nonadjacently bound **1** show the expected emission at 483 nm, whereas those for adjacently bound **1** show emission at 582 nm (Figure 9e; see SI). A value of *m* = 2 for binding to αSyn-PBS fibrils may reflect binding of **1** in a folded conformation where the ThT headgroups are in close proximity (Figure 9f). In contrast, only a single ThT headgroup may bind to αSyn-Tris fibrils to allow for *m* = 1 (Figure 9g). These results suggest that a long, narrow ligand structure alone is insufficient to enforce an overlapping binding mode.

For the binding of **2**, NDI excitation at 332 nm produced a new emission band at 440 nm upon binding both fibril morphologies (Figure 10a). This emission is red-shifted and stronger than the emission observed in the absence of fibrils (see SI), in agreement with literature observations for the formation of π-stacked NDI supramolecular polymers in aqueous media.^62^ A discrete binding model (*m* = 1, ω = 1), allowing for differences in emission of adjacently and nonadjacently bound **2**, gave the best fit for both fibril targets (Figure 10b,c). Binding of **2** to αSyn-PBS (*m* = 1, ω = 1, *K*_*d*_ = 1,800 ± 100 nM) was not enhanced compared to **ThT** (*m* = 1, ω = 1, *K*_*d*_ = 1,600 ± 500 nM), but binding of **2** to αSyn-Tris (*m* = 1, ω = 1, *K*_*d*_ = 600 ± 60 nM) was significantly stronger than that of **ThT** (*m* = 4, ω = 1, *K*_*d*_ = 8,000 ± 1,000 nM). Fitted emission spectra for excitation at 332 nm show a weak emission for nonadjacently bound **2**, and an enhanced emission for adjacently bound **2** that is red-shifted compared to the free ligand (Figure 10d,e).

**Figure 10:**
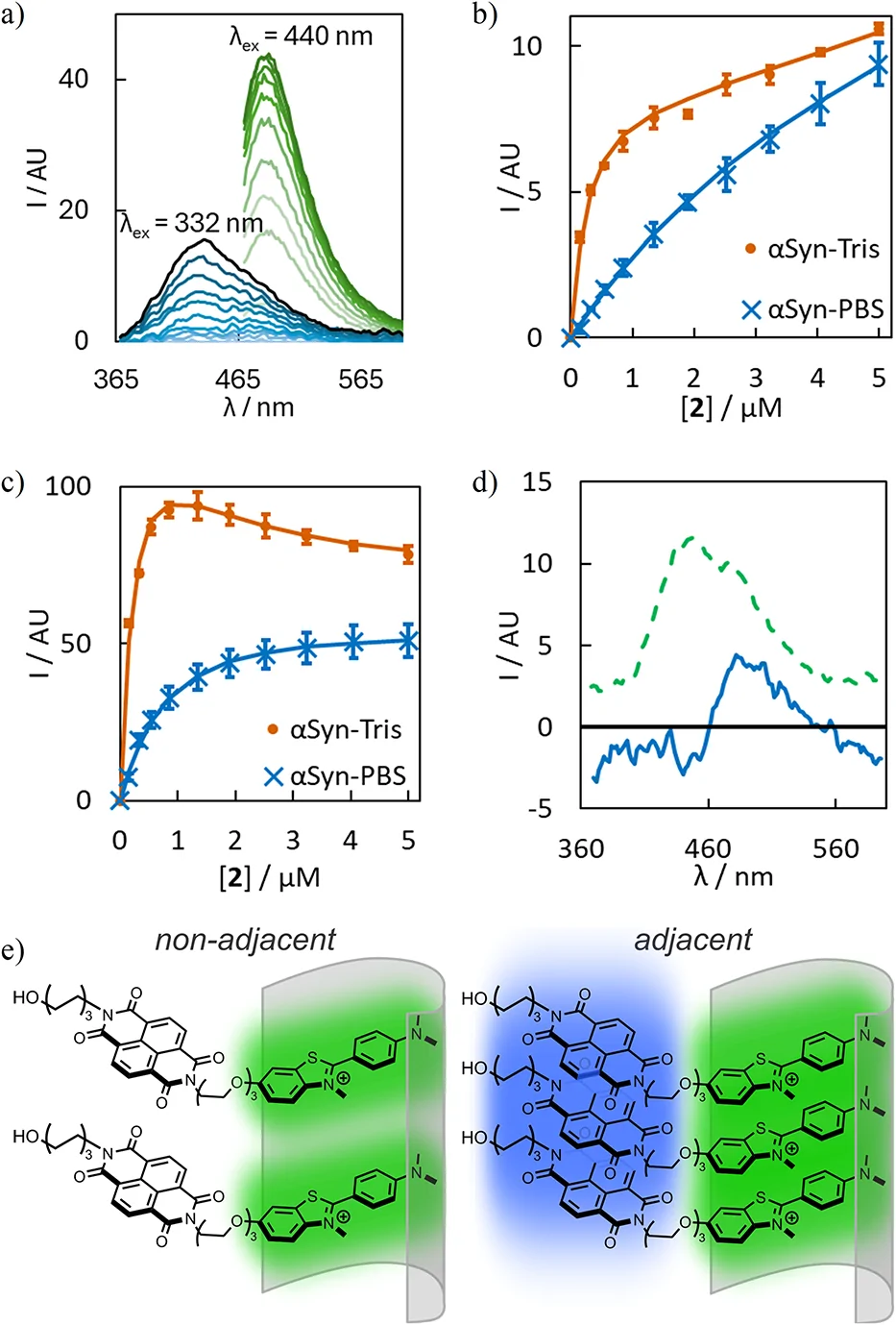
Data from fluorescence binding assays of **2** with αSyn-Tris and αSyn-PBS fibrils (500 nM) in 1x PBS (pH 7.4). (a) Representative emission spectra (λ_ex_ = 332 nm, λ_ex_ = 440 nm) for increasing concentrations of **2** added to αSyn-PBS fibrils. Fluorescence titration data monitored at (b) λ_ex_ = 332 nm; λ_em_ = 440 nm and (c) λ_ex_ = 440; λ_em_ = 483 nm. Lines are the best fit to a discrete binding isotherm (*m* = 1, ω = 1) accounting for different emission spectra for adjacently and nonadjacently bound **2**. (d) Representative fitted emission spectra (λ_ex_ = 332 nm) for nonadjacently bound **2** (solid blue line) and adjacently bound **2** (dashed green line) from a fluorescence binding assay with αSyn-PBS fibrils. (e) Schematics illustrating the proposed nonadjacent and adjacent binding modes of **2**. Data points are averages of at least three experimental measurements, with 95% confidence intervals shown.

These results support the existence of distinct interacting and overlapping binding modes and provide a proof of principle that binding-mode-informed ligand design can produce enhanced ligand binding properties and unique fluorescence readouts.

## Conclusion

This work outlines an experimental approach to determine the binding mode of ligands to amyloid fibrils using standard binding assays combined with qualitative and quantitative analysis. These data support the presence of overlapping and interacting binding modes, and reanalysis of published data indicates these binding modes are likely widespread. Knowledge of these binding modes can then inform the design of ligands, such as **1** and **2**, which exhibited improved binding properties to αSyn fibrils. Altogether, this work enables information on the binding mode of amyloid ligands to be obtained from straightforward binding assays and provides a more accurate framework to both design future ligands and understand their interactions with amyloid fibrils.

## Methods

### Small Molecule Synthesis

**6,6′-((1,4-Phenylenebis­(methylene))­bis­(oxy))­bis­(2-(4-(dimethylamino)­phenyl)-3-methylbenzo­[** *d* **]­thiazol-3-ium) diiodide** (**1**)

To 2-(4-(dimethylamino)­phenyl)­benzo­[*d*]­thiazol-6-ol (216 mg, 0.80 mmol, 2.0 equiv) in DMF (5 mL) was added sodium hydride (60% in mineral oil, 29 mg, 1.20 mmol, 3.0 equiv) in one portion at room temperature. The resultant yellow suspension was stirred for 1 h, producing a green solution. 1,4-*Bis*(bromomethyl)­benzene (102 mg, 0.39 mmol, 0.98 equiv) was then added, and the mixture was heated to 90 °C for 19 h. After cooling to room temperature, ice water (15 mL) was added with vigorous stirring. The resultant precipitate was collected by filtration and washed with ice water (10 mL) and then ice-cold ethanol (10 mL) to afford a poorly soluble intermediate (77.9 mg).

A mixture of the intermediate (19.3 mg, 30 μmol) and iodomethane (0.5 mL) in nitrobenzene (2.0 mL) was heated to 110 °C for 4 h under microwave irradiation. The reaction was cooled to room temperature and triturated using diethyl ether on ice. The precipitate was collected by filtration and washed with ice-cold diethyl ether (50 mL) to afford a yellow solid (28.0 mg, 30 μmol, 100%). ^**1**^ **H NMR (400 MHz, DMSO-** *d*_***6***_ **) δ (ppm)**: 8.17 (d, *J* = 9.2 Hz, 2H), 8.07 (s, 2H), 7.79 (d, *J* = 8.7 Hz, 4H), 7.60–7.48 (m, 6H), 6.97 (d, *J* = 8.7 Hz, 4H), 5.29 (s, 4H), 4.21 (s, 6H), 3.12 (s, 12H). ^**13**^ **C NMR (176 MHz, DMSO-** *d*_***6***_ **) δ (ppm)**: 171.5, 157.7, 153.5, 137.1, 136.1, 132.1, 129.7, 128.2, 118.6, 117.9, 112.0, 111.0, 107.8, 69.9, 39.7, 38.2. **HRMS (ESI+)**: 336.5048 *m*/*z*: Calculated for C_40_H_40_N_4_O_2_S_2_^2+^ = 336.5308 [M]^2+^.

**2-(2-(2-(2-((2-(4-(Dimethylamino)­phenyl)­benzo­[** *d* **]­thiazol-6-yl)­oxy)­ethoxy)­ethoxy)­ethoxy)­ethyl)-7-(2-(2-(2-hydroxyethoxy)­ethoxy)­ethoxy)­ethyl)­benzo­[lmn]­[3,8]­phenanthroline-1,3,6,8­(2** *H*,**7** *H* **)-tetraone** (**S4**)

To 2-(4-(dimethylamino)­phenyl)­benzo­[d]­thiazol-6-ol (23.0 mg, 85 μmol, 1.1 equiv) and sodium hydride (60% in mineral oil, 8.2 mg, 4.4 equiv) in DMF (0.8 mL) was added S3 (52.9 mg, 77 μmol, 1.0 equiv) in DMF (0.7 mL) on ice. The reaction was warmed to room temperature and stirred for 30 min, producing a green solution that was heated to 80 °C for 17 h. The residue was diluted in CH_2_Cl_2_ (50 mL), and the organic layer was washed with 1 M KOH (3 × 20 mL) and brine (20 mL) and dried over Na_2_SO_4_. The crude residue was then purified by flash silica column chromatography (CH_2_Cl_2_/MeOH) to afford **S4** as a yellow solid (25.5 mg, 33 μmol, 43%). ^**1**^ **H NMR (400 MHz, CDCl**_**3**_ **) δ (ppm)**: 8.64 (s, 4H), 7.78 (d, *J* = 8.9 Hz, 2H), 7.67 (d, *J* = 8.9 Hz, 1H), 7.13 (d, *J* = 2.5 Hz, 1H), 6.86 (dd, *J* = 8.9, 2.6 Hz, 1H), 6.69 (dt, *J* = 8.9, 2.2 Hz, 2H), 4.40 (t, *J* = 6.0 Hz, 2H), 4.34 (t, *J* = 5.8 Hz, 2H), 4.03 (t, *J* = 4.8 Hz, 2H), 3.88–3.78 (m, 6H), 3.74–3.70 (m, 4H), 3.67–3.65 (m, 2H), 3.63–3.58 (m, 4H), 3.57–3.48 (m, 2H), 3.04 (s, 6H). ^**13**^ **C NMR (101 MHz, CDCl**_**3**_ **) δ (ppm)**: 162.9, 156.2, 152.0, 149.0, 135.6, 130.9, 128.5, 122.6, 121.5, 115.4, 111.8, 105.5, 77.4, 72.5, 70.9, 70.6, 70.5, 70.2, 69.9, 68.2, 61.8, 40.3, 29.8. **HRMS (ESI+)**: 783.2698 *m*/*z*: Calculated for C_41_H_43_N_4_O_10_S^+^ = 783.2695 [M + H]^+^. 805.2520 *m*/*z*: Calculated for C_41_H_42_N_4_O_10_SNa^+^ = 805.2519.

**2-(4-(Dimethylamino)­phenyl)-6-(2-(2-(2-(7-(2-(2-(2-hydroxyethoxy)­ethoxy)­ethyl)-1,3,6,8-tetraoxo-3,6,7,8-tetrahydrobenzo­[lmn]­[3,8]­phenanthrolin-2­(1** *H* **)-yl)­ethoxy)­ethoxy)­ethoxy)-3-methylbenzo­[** *d* **]­thiazol-3-ium Iodide** (**2**)

A mixture of **S4** (23.5 mg, 30 μmol) and iodomethane (0.5 mL) in nitrobenzene (2.0 mL) was heated to 110 °C for 5 h under microwave irradiation. The reaction was cooled to room temperature and triturated using diethyl ether on ice. The precipitate was collected by filtration and washed with ice-cold diethyl ether (50 mL) to afford **2** as a brown wax (26.6 mg, 28 μmol, 93%). ^**1**^ **H NMR (400 MHz, DMSO) δ (ppm):** 8.62–8.57 (m, 4H), 8.05 (d, *J* = 9.4 Hz, 1H), 7.80–7.76 (m, 3H), 7.33 (dd, *J* = 9.3, 2.2 Hz, 1H), 6.96 (d, *J* = 8.9 Hz, 2H), 4.51 (t, *J* = 5.3 Hz, 2H), 4.25 (t, *J* = 6.2 Hz, 2H), 4.20–4.18 (m, 5H), 4.10–4.04 (m, 2H), 3.76–3.72 (m, 4H), 3.65 (t, *J* = 6.7 Hz, 2H), 3.62–3.60 (m, 3H), 3.57–3.50 (m, 2H), 3.49–3.45 (m, 2H), 3.37 (t, *J* = 5.0 Hz, 2H), 3.11 (s, 6H). ^**13**^ **C NMR (176 MHz, DMSO) δ (ppm):** 171.2, 162.5, 162.5, 157.8, 153.5, 136.8, 132.0, 130.5, 130.4, 129.6, 126.1, 126.0, 126.0, 118.2, 117.7, 112.0, 112.0, 110.9, 107.1, 72.3, 69.8, 69.6, 69.6, 68.6, 68.1, 66.7, 60.1. **HRMS (ESI+):** 797.2993 *m*/*z*: Calculated for C_42_H_45_N_4_O_10_S^+^ = 797.2851 [M + H]^+^.

### Generation of Synthetic Binding Isotherms

Synthetic binding isotherms were generated using the supplementary iPython notebook “*Model—Main Fitter.ipynb*”. For isotherms with a single ligand and a single type of binding site, values of *K* = 1 and host concentration = 100 were fixed. Additional parameters were fixed for discrete (ω = 1, *m* = 1), overlapping (ω = 1, *m* = 4), and interacting (ω = 4, *m* = 1) binding modes.

### Radioligand Assay Data Set

Data were extracted from a previously reported database of amyloid-binding ligands.^5^ Inclusion criteria were that radioligands must have a dissociation constant reported by both a saturation assay and a self-displacement assay, where identical structures were tested with the exception of radioisotopes, and that these data must be reported in a single publication. Assays must be against the same fibril target using a single set of buffer conditions at the same temperature. The data for the ligands used are in the supplementary “Radioligand data.csv” file provided.

### Preparation of αSyn Fibrils

A solution of monomeric αSyn (recombinant, *E. coli*) was added to an Amicon Ultra-15 centrifugal filter (15 kDa MWCO) and centrifuged (15 min, 4000*g*). The retained monomeric αSyn was washed with 5 mL of the aggregation buffer; either 1× PBS (pH 7.4) or Tris·HCl (50 mM, pH 7.5) and NaCl (100 mM), and centrifuged (15 min, 4000*g*). This wash step was repeated four times in total. The retained filtrate was diluted to 1 mL using the aggregation buffer and incubated at 37 °C for 72 h with gentle agitation using a magnetic stir bar in an Eppendorf LoBind microcentrifuge tube (2.0 mL). The resultant fibrils were then pelletized in a centrifuge (15 min, 4000*g*), the supernatant was removed, and the fibrils were gently resuspended in the desired buffer. The fibril preparation was then stored at −80 °C for 2 years. Characterization data of these fibril samples have been previously reported.^63^

### Fluorescence Binding Assays

Stock solutions of ligand in DMSO (1.0 mM) and αSyn fibrils in 1× PBS (pH 7.4, 50 μM) were prepared. Titrations were performed through the addition of a mixture of ligand and αSyn fibrils diluted in 1× PBS (20 μM ligand, 500 nM αSyn fibril, pH 7.4) into αSyn fibrils diluted in 1× PBS (500 nM αSyn fibril, pH 7.4, 37 °C) in a fluorescence quartz cuvette. Fluorescence spectra were obtained, and titrations were repeated with at least three independent replicates.

### Data Fitting

Binding assay data were fit using the open source software *Musketeer* implementing the custom speciation file *speciation_1Dbinding.py*.^26^ The measured fluorescence spectra, ligand concentration, and free ligand fluorescence spectra were input. The binding constant, host (αSyn fibril) concentration, and fluorescence spectra of the bound species were left as fitted variables. The cooperativity parameter, ω, was fixed to 1 for discrete or overlapping binding modes, or left as a fitted variable for an interacting binding mode. The ligand size, *m*, was fixed to 1 for discrete or interacting binding modes, or systematically varied for an overlapping binding mode.

## Supplementary Material





## Notes

Experimental procedures, compound characterization, analytical and spectroscopic data, binding model derivation, synthetic binding data, and instructions to generate synthetic binding data (PDF)
Data set of radioligands (CSV); binding model code, modified Musketeer code, and associated readme file (IPYNB, PY, TXT) (ZIP)
