# Supplementary material for: Three Distinct Binding Modes Govern High-Affinity Ligand Interactions with Amyloid Fibrils
Source: Biochemistry. 2026 Oct 2;65(20):3299–309. doi: 10.1021/acs.biochem.6c00489 (PMC13689812; doi:10.1021/acs.biochem.6c00489)
Supplement: Supplementary file 1 [file bi6c00489_si_001.pdf]

Supporting Information

Three Distinct Binding Modes Govern High-Affinity Ligand Interactions with Amyloid Fibrils

Timothy S. Chisholm*,[a]

[a] Yusuf Hamied Department of Chemistry, University of Cambridge, Lensfield Road, Cambridge CB2 1EW, UK. Email: tsc42@ch.cam.ac.uk.

Contents

Materials and Instrumentation S3

Small Molecule Synthesis S4

UV-Visible Characterisation S15

Fluorescence Characterisation S16

Additional Scatchard Plots and Hill Plots S18

Binding Model Derivation S20

- Cooperativity parameter S20
- Gap probability S20
- Computing the number of free binding sites S20
- Multi-ligand, multi-site binding model equations S22
- Final system of equations S24

Synthetic Binding Data S26

- Additional one-site synthetic binding data S26
- Overlapping new-site synthetic binding data S28
- Two-site synthetic binding data S29
- Synthetic binding data generation S31

Code for Isotherm Generation and Fitting. S34

Comparing Fits S35

- Comparing fitted dissociation constants for different values of *m* S36

Symbols Used in Equations S37

References S38

Materials and Instrumentation

All solvents and chemicals were obtained from commercial sources and used without further purification unless otherwise stated. Protein LoBind (Eppendorf) microtubes were used for preparing and storing all solutions containing protein. All buffers were prepared with Milli-Q water and filtered through 0.22 µm filters.

Reactions were monitored by TLC or LCMS. TLC analyses were performed on Merck TLC Silica gel 60 F_{254} glass plates (0.2 mm). LCMS analyses of samples were performed using a Waters Acquity H-class UPLC coupled with a single quadrupole Waters SQD2. An Acquity UPLC CSH C18 Column, 130Å, 1.7 µm, 2.1 mm x 50 mm was used as the UPLC column.

Purification of compounds by silica column chromatography were performed using an automated system (Combiflash® Rf+) with prepackaged silica cartridges (25 µm or 50 µm PuriFlash® columns). ^{1}H and ^{13}C NMR spectra were recorded using a Bruker 600 MHz Avance 600 BBI spectrometer, a 500 MHZ Acance III Smart Probe spectrometer, or a 400 MHz Avance III HD Smart Probe spectrometer at 298.0 ± 0.1 K. Residual solvent peaks were used as an internal standard for calibration. All chemical shifts are quoted in ppm on the δ scale and the coupling constants are expressed in Hz. Signal splitting patterns are described as a singlet (s), broad singlet (br s), doublet (d), triplet (t), quartet (q), or multiplet (m).

UV-vis spectra were collected on an Agilent Cary 60 UV-vis spectrophotometer controlled by Cary WinUV software. Fluorescence spectroscopic data were recorded using an Agilent Cary Eclipse Fluorescence Spectrophotometer controlled by Cary WinUV software, and equipped with a Cary Eclipse Automated Polarizer for anisotropy measurements.

Small Molecule Synthesis

2-(4-(dimethylamino)phenyl)benzo[d]thiazol-6-ol (**S1**)

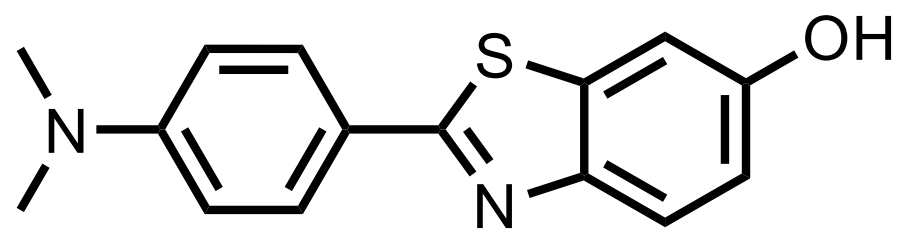

S1 was prepared as previously reported.[1]

6,6'-((1,4-phenylenebis(methylene))bis(oxy))bis(2-(4-(dimethylamino)phenyl)-3-methylbenzo[*d*]thiazol-3-ium) diiodide (1)

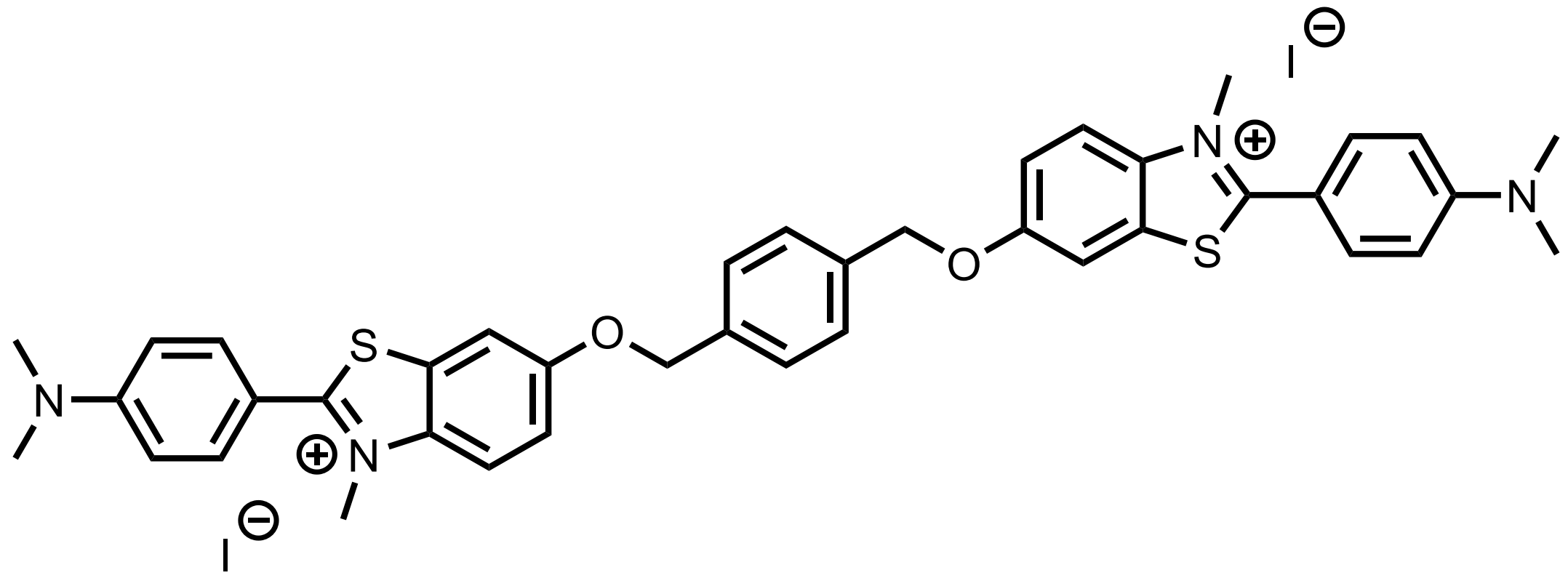

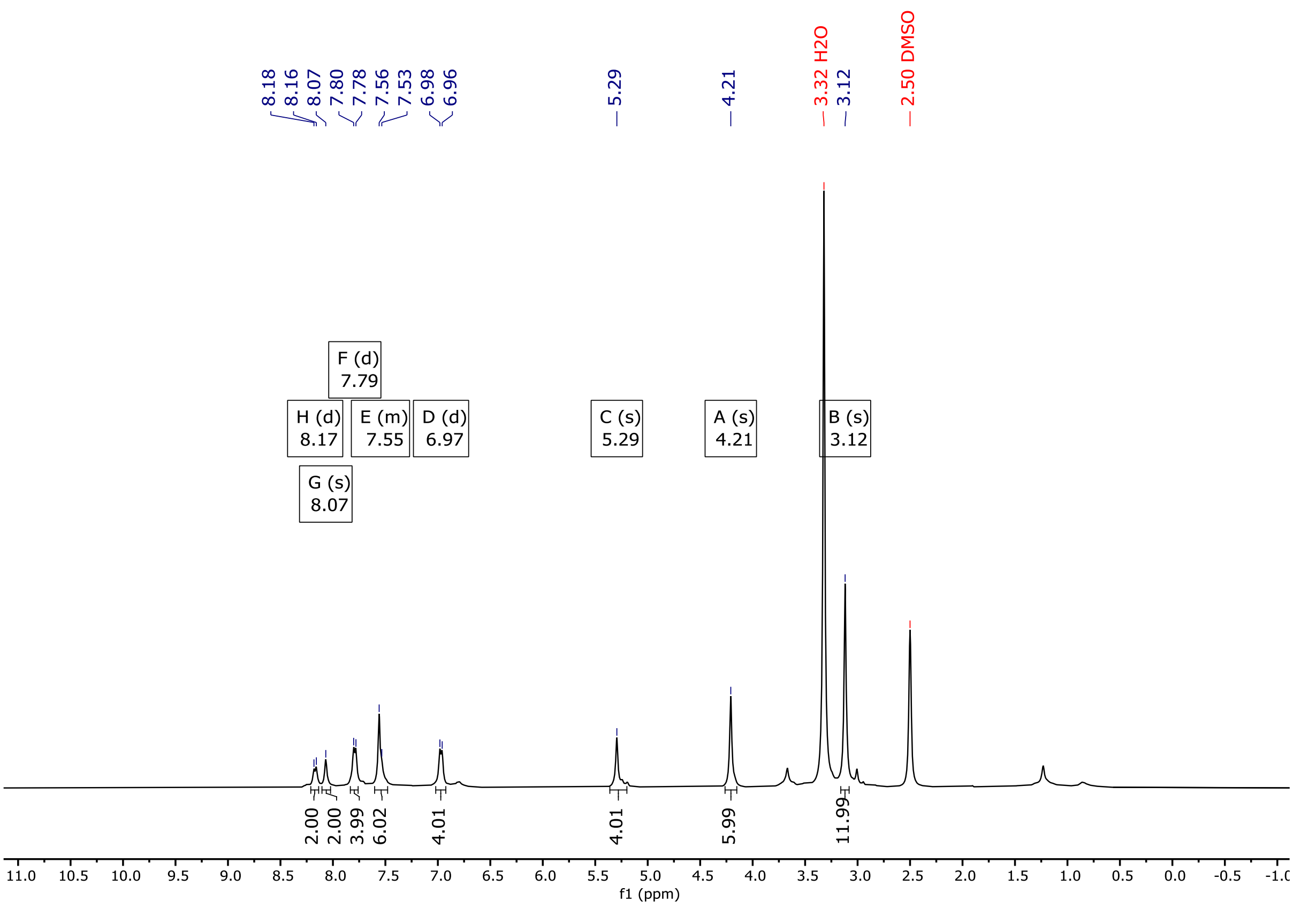

Figure S1. ^{1}H NMR (400 MHz, DMSO-d_6) spectra of **1**.

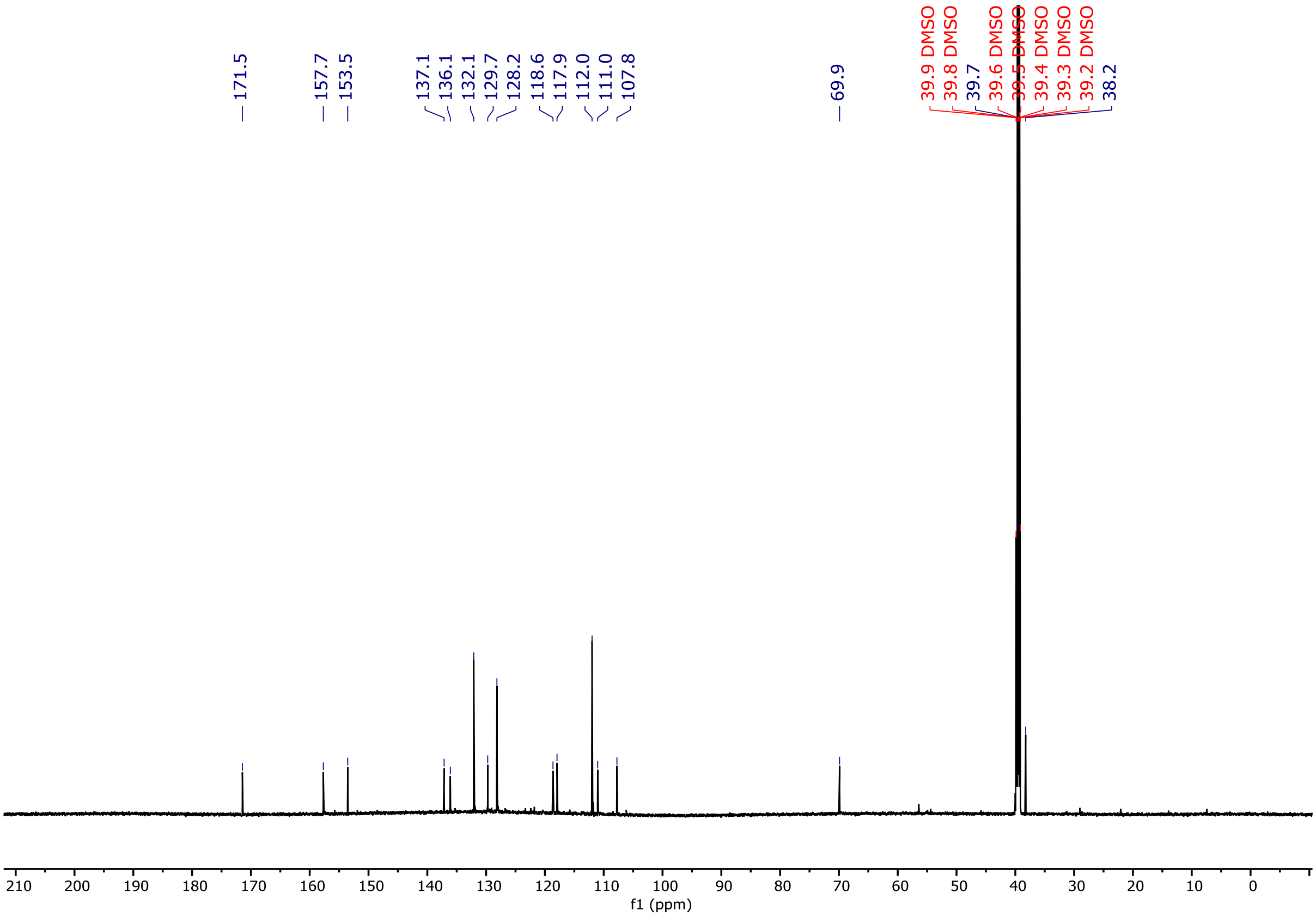

Figure S2. ^{13}C NMR (176 MHz, DMSO-d_6) spectra of **1**.

2,7-bis(2-(2-(2-hydroxyethoxy)ethoxy)ethyl)benzo[lmn][3,8]phenanthroline-1,3,6,8(2*H*,7*H*)-tetraone (**S2**)

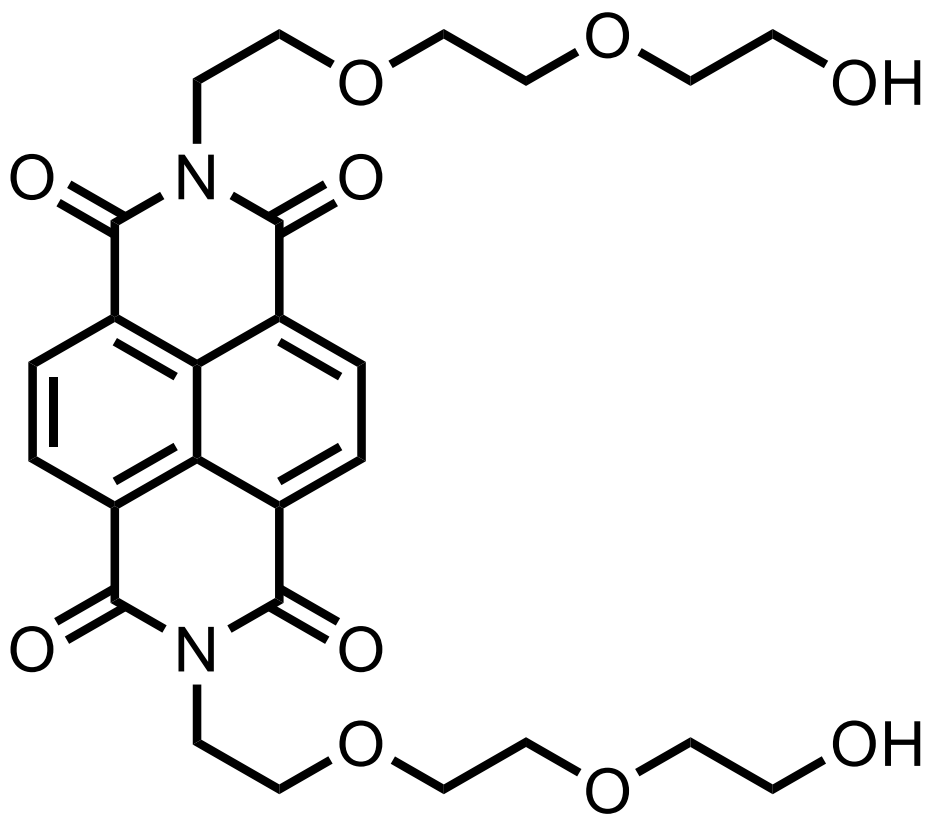

1,4,5,8-Naphthalenetetracarboxylic dianhydride (805 mg, 3.0 mmol, 1.0 equiv.) and 2-(2-aminoethoxy)ethanol (1.66 mL, 12.0 mmol, 4.0 equiv.) in NMP (9 mL) were heated to 110 °C for 2 h. The reaction mixture was cooled, poured into water, and **S2** was collected by filtration as a brown solid.

Yield: 1.451 g (2.73 mmol, 91%)

Aspect: brown solid

^{1}H NMR (400 MHz, $CDCl_3$) δ (ppm): 8.75 (s, 4H), 4.47 (t, *J* = 5.8 Hz, 4H), 3.87 (t, *J* = 5.7 Hz, 4H), 3.72 – 3.69 (m, 4H), 3.64 – 3.60 (m, 8H), 3.54 – 3.50 (m, 4H).

^{13}C NMR (101 MHz, $CDCl_3$) δ (ppm): 163.1, 131.2, 126.9, 126.7, 72.5, 70.6, 70.3, 68.0, 61.8, 39.8.

HRMS (ESI+): 531.2298 m/z: Calculated for $C_{26}H_{31}N_2O_{10}^+$ = 531.2174 $[M+H]^+$.

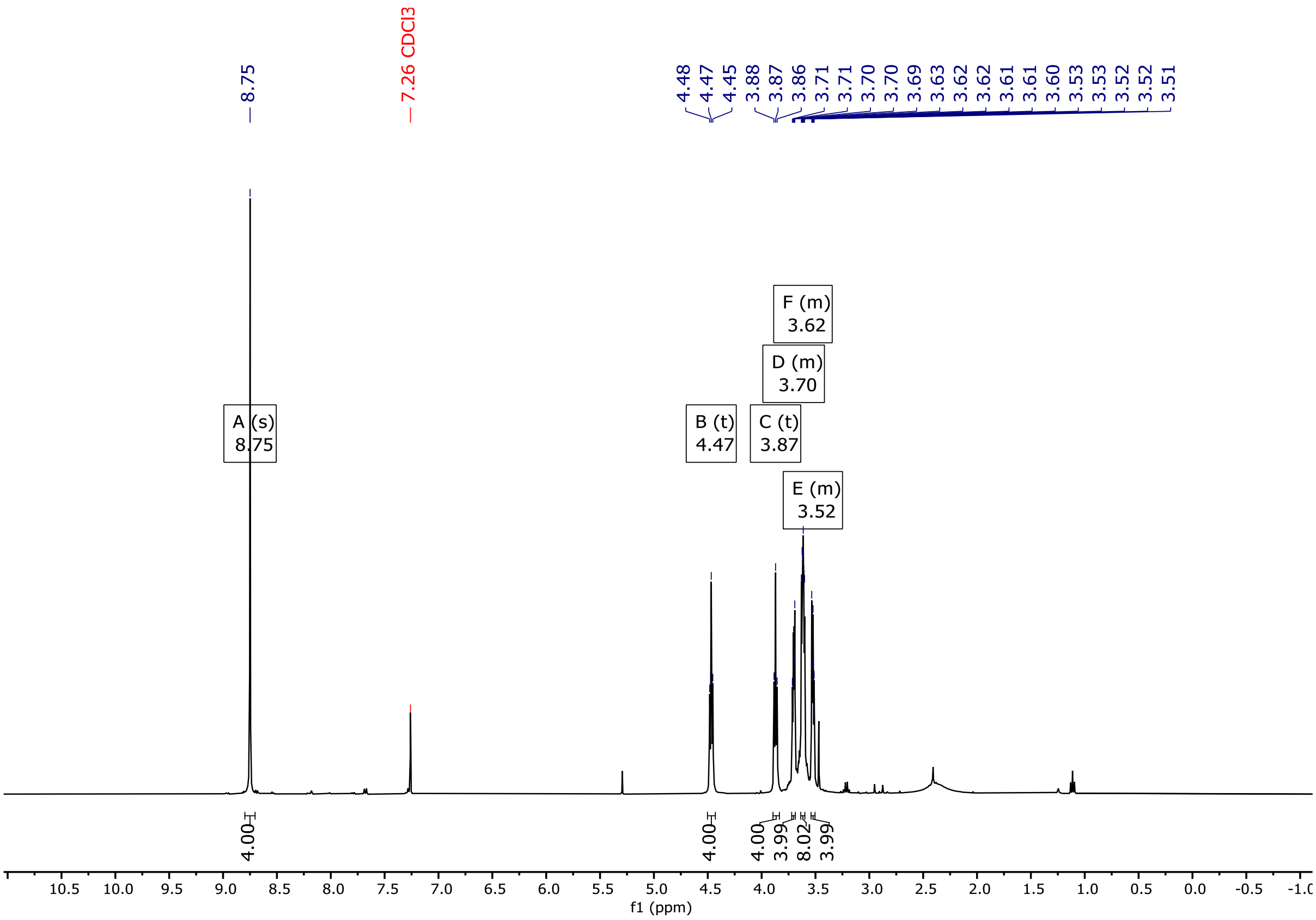

Figure S3. ^{1}H NMR (400 MHz, $CDCl_3$) spectra of **S2**.

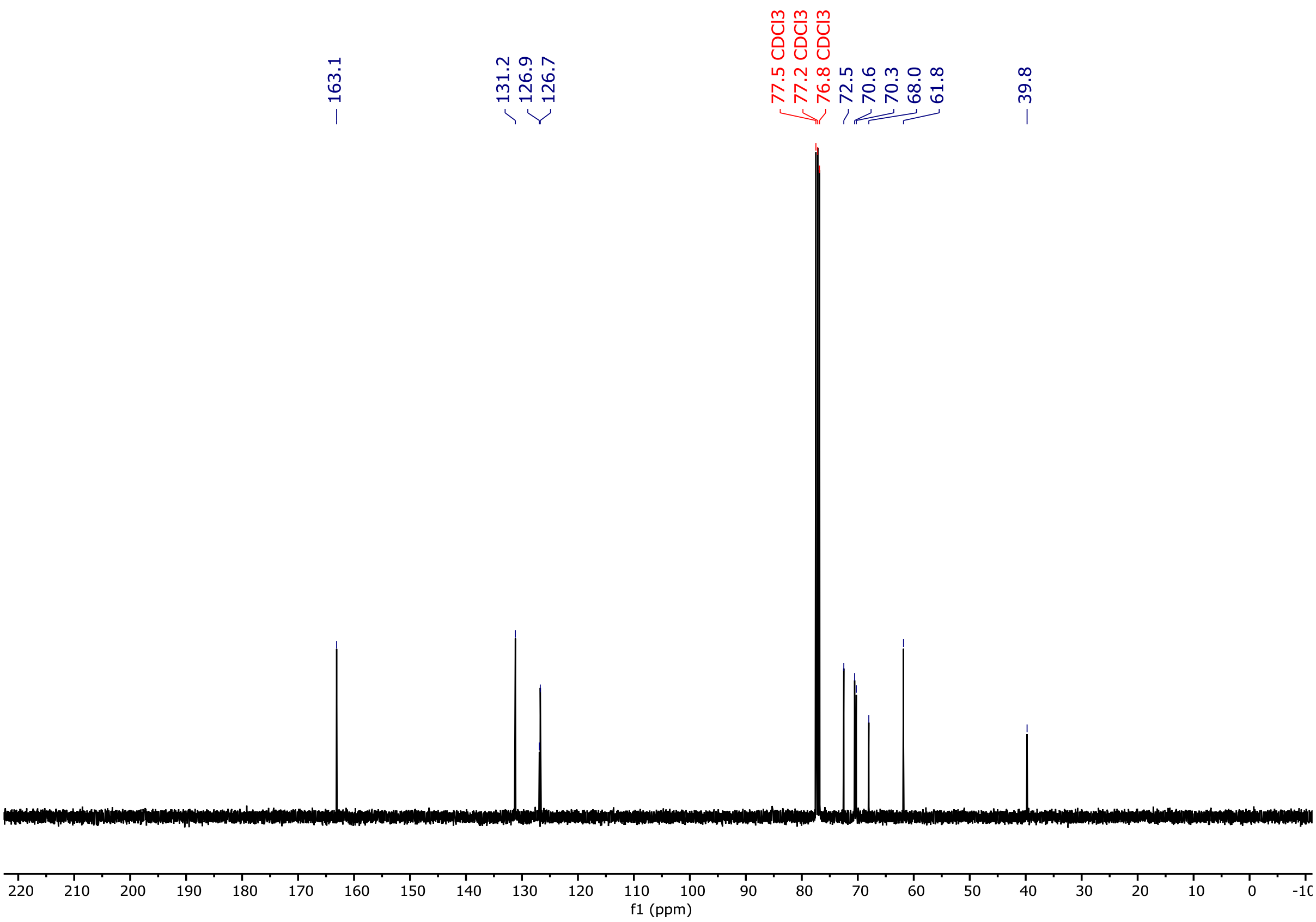

Figure S4. ^{13}C NMR (101 MHz, $CDCl_3$) spectra of **S2**.

2-(2-(2-(7-(2-(2-(2-hydroxyethoxy)ethoxy)ethyl)-1,3,6,8-tetraoxo-3,6,7,8-tetrahydrobenzo[lmn][3,8]phenanthrolin-2(1*H*)-yl)ethoxy)ethoxy)ethyl 4-methylbenzenesulfonate (**S3**)

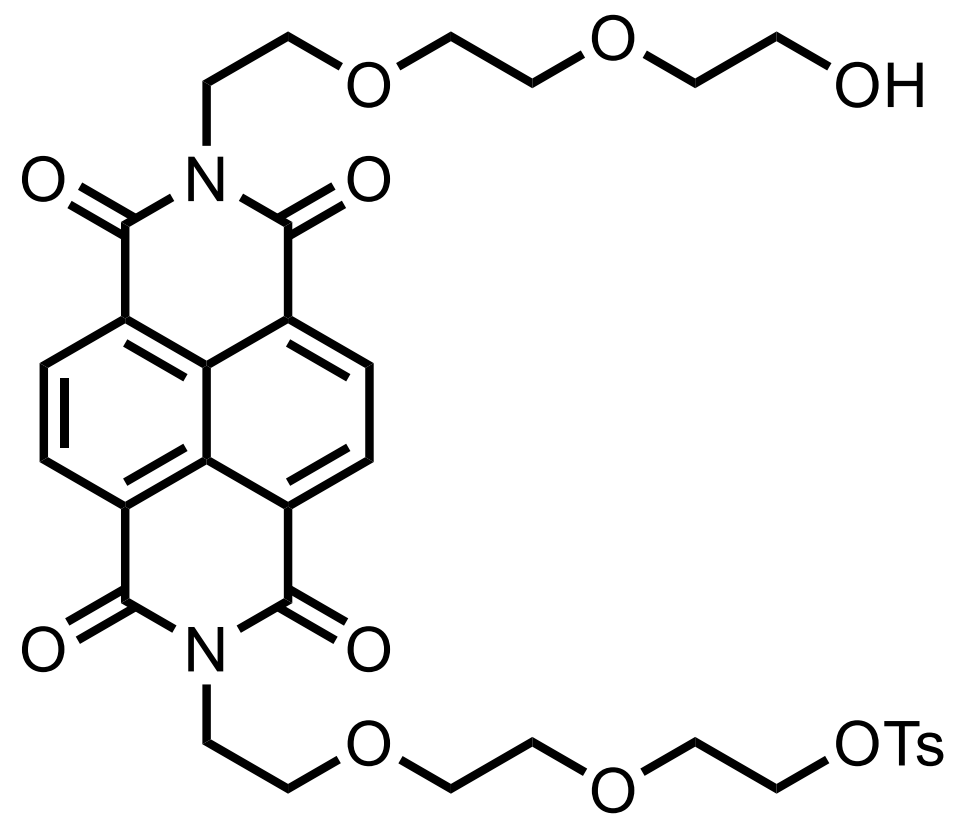

To a mixture of DMAP (2 mg, catalytic) and **S2** (212.2 mg, 0.40 mmol, 1.0 equiv.) in CH_2Cl_2 (2 mL) was added a solution of tosyl chloride (91.5 mg, 0.48 mmol, 1.2 equiv.) and triethylamine (223 µL, 1.6 mmol, 4.0 equiv.) in CH_2Cl_2 (2 mL) dropwise on ice. The reaction mixture was stirred for 1 h, and upon completion by LCMS, 10w/v% NH_4Cl (10 mL) was added and the mixture was warmed to room temperature. The aqueous layer was washed with CH_2Cl_2 (3 x 20 mL) and the combined organic extracts were washed with brine (20 mL), dried over Na_2SO_4, and the solvent removed *in vacuo*. The crude product was then purified by flash silica column chromatography (PE/EtOAc) to afford the monotosylated **S4** (54.9 mg, 80 µmol, 20%) as a yellow-orange solid, and the ditosylated product (177.3 mg, 211 µL, 53%).

Yield: 54.9 mg (80 µmol, 20%)

Aspect: yellow-orange solid

^{1}H NMR (400 MHz, CDCl$_3$) δ (ppm): 8.71 (d, *J* = 1.3 Hz, 4H), 7.72 (dt, *J* = 8.4, 1.8 Hz, 2H), 7.32 – 7.29 (m, 2H), 4.43 (dt, *J* = 13.3, 5.8 Hz, 4H), 4.05 – 4.01 (m, 2H), 3.86 (t, *J* = 5.8 Hz, 2H), 3.80 (t, *J* = 5.8 Hz, 2H), 3.71 – 3.67 (m, 2H), 3.63 – 3.58 (m, 8H), 3.52 (dt, *J* = 5.9, 3.2 Hz, 4H), 2.42 (s, 3H).

^{13}C NMR (101 MHz, CDCl$_3$) δ (ppm): 163.0, 162.9, 144.9, 133.0, 131.1, 131.1, 129.9, 128.0, 126.8, 126.8, 126.7, 126.6, 72.5, 70.8, 70.5, 70.2, 70.2, 69.2, 68.7, 68.0, 67.9, 61.8, 39.7, 39.6, 21.7.

HRMS (ESI+): 685.2063 m/z: Calculated for $C_{33}H_{37}N_2O_{12}S^+$ = 685.2062 $[M+H]^+$. 707.1892 m/z: Calculated for $C_{33}H_{36}N_2O_{12}SNa^+$ = 707.1882.

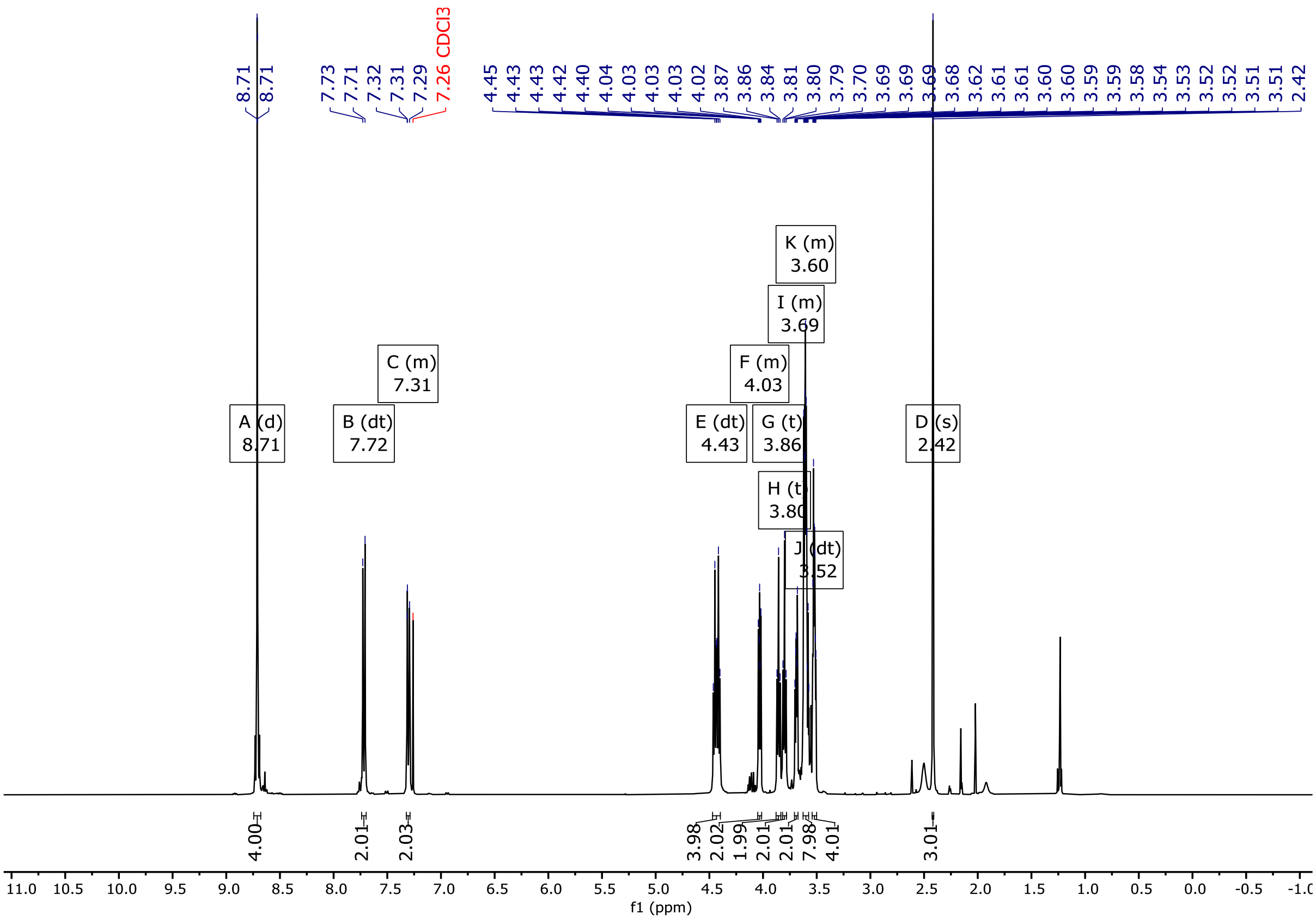

Figure S5. ^{1}H NMR (400 MHz, $CDCl_3$) spectra of **S3**.

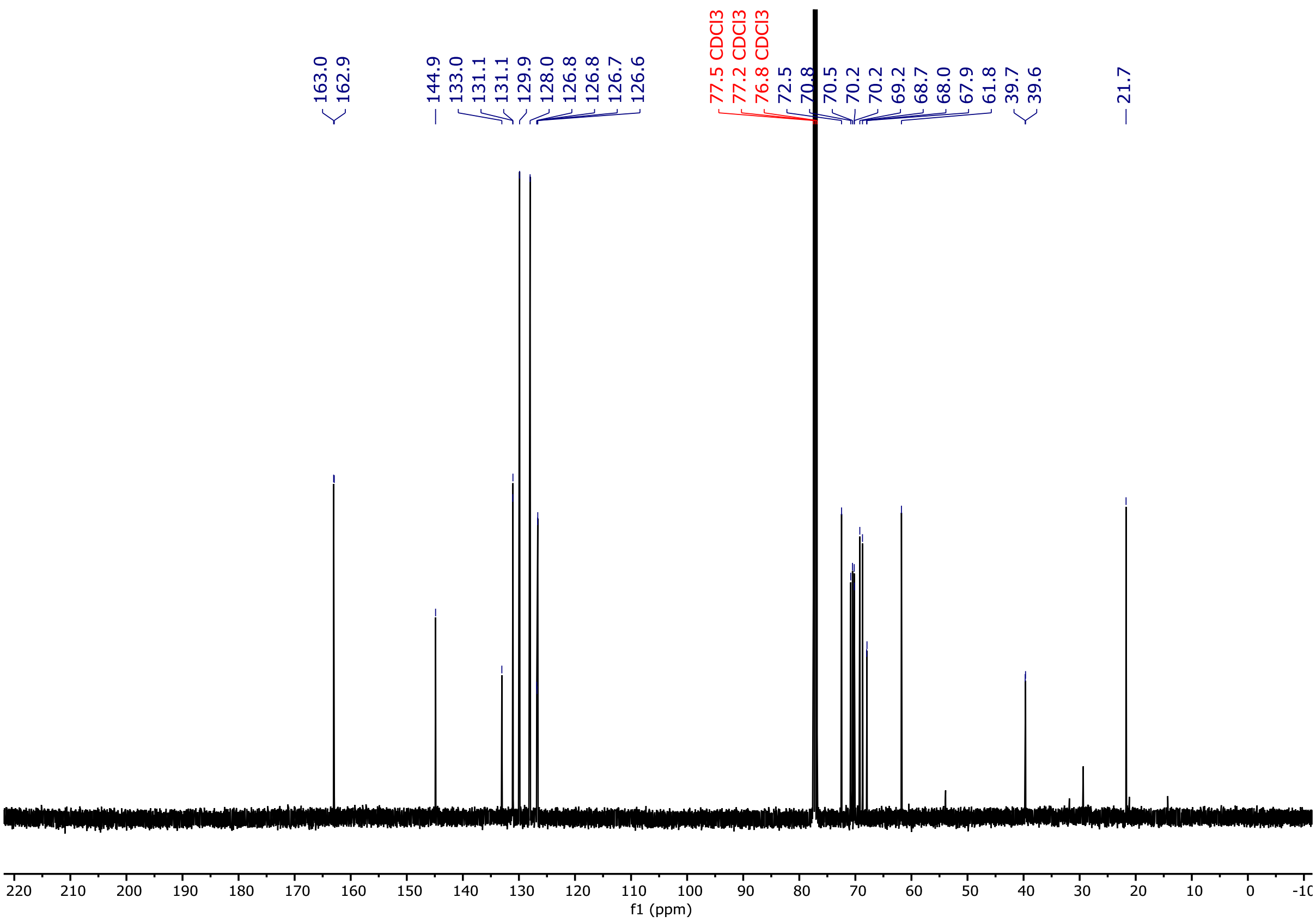

Figure S6. ^{13}C NMR (101 MHz, $CDCl_3$) spectra of **S3**.

2-(2-(2-(2-((2-(4-(dimethylamino)phenyl)benzo[*d*]thiazol-6-yl)oxy)ethoxy)ethoxy)ethyl)-7-(2-(2-(2-hydroxyethoxy)ethoxy)ethyl)benzo[lmn][3,8]phenanthroline-1,3,6,8(2*H*,7*H*)-tetraone (**S4**)

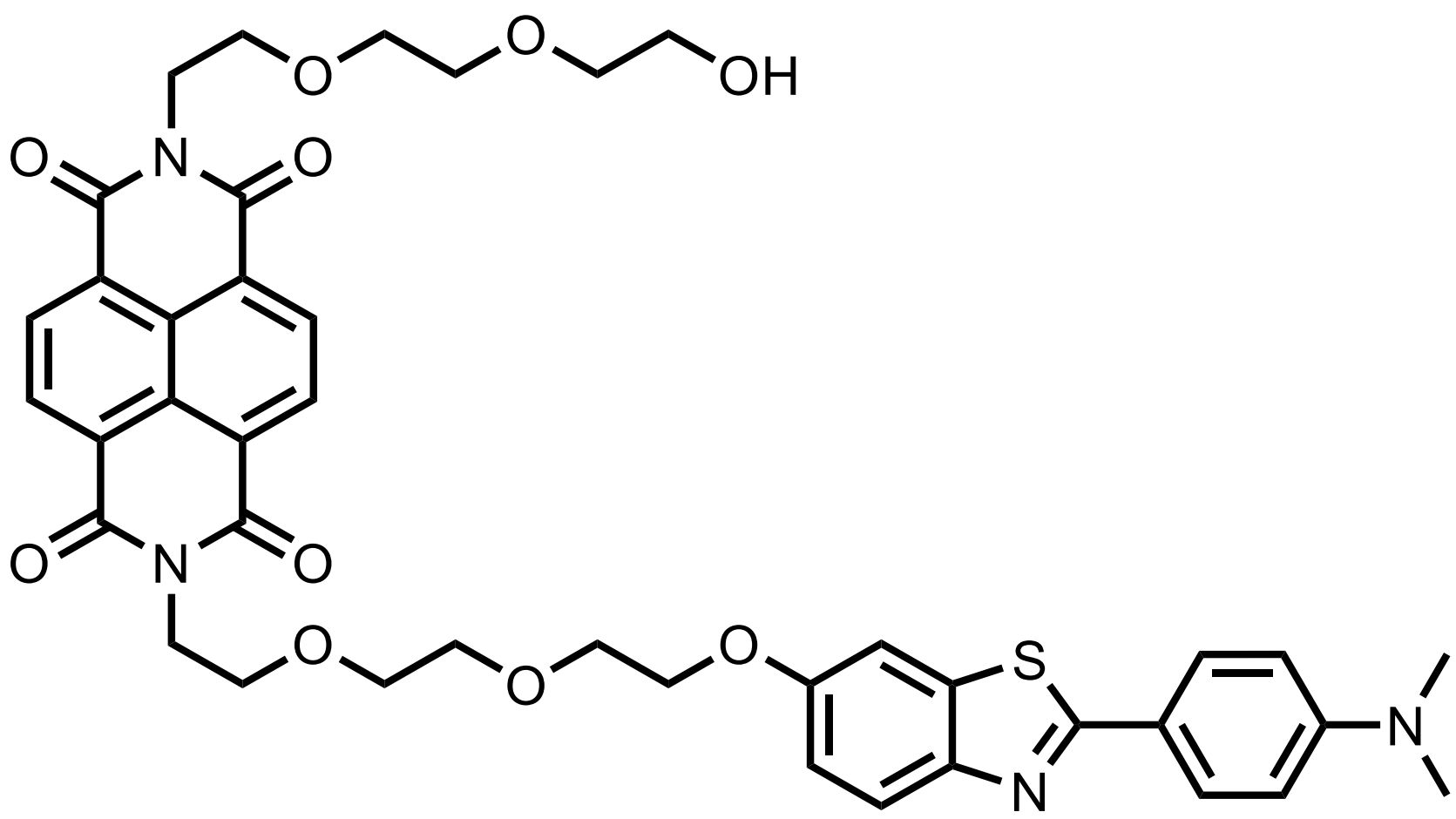

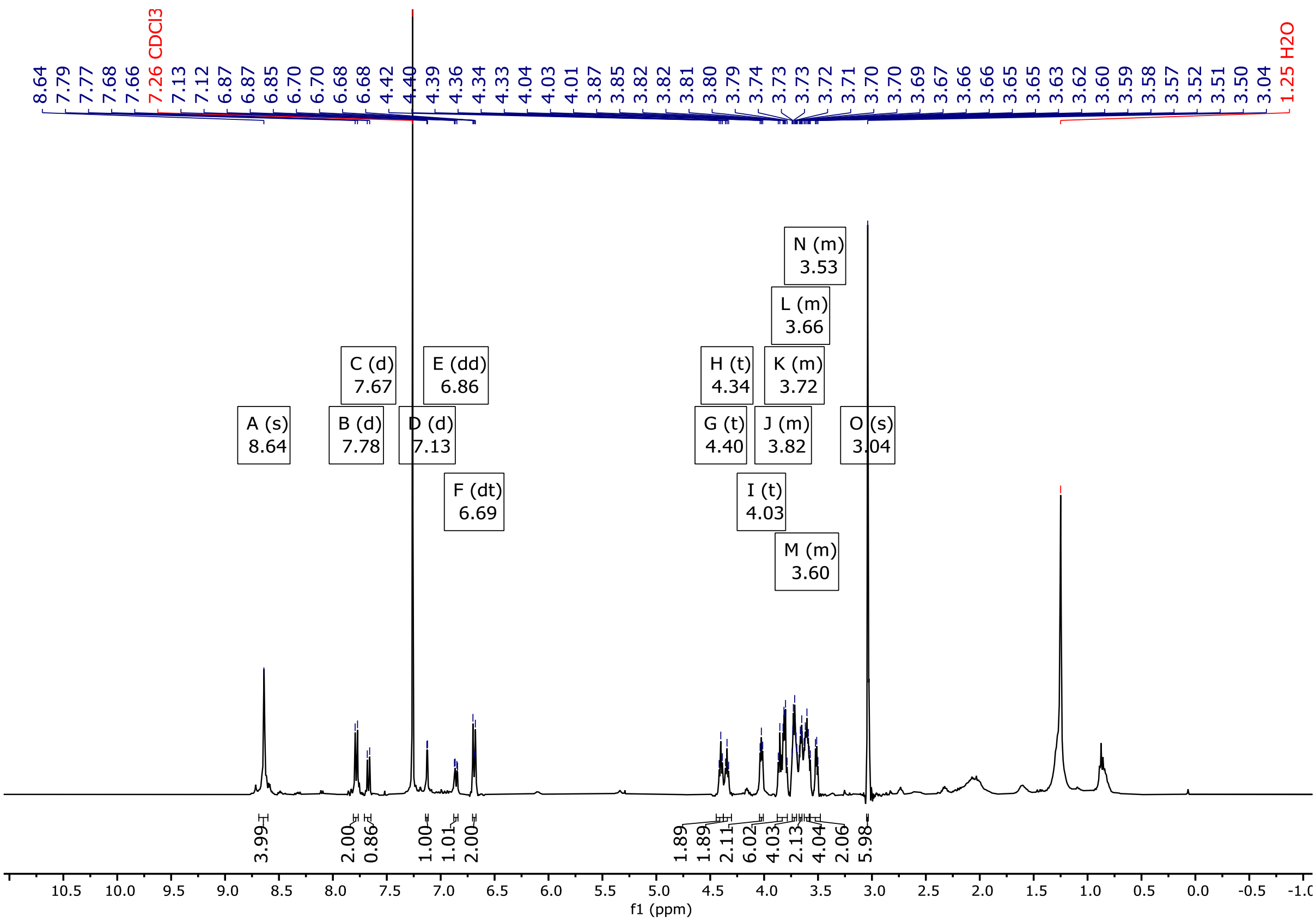

Figure S7. ^{1}H NMR (400 MHz, $CDCl_3$) spectra of **S4**.

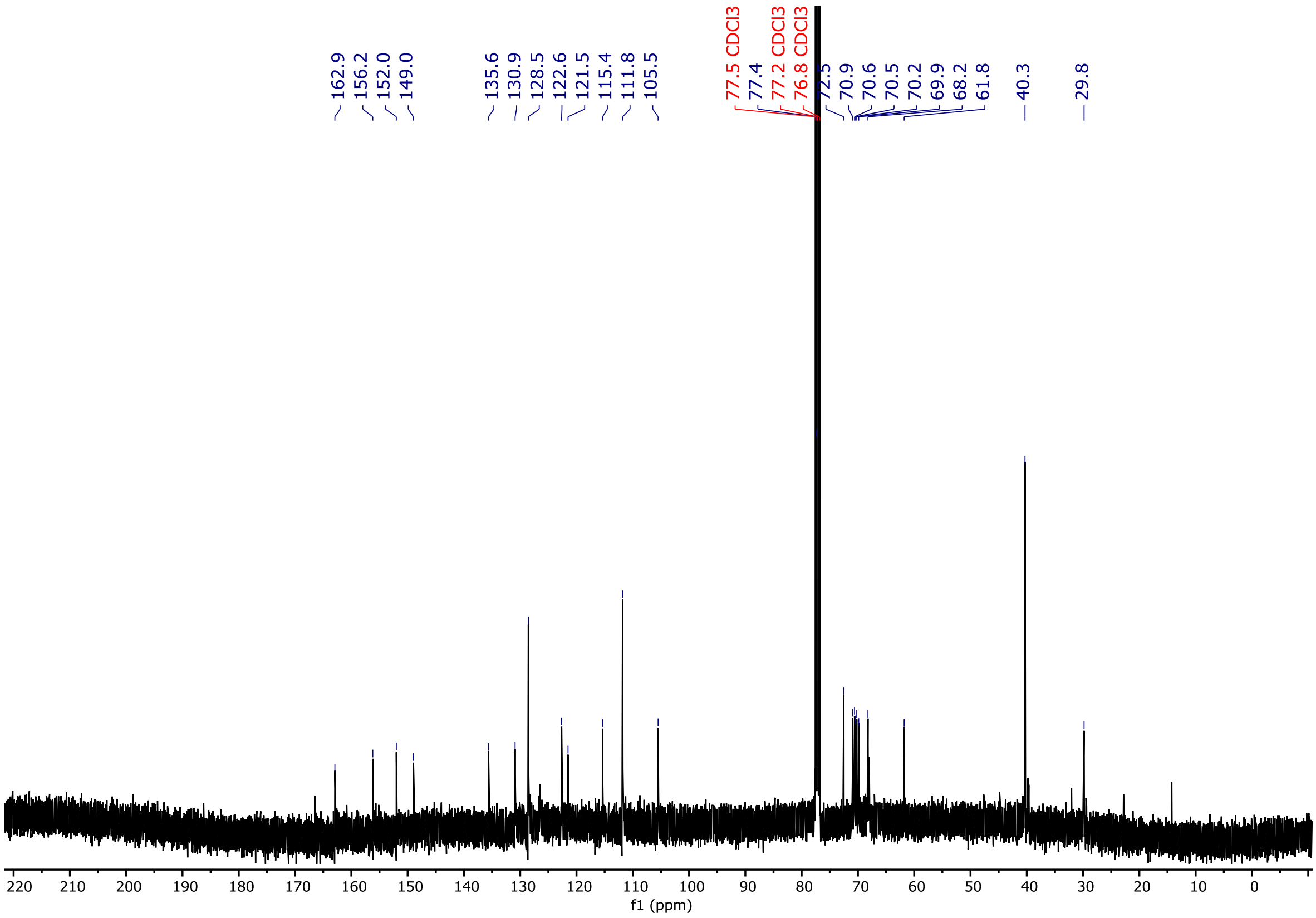

Figure S8. ^{13}C NMR (101 MHz, $CDCl_3$) spectra of **S4**.

2-(4-(dimethylamino)phenyl)-6-(2-(2-(2-(7-(2-(2-(2-hydroxyethoxy)ethoxy)ethyl)-1,3,6,8-tetraoxo-3,6,7,8-tetrahydrobenzo[lmn][3,8]phenanthrolin-2(1*H*)-yl)ethoxy)ethoxy)ethoxy)-3-methylbenzo[*d*]thiazol-3-ium iodide (**2**)

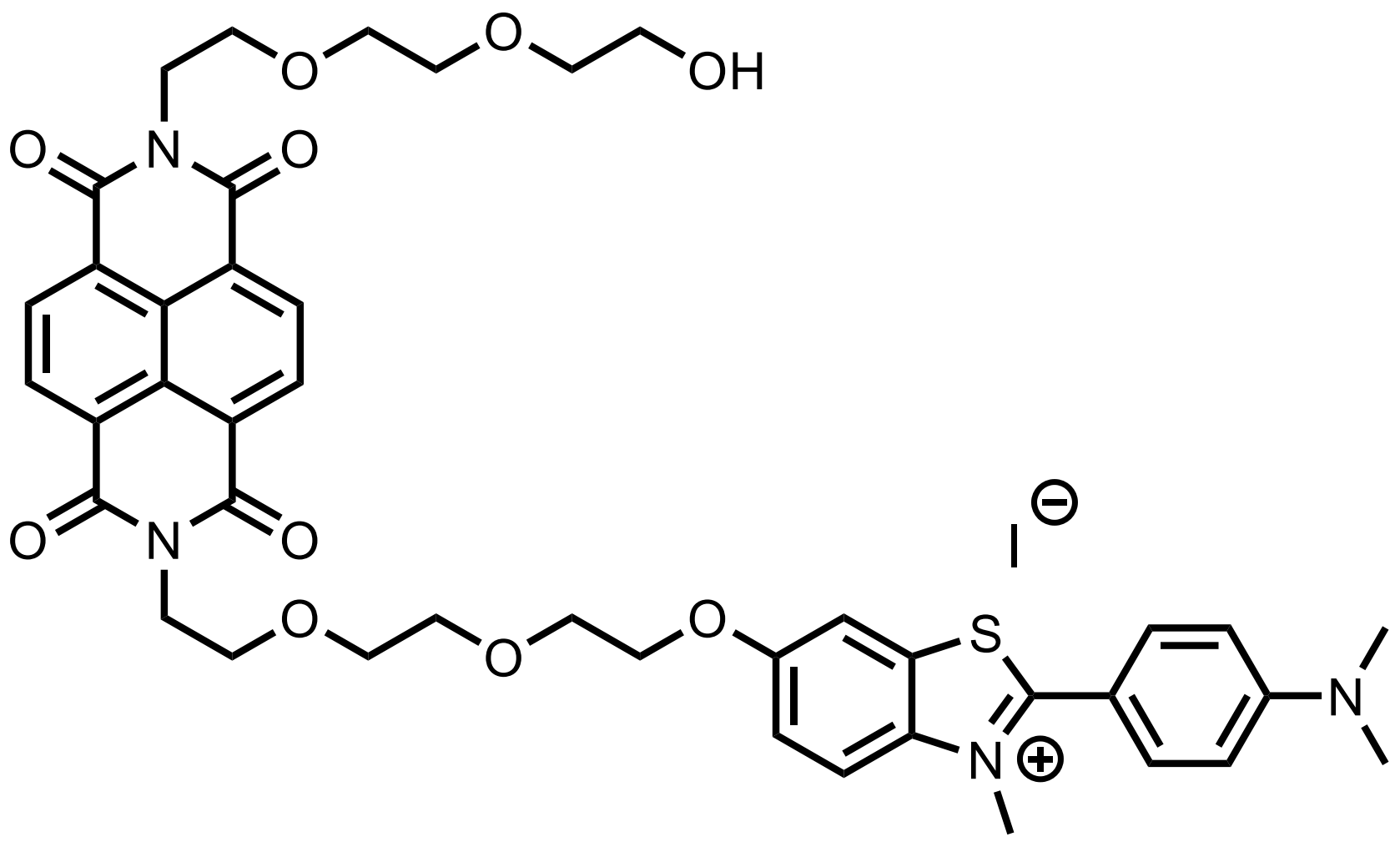

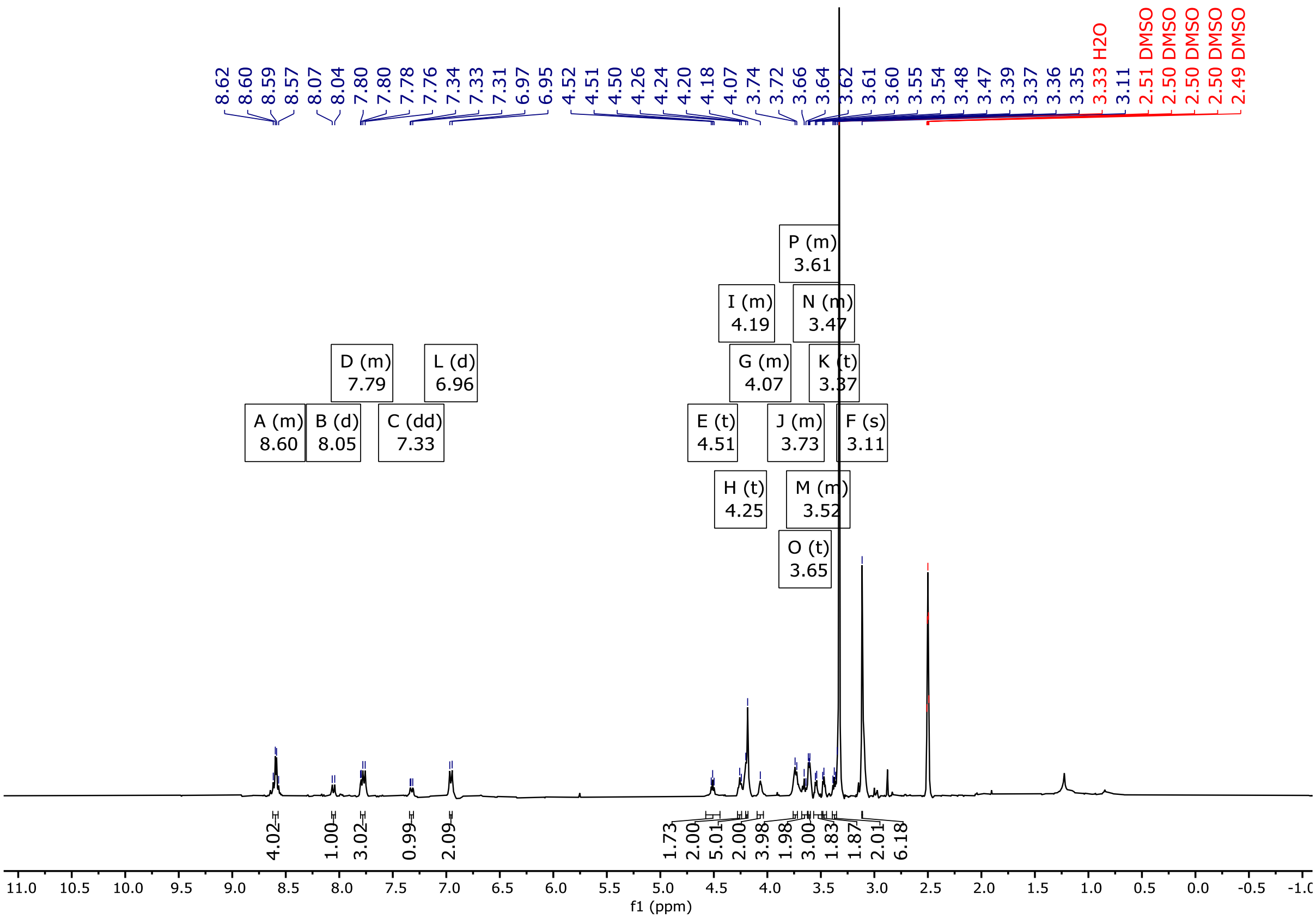

Figure S9. ^{1}H NMR (400 MHz, DMSO-d_6) spectra of **S4**.

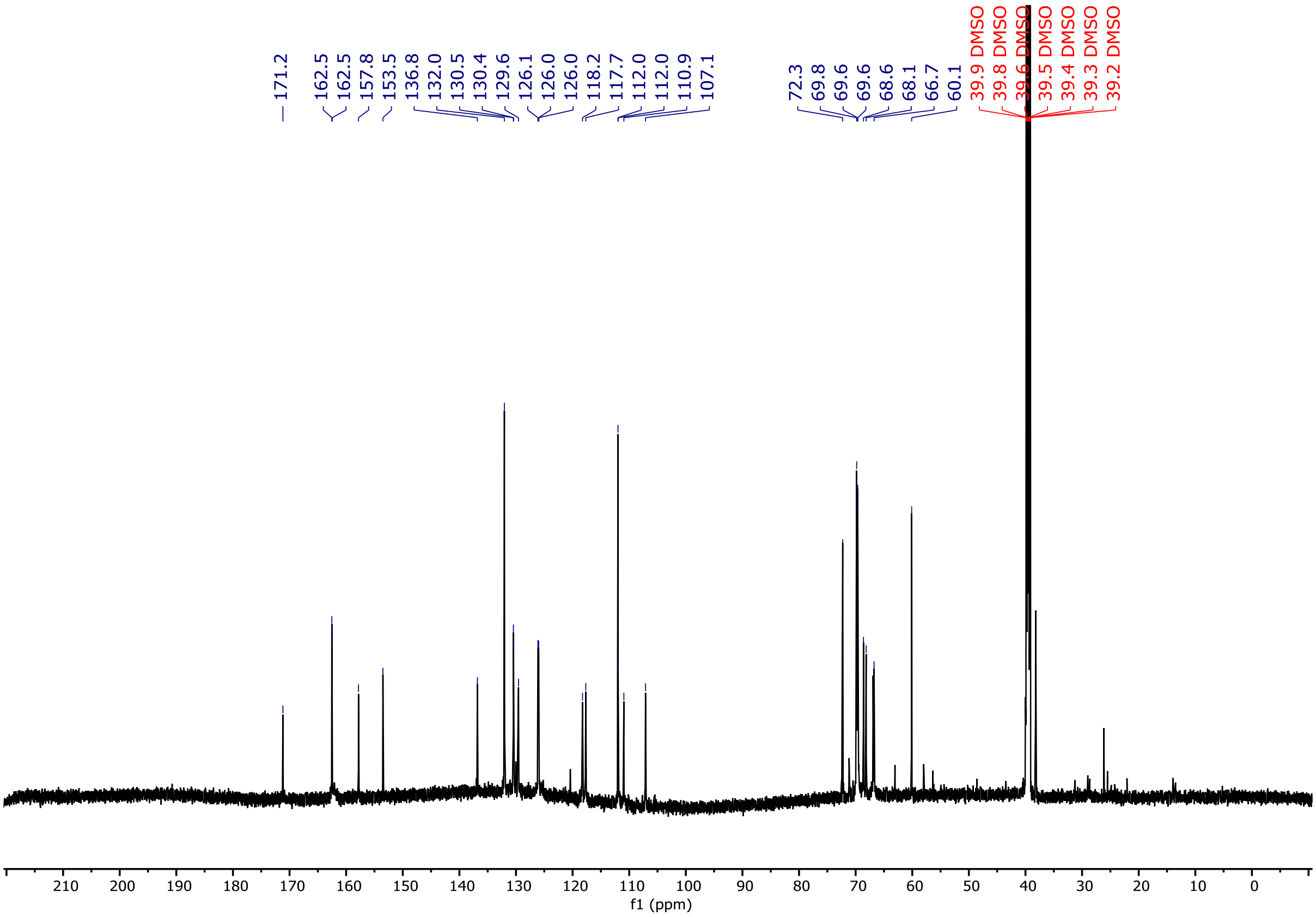

Figure S10. ^{13}C NMR (176 MHz, DMSO-d_6) spectra of **S4**.

UV-Visible Characterisation

Ligand stock solutions in DMSO (10 mM) were diluted in ethanol to obtain a 50 μM solution in a quartz fluorescence cuvette (Hellma Analytics, 1 cm pathlength). UV-visible spectra were obtained using an Agilent Cary 60 UV-vis spectrophotometer with Cary WinUV software using a scan rate of 600 nm/min, a data interval of 1.0 nm and an averaging time of 0.10 at 25°C.

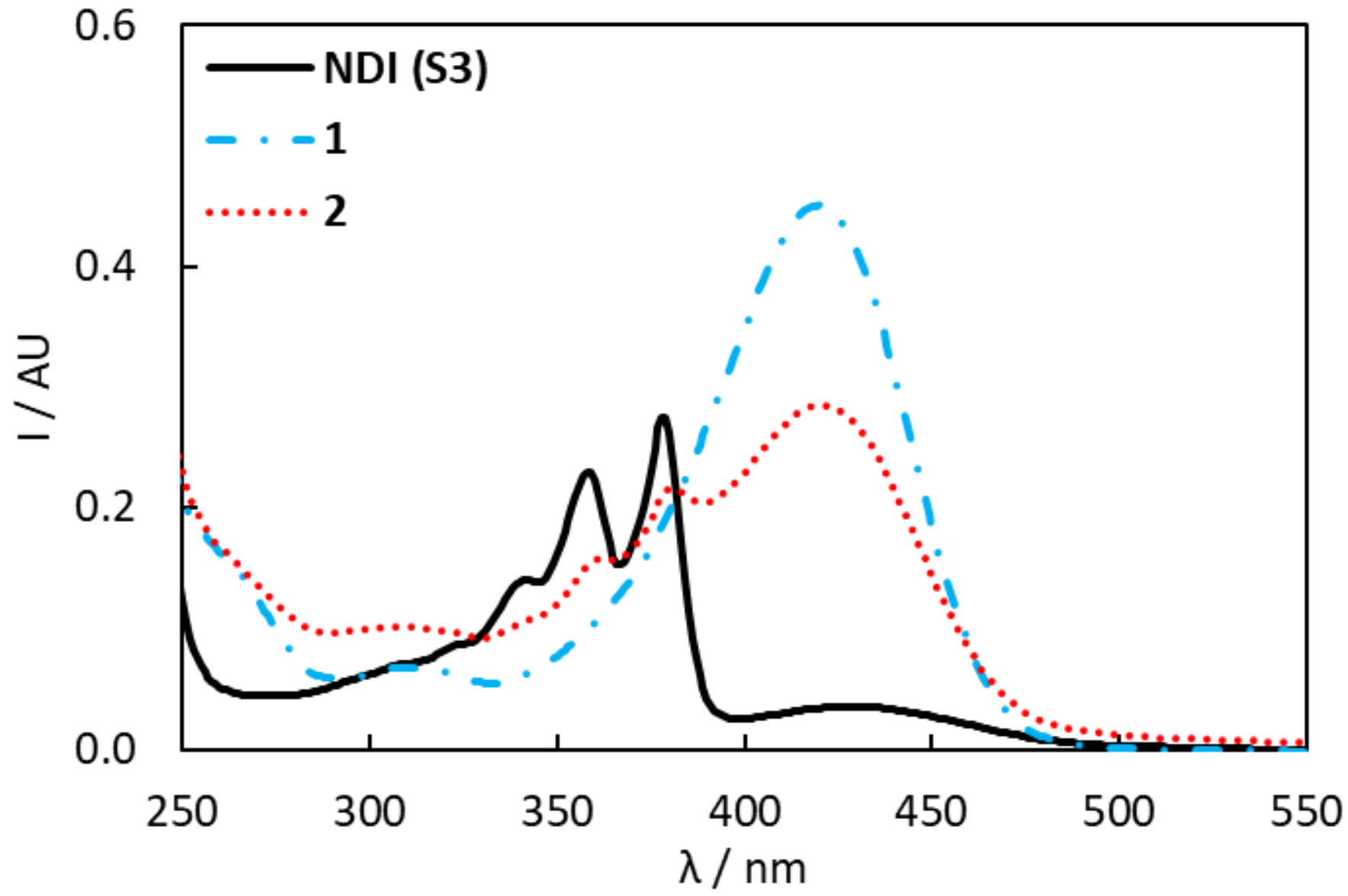

Figure S11. UV-vis spectra of **S2** (black line), **1** (blue dash-dotted line), and **2** (red dotted line) (50 μM in EtOH).

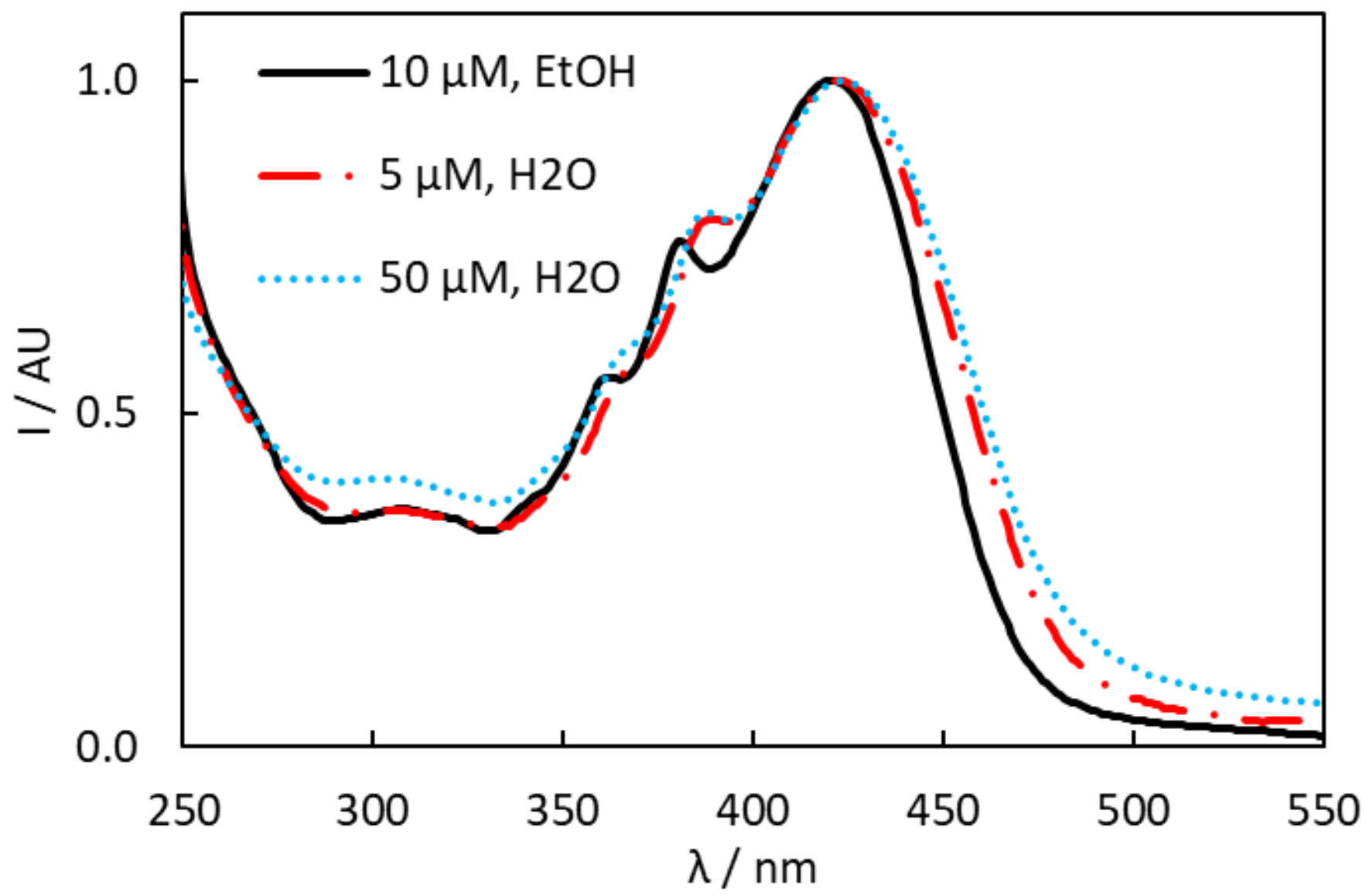

Figure S12. UV-vis spectra of **2** in EtOH (black line, 50 μM in EtOH), and H_2O (red dash-dotted line: 5 μM; blue dotted line: 50 μM).

Fluorescence Characterisation

Fluorescence excitation and emission spectra were obtained using an Agilent Cary Eclipse Fluorescence Spectrophotometer with a scan rate of 600 nm/min, a data interval of 1.0 nm, an averaging time of 0.10. 20 nm excitation and emission slits, and medium PMT voltage at 25°C.

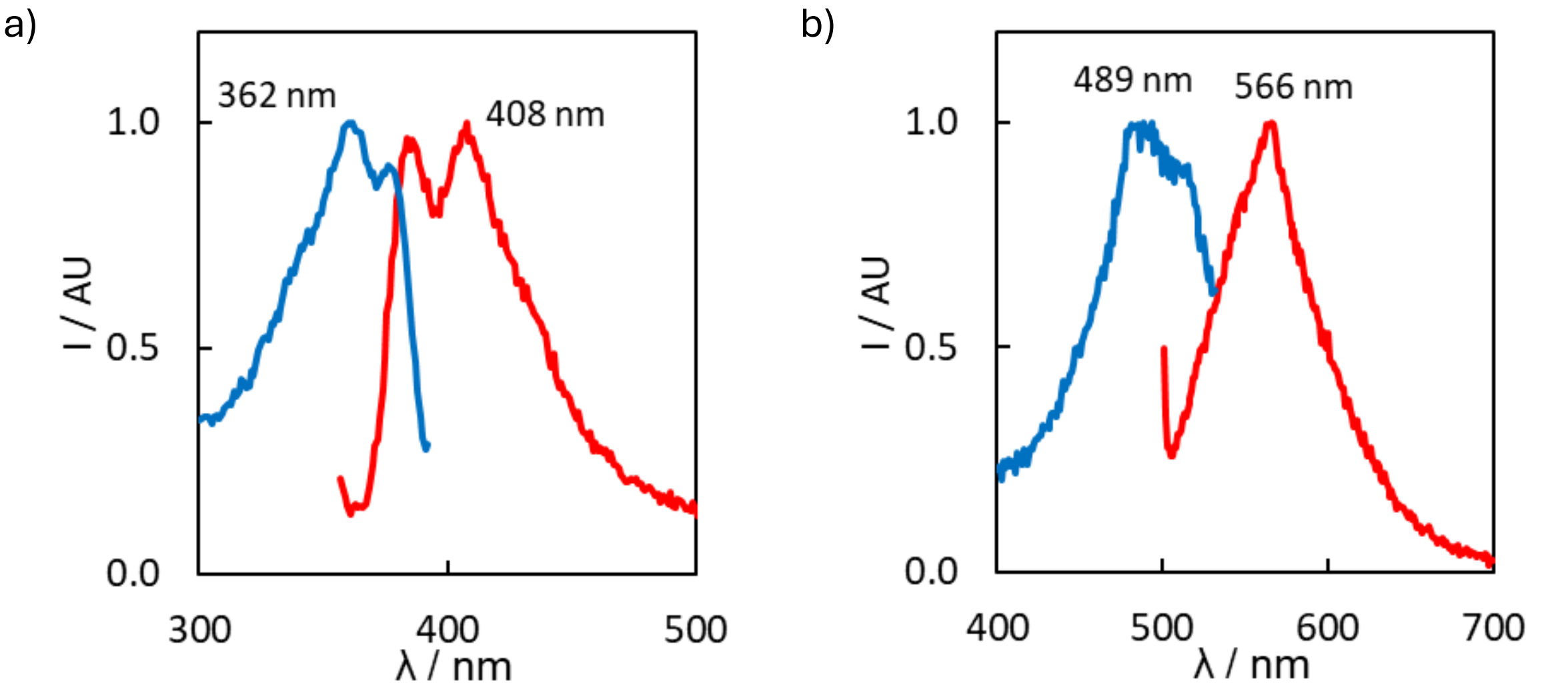

Figure S13. Fluorescence spectra of **S2** (10 μM) in EtOH (a: λ_{ex} = 342, λ_{em} = 407; b: λ_{ex} = 486, λ_{em} = 557).

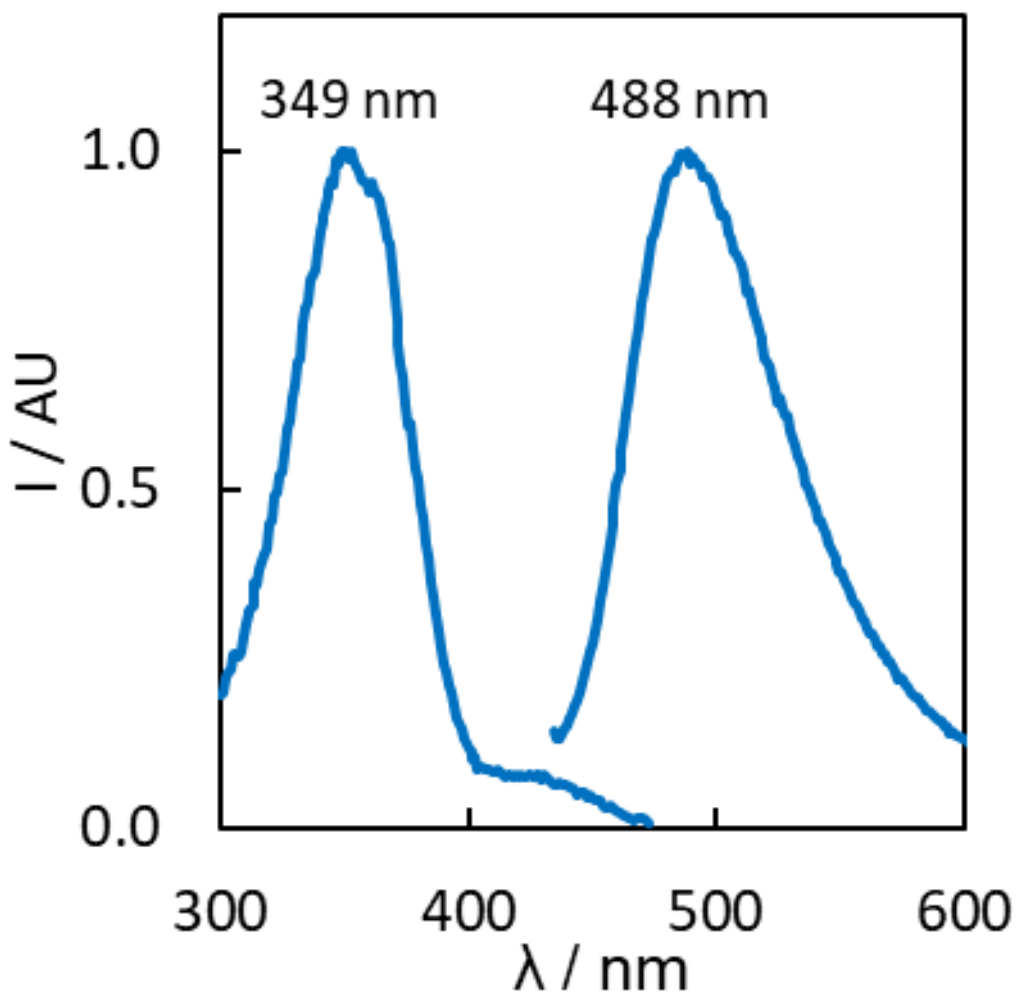

Figure S14. Fluorescence spectra of **1** (10 μM) in EtOH (λ_{ex} = 440, λ_{em} = 488).

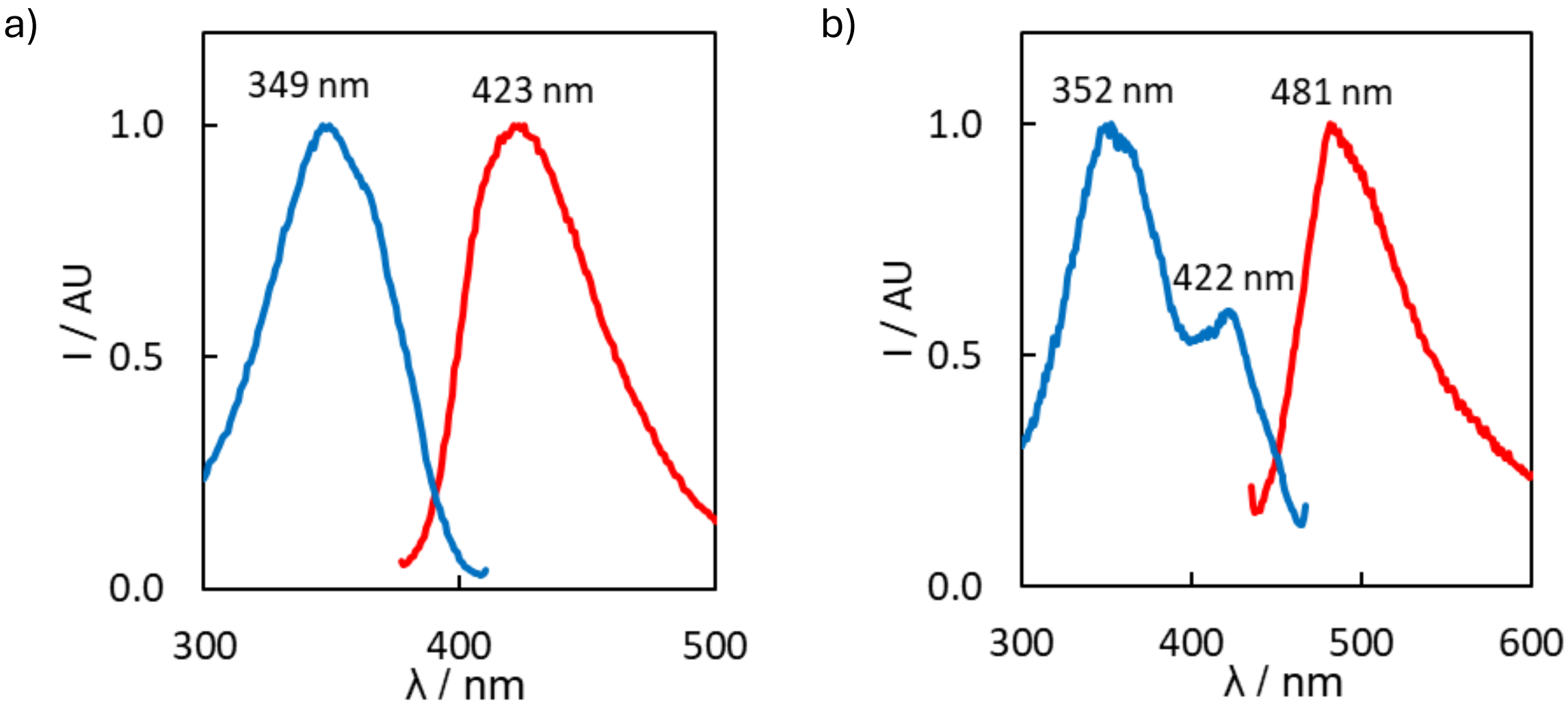

Figure S15. Fluorescence spectra of **2** (10 μM) in EtOH (a: λ_{ex} = 362, λ_{em} = 425; b: λ_{ex} = 420, λ_{em} = 482).

Additional Scatchard Plots and Hill Plots

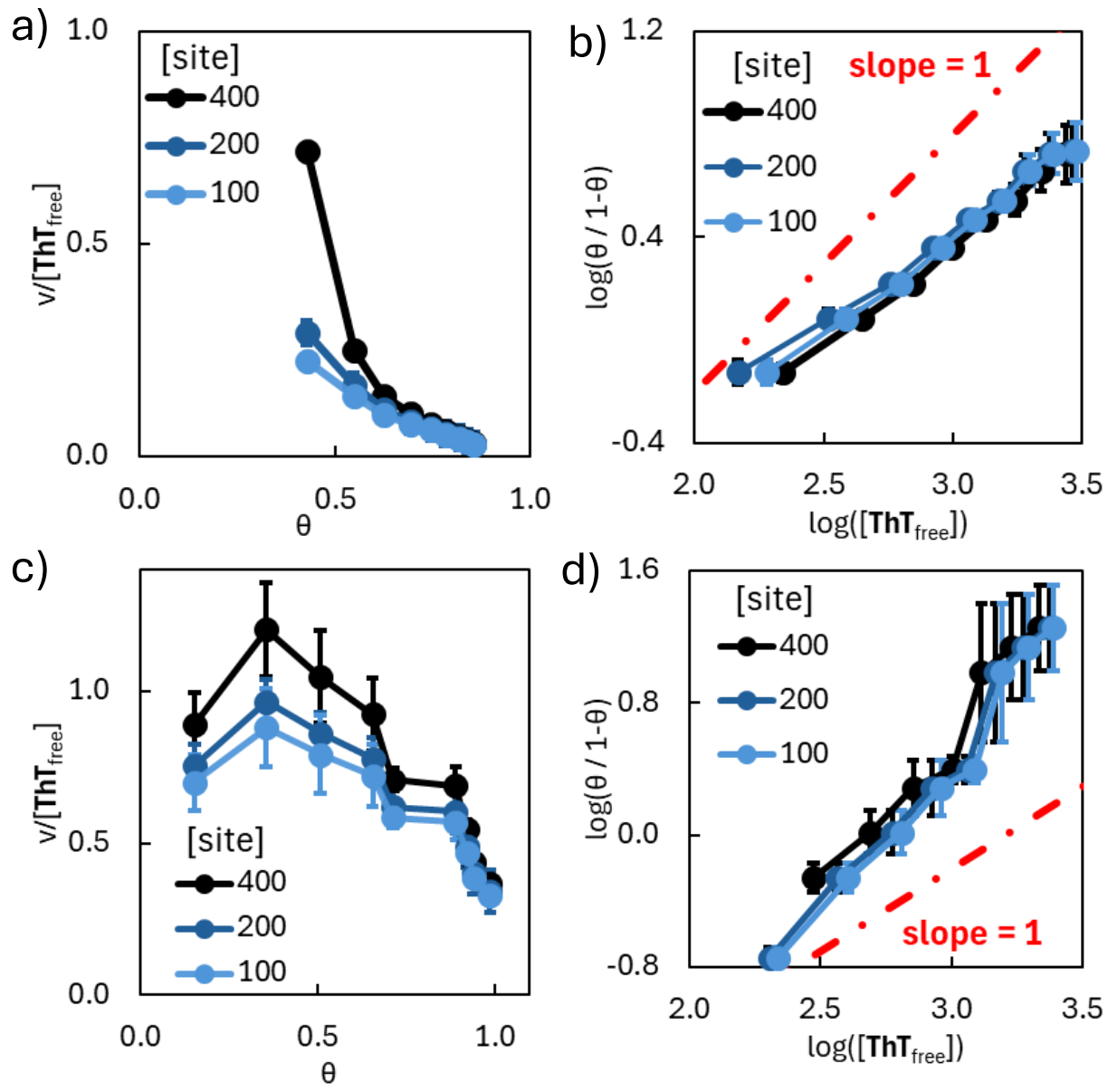

Figure S16. Scatchard and Hill plots for the binding of **ThT** (λ_{ex} = 440 nm, λ_{em} = 483 nm) to (a,b) PD fibrils (500 nM), and (c,d) f65 fibrils. Data are calculated for the different lattice site concentrations (in nM) shown. Data points are the average of at least three experimental measurements with 95% confidence intervals shown.

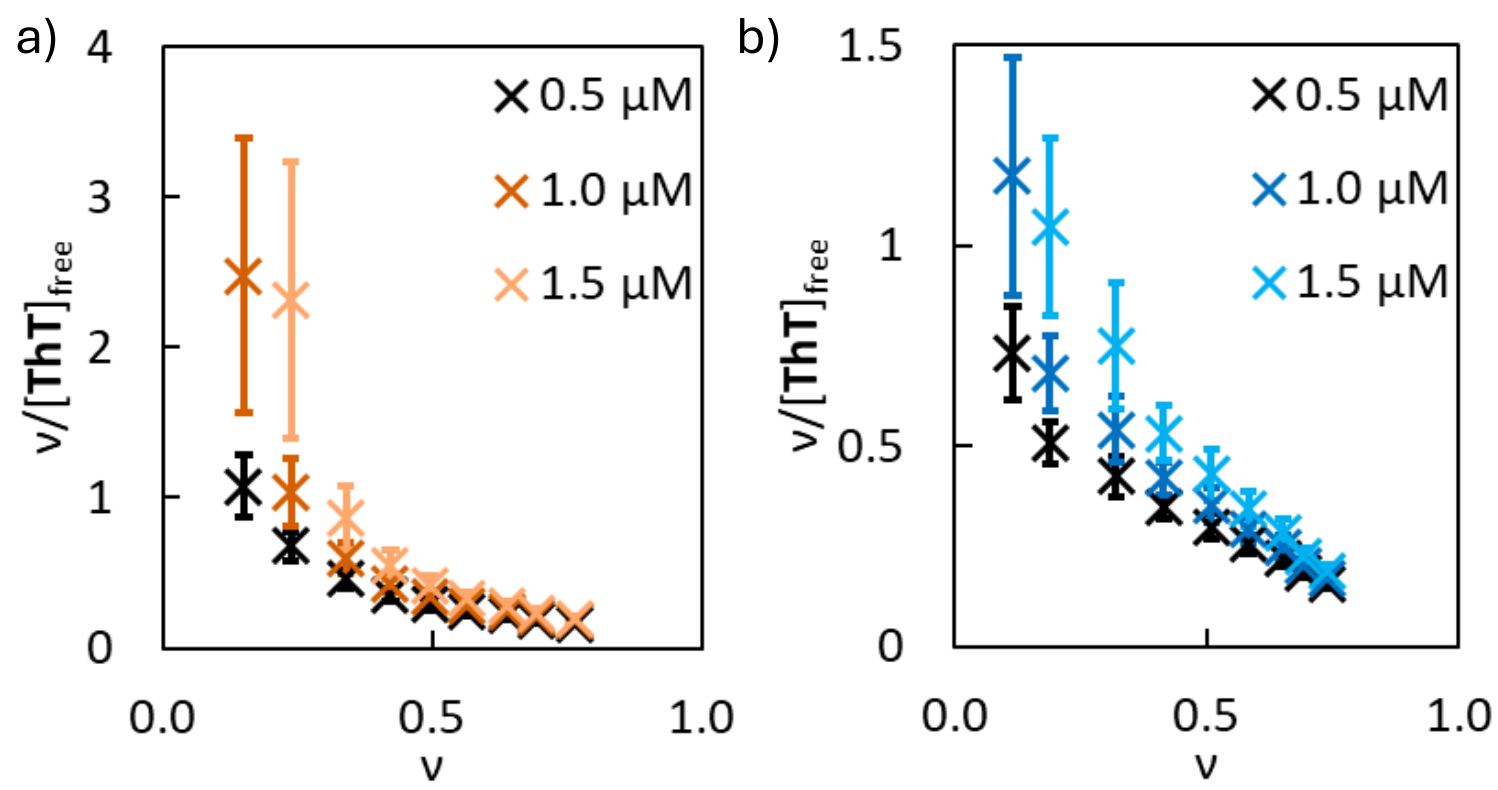

Figure S17. Scatchard plots for **ThT** (λ_{ex} = 440 nm) binding to (a) αSyn-Tris fibrils and (b) αSyn-PBS fibrils (500 nM) in 1xPBS (pH 7.4). Data are calculated for the different lattice site concentrations shown. Data points are the average of at least three experimental measurements with 95% confidence intervals shown.

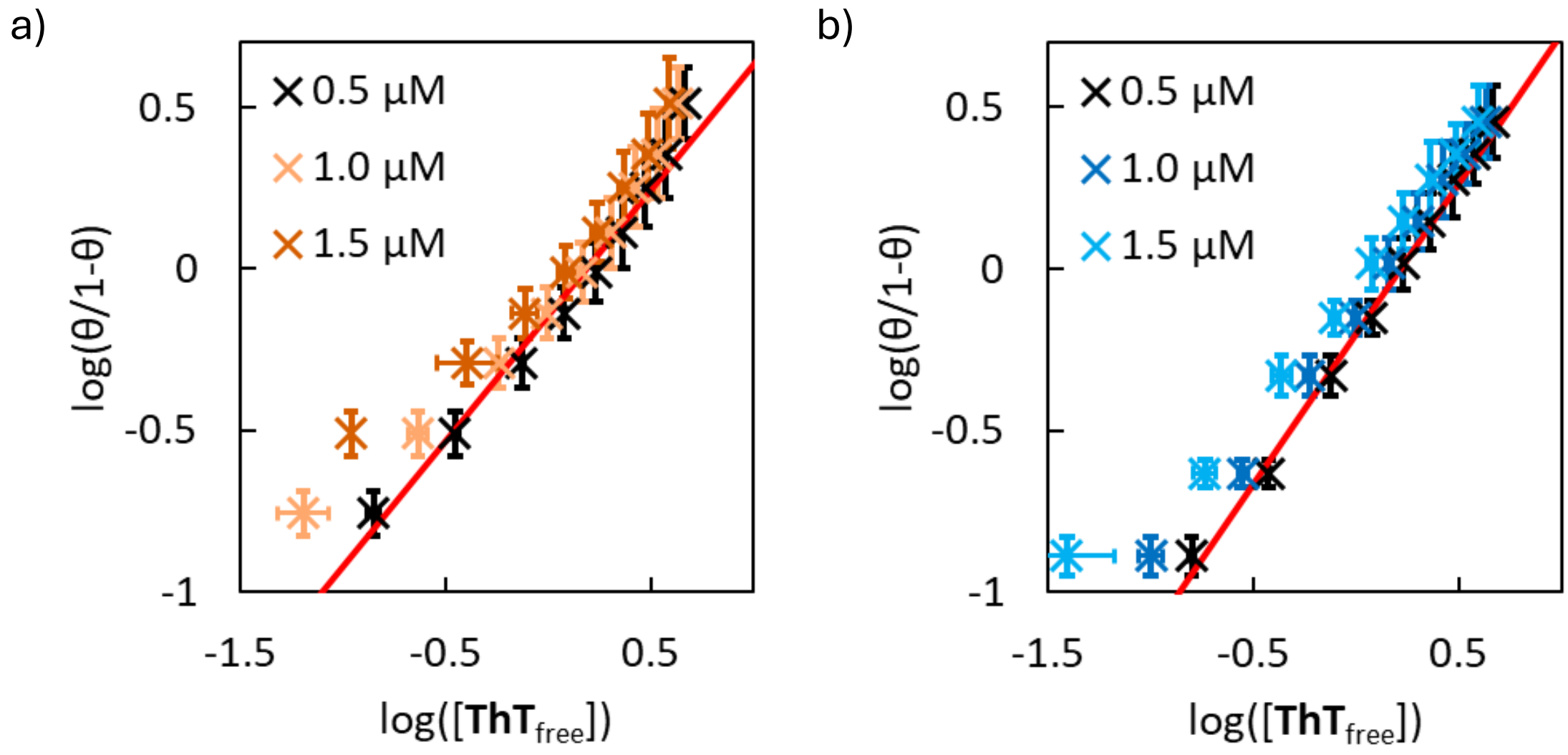

Figure S18. Hill plots for binding assays of **ThT** (λ_{ex} = 440 nm) with (a) αSyn-Tris and αSyn-PBS fibrils (500 nM) in 1xPBS (pH 7.4). Red line shows weighted linear regression to 0.5 µM data (a: y = (0.8 ± 0.1)x - (0.14 ± 0.06); b: y = (0.9 ± 0.1)x - (0.20 ± 0.05), with errors corresponding to 95% confidence intervals of the fit). Data are calculated for the different lattice site concentrations shown. Data points are the average of at least three experimental measurements with 95% confidence intervals shown.

Binding Model Derivation

Throughout the derivation, α denotes a specific ligand type under consideration, whereas i and j index general ligand types appearing in summations and neighbouring configurations. This derivation is largely based on the procedure outlined by Villaluenga *et al.*,[4] and by McGhee and von Hippel.[5]

Cooperativity parameter

The cooperativity parameter is defined as the ratio of the probability of ligands existing in an adjacent configuration (B), to the probability of ligands existing in a non-adjacent configuration (A) (Figure S19),

$$\begin{aligned}\omega_{ij} &= \frac{\text{probability of configuration II}}{\text{probability of configuration I}} \\ &= \frac{(\text{prob. config. A})\left(b^i_{m_i}b^j_1\right)\left(b^j_1b^j_2\right)\ldots\left(b^j_{m_j}f\right)(ff)^{x+y-1}(\text{prob. config. B})}{(\text{prob. config. A})\left(b^i_{m_i}f\right)(ff)^{x-1}\left(fb^j_1\right)\left(b^j_1b^j_2\right)\ldots\left(b^j_{m_j}f\right)(ff)^{y-1}(\text{prob. config. B})} \\ &= \frac{\left(b^i_{m_i}b^j_1\right)(ff)}{\left(b^i_{m_i}f\right)\left(fb^j_1\right)}.\end{aligned} \quad \text{Eq. S1}$$

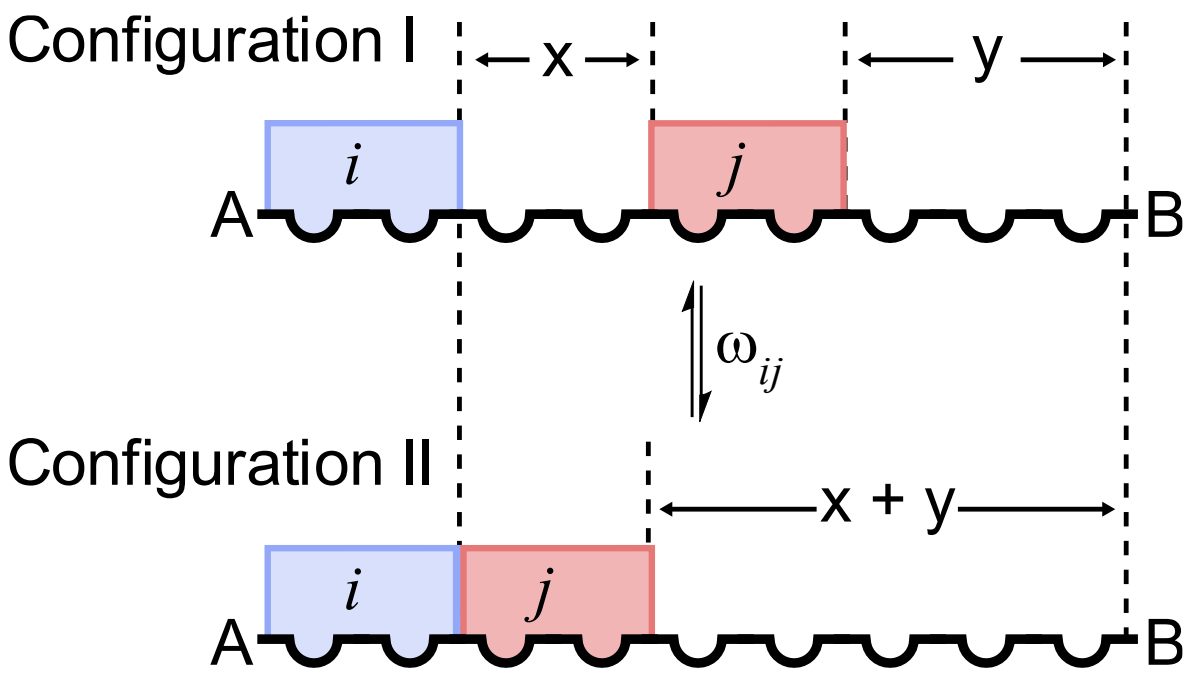

Figure S19. Definition of the cooperativity parameter ω used in this model.

Gap probability

A gap is defined as a sequence of g consecutive free lattice residues. A ligand i cannot bind to gaps smaller than their size ($g < m_i$), and g = 0 indicates that two ligands are adjacent. The probability that a given gap between a bound ligand of type i and a bound ligand of type j is g lattice residues long is given by

$$\left(P_g\right)_{ij} = \left(b^i_{m_i}f\right)(ff)^{g-1}\left(fb^j_1\right).$$

Computing the number of free binding sites

Derivation of the average number of free binding sites per lattice residue follows the procedure presented by Villaluenga *et al.*[4] The goal is to compute the average number of

free ligand binding sites per lattice residue, $\bar{s}_\alpha$. There are three types of free binding sites: an isolated site with no adjacent ligands ($\bar{s}_{iso}$), a singly contiguous site with one adjacent ligand ($\bar{s}_{sc}$), and a doubly contiguous site with two adjacent ligands ($\bar{s}_{dc}$). Following the notation used in Figure S19, if a ligand binds to an isolated site with affinity K_i, the affinity for a single contiguous site is $K_i\omega_{ij}$, and the affinity for a doubly contiguous site is $K_i\omega_{ij}^2$.

The number of binding sites in a gap of g lattice residues between two bound ligands is given by $g - m_\alpha + 1$ if $g \geq m_\alpha$, but zero if the gap is smaller than α. The probability of a gap being g free lattice residues long is denoted by (P_g), and is related to $\left(P_g\right)_{ij}$ by the sum

$$(P_g) = \sum_{i=1}^{T}\sum_{j=1}^{T}\left(P_g\right)_{ij}. \qquad \text{Eq. S2}$$

Consider a specific ligand α. For doubly contiguous sites, the gap size g must be exactly equal to the length of the ligand, m_α. The average number of doubly contiguous binding sites for ligand α in a gap flanked by ligand i on the left and ligand j on the right, $(\bar{s}_{dc})_{ij}^{\alpha}$, is given by

$$(\bar{s}_{dc})_{ij}^{\alpha} = \left(P_{m_i}\right)_{ij} = \left(b_{m_i}^{i} f\right)(ff)^{m_\alpha - 1}\left(f b_1^{j}\right). \qquad \text{Eq. S3}$$

By summing over different combinations of ligands i and j, weighted by the respective cooperativities, the average number of doubly contiguous binding sites for ligand α $(\bar{s}_{dc})^{\alpha}$ is obtained as

$$\begin{aligned}(\bar{s}_{dc})^{\alpha} &= \sum_{i=1}^{T}\sum_{j=1}^{T}\left(P_{m_\alpha}\right)_{ij}\omega_{\alpha i}\omega_{j\alpha},\\ &= \sum_{i=1}^{T}\sum_{j=1}^{T}\left(b_{m_i}^{i} f\right)(ff)^{m_\alpha - 1}\left(f b_1^{j}\right)\omega_{\alpha i}\omega_{j\alpha},\\ &= \left[\sum_{i=1}^{T}\frac{\theta_i}{\theta}\left(b_{m_i}^{i} f\right)\omega_{\alpha i}\right](ff)^{m_\alpha - 1}\left[\sum_{j=1}^{T}\left(f b_1^{j}\right)\omega_{j\alpha}\right]. \qquad \text{Eq. S4}\end{aligned}$$

For $g \geq m_\alpha + 1$, there are two singly contiguous binding sites. The average number of free singly contiguous binding sites for a specific ligand α flanked by ligand i on the left and ligand j on the right, $(\bar{s}_{sc})_{ij}^{\alpha}$, is given by the sum

$$(\bar{s}_{sc})_{ij}^{\alpha} = 2\sum_{g=m_\alpha+1}^{T}\left(P_g\right)_{ij} = 2\frac{\theta_i}{\theta}\left(b_{m_i}^{i} f\right)\frac{(ff)^{m_\alpha}}{1-(ff)}\left(f b_1^{j}\right). \qquad \text{Eq. S5}$$

By summing over different combinations of ligands i and j, the average number of singly contiguous binding sites $(\bar{s}_{sc})^{\alpha}$ is obtained as

$$(\bar{s}_{sc})^{\alpha} = \sum_{i=1}^{T}\sum_{j=1}^{T}(\bar{s}_{sc})_{ij}^{\alpha}\frac{\omega_{i\alpha}+\omega_{\alpha j}}{2}$$
$$= \sum_{i=1}^{T}\sum_{j=1}^{T}\frac{\theta_i}{\theta}(b_{m_i}^{i}f)\frac{(ff)^{m_\alpha}}{1-(ff)}(fb_1^{j})(\omega_{i\alpha}+\omega_{\alpha j}). \quad \text{Eq. S6}$$

Finally, for $g \geq m_\alpha + 2$, there are $(g - m_\alpha - 1)$ isolated binding sites. The sum then becomes

$$(\bar{s}_{iso})^{\alpha} = \sum_{i=1}^{T}\sum_{j=1}^{T}\sum_{g=m_\alpha+2}^{\infty}(g-m_\alpha-1)(P_g)_{ij}$$
$$= \left[\sum_{i=1}^{T}\frac{\theta_i}{\theta}(b_{m_i}^{i}f)\right]\frac{(ff)^{m_\alpha+1}}{\left(1-(ff)\right)^2}\left[\sum_{j=1}^{T}(fb_1^{j})\right]. \quad \text{Eq. S7}$$

Finally, the total average number of free binding sites is expressed as

$$(\bar{s})_{\alpha} = (\bar{s}_{dc})^{\alpha} + (\bar{s}_{sc})^{\alpha} + (\bar{s}_{iso})^{\alpha}$$
$$= \left[\sum_{i=1}^{T}\frac{\theta_i}{\theta}(b_{m_i}^{i}f)\left[\omega_{i\alpha}+\frac{(ff)}{1-(ff)}\right]\right](ff)^{m_\alpha+1}\left[\sum_{j=1}^{T}(fb_1^{j})\left[\omega_{\alpha j}+\frac{(ff)}{1-(ff)}\right]\right]. \quad \text{Eq. S8}$$

Multi-ligand, multi-site binding model equations

Equations can be derived to model the binding of an arbitrary number of ligands to an arbitrary number of types of binding site. Here, different "types of binding sites" refers to the different potential binding grooves on an amyloid fibril that share distinct populations of lattice residues available for binding. The derivation is provided in full, as an alternative to that provided in the manuscript text.

Consider a linear polymer composed of *N* individual lattice residues. A ligand of type i covers m_i lattice residues when bound and is described as consisting of m_i segments labelled 1, 2, ..., m_i from left to right. Across T total types of ligands there are therefore $1+\sum_{i=1}^{T} m_i$ possible types of residues in the linear polymer. Free residues are denoted f, and residues bound to a ligand are denoted by b_k^i for segment k of a ligand of type i.

Conditional probabilities are then defined as a sequence of two types of lattice residues. There are four relevant conditional probabilities: (ff) is the probability that, given a free residue, there is an adjacent free residue to the right; (fb_1^i) is the probability that, given a free residue, there is an adjacent residue bound to the first section of a ligand of type i to the right; $(b_{m_i}^i f)$ is the probability that, given a residue bound to the final section of a

ligand of type i, that there is an adjacent free residue to the right; $(b^i_{m_i} b^i_1)$ is the probability that, given a residue bound to the final section of a ligand of type i, that there is an adjacent residue bound to the first section of a ligand of type i to the right.

First, only two types of residues can lie to the immediate right of a free residue, yielding

$$(ff) + \sum_{i=1}^{T} (f b^i_1) = 1. \quad \text{Eq. S9}$$

If the final segment of a bound ligand is selected, only a free site or a second bound ligand, denoted as type j, can lie to the immediate right, giving

$$(b^i_{m_i} f) + \sum_{j=1}^{T} (b^i_{m_i} b^j_1) = 1, \quad \text{Eq. S10}$$

The coverage of a ligand is then defined as the fraction of lattice residues covered by a ligand of type i,

$$\theta_i = \frac{n_i m_i}{N}, \quad \text{Eq. S11}$$

where n_i ligands of type i are bound. If a residue is selected at random, the next residue will be free with a probability $(1-\theta)$ where $\theta = \sum_{i=1}^{T} \theta_i$, giving

$$(1-\theta)(ff) + \sum_{i=1}^{T} \frac{\theta_i}{m_i} (b^i_{m_i} f) = 1 - \theta. \quad \text{Eq. S12}$$

Cooperative binding affects these conditional probabilities and can be accounted for by introducing a cooperativity parameter, ω_{ij}, describing the equilibrium between binding configurations where two ligands are adjacent and non-adjacent.

While these conditional probabilities describe local adjacency, the number of available binding sites depends on stretches of free residues. Let α denote a specific ligand type to be considered. A gap is defined as a sequence of g free lattice residues, where $g = 0$ corresponds to adjacent ligands, and a ligand α can only bind if $g \geq m_\alpha$. Note that while conditional probabilities are defined per lattice residue, binding site availability is most naturally expressed per gap, following the statistical treatment introduced by McGhee and von Hippel.[24] This approach allows for the average number of free ligand binding sites per gap, $\bar{s}_\alpha$, to be computed (see SI for full derivation),

$$\bar{s}_\alpha = \left[\sum_{i=1}^{T} \frac{\theta_i}{\theta}\left(b_{m_i}^i f\right)\left[\omega_{i\alpha} + \frac{(ff)}{1-(ff)}\right]\right](ff)^{m_\alpha - 1}\left[\sum_{j=1}^{T}\left(f b_1^j\right)\left[\omega_{\alpha j} + \frac{(ff)}{1-(ff)}\right]\right]. \quad \text{Eq. S13}$$

The total number of free ligand binding sites can then be written as $\bar{s}_\alpha \cdot g$. As the total number of bound ligands is equal to the number of gaps plus one, the concentration of free binding sites $[s]$ is then $\bar{s}_\alpha \sum_{i=1}^{T}[L_i \cdot s]$ for $g \gg 1$, leading to the mass action equation,

$$\frac{[L_\alpha \cdot s]}{[L_\alpha]} = K_\alpha \bar{s}_\alpha \sum_{i=1}^{T}[L_i \cdot s]. \quad \text{Eq. S14}$$

These equations form the basis of a binding model that models each binding modes in Figure S25 and allows multiple ligands and binding sites to be considered, allowing for cooperativity and the effect of ligand size to be accounted for. The discrete binding mode will be used as a reference point in this work. Note that discrete binding is equivalent to a non-cooperative interacting binding mode (ω = 1), or an overlapping binding mode where ligand size is 1 (*m* = 1).

Final system of equations

The following final system of equations were used when generating synthetic data, or fitting experimental data, using the implemented mathematical model.

The first two equations used describe normalisation of the probability that only two types of sites can lie to the right of a free residue,

$$(ff) + \sum_{i=1}^{T}\left(f b_1^i\right) = 1, \quad \text{Eq. S15}$$

$$(1-\theta)(ff) + \sum_{i=1}^{T}\frac{\theta_i}{m_i}\left(b_{m_i}^i f\right) = 1 - \theta. \quad \text{Eq. S16}$$

Next, the probability of each type of lattice residue lying to the right of a ligand must sum to unity,

$$\left(b_{m_i}^i f\right) + \sum_{j=1}^{T}\left(b_{m_i}^i b_1^j\right) = 1. \quad \text{Eq. S17}$$

Given the cooperativity parameter defines the probability of having two ligands adjacent,

$$\omega_{ij} = \frac{\left(b_{m_i}^{i} b_1^{j}\right)(ff)}{\left(b_{m_i}^{i} f\right)\left(f b_1^{j}\right)}, \qquad \text{Eq. S18}$$

then, for a specific ligand α

$$\left(b_{m_\alpha}^{\alpha} f\right) + \sum_{j=1}^{T} \omega_{\alpha j} \frac{\left(b_{m_\alpha}^{\alpha} f\right)\left(f b_1^{j}\right)}{\left(b_{m_\alpha}^{\alpha} b_1^{j}\right)} = 1. \qquad \text{Eq. S19}$$

The mass action equation can then be implemented

$$\frac{[L_\alpha \cdot s]}{[L_\alpha]} = K_\alpha \bar{s}_\alpha \sum_{i=1}^{T} [L_i \cdot s]. \qquad \text{Eq. S20}$$

Note that for a system with only a single ligand, this reduces to

$$1 = K_\alpha \bar{s}_\alpha [L_\alpha] \qquad \text{Eq. S21}$$

Finally, the conditional probabilities and the concentration of each species can be linked. A bound ligand α either follows a free residue, with conditional probability $(f b_1^{\alpha})$, or the final residue of another ligand, with conditional probability $\left(b_{m_i}^{i} b_1^{\alpha}\right)$. Weighting these probabilities with the concentration of free lattice residue and the summed concentrations of bound ligands of type i must yield the total concentration of a bound ligand of type α, expressed as

$$\begin{aligned} [L_\alpha \cdot s] &= \left[r_{free}\right](f b_1^{\alpha}) + \sum_{i=1}^{T} [L_i \cdot s]\left(b_{m_i}^{i} b_1^{\alpha}\right) \\ &= \left[r_{free}\right](f b_1^{\alpha}) + \sum_{i=1}^{T} [L_i \cdot s]\,\omega_{i\alpha} \frac{\left(b_{m_i}^{i} f\right)(f b_1^{\alpha})}{(ff)}, \end{aligned} \qquad \text{Eq. S22}$$

where $\left[r_{free}\right]$ is the concentration of free lattice residues.

Finally, conservation is enforced for the total concentration of each ligand by:

$$[L_\alpha] + [L_\alpha \cdot s] = [L_\alpha]_{tot} \qquad \text{Eq. S23}$$

Where $[L_\alpha]_{tot}$ is the total concentration of ligand α present.

Equations S8, S9, S10, S11, S13, S14, and S15 are implemented in the provided code. Note that this approach can be extended to multiple types of binding sites by introducing sums over different site types.

Synthetic Binding Data

Additional one-site synthetic binding data

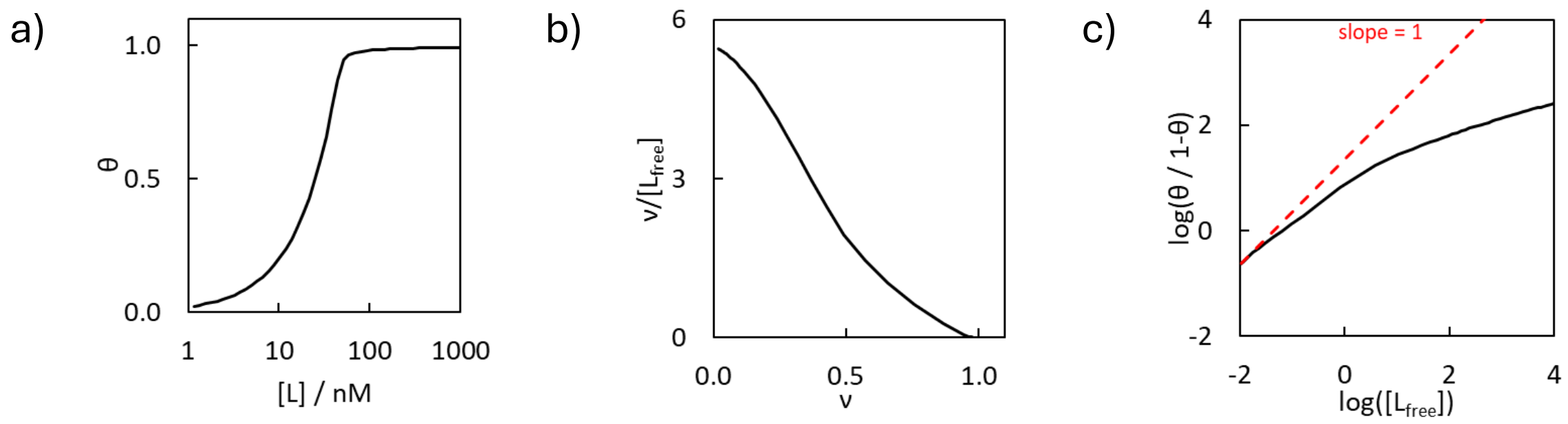

Figure S20. Representative (a) binding isotherm, (b) Scatchard plot, and (c) Hill plot for one ligand binding to two overlapping-interacting sites (K_1 = 1, K_2 = 10, m_1 = m_2 = 4, ω_1 = ω_2 = 4).

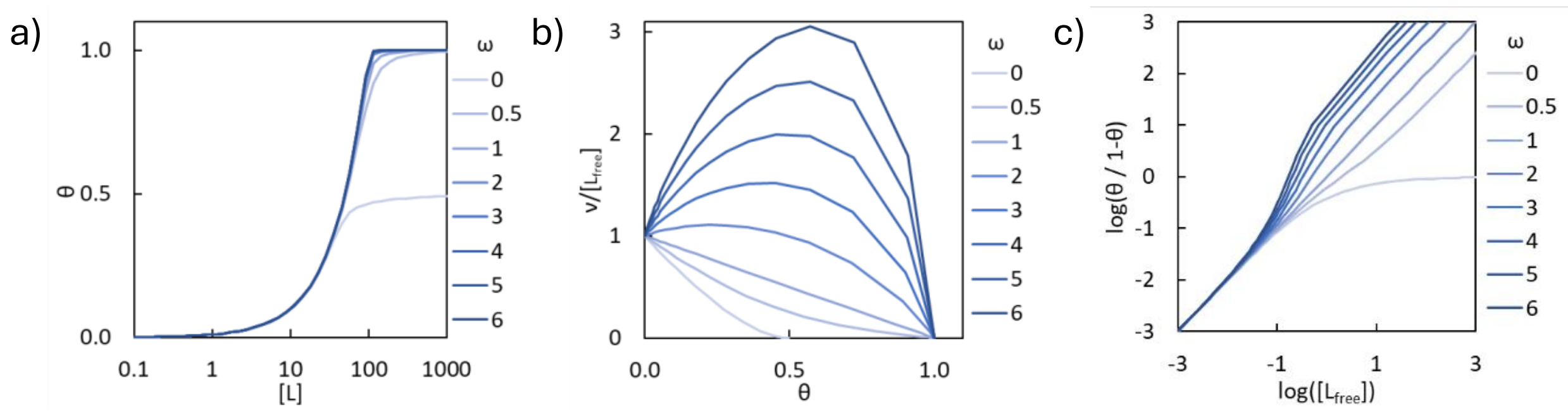

Figure S21. Representative (a) binding isotherm, (b) Scatchard plot, and (c) Hill plot for a ligand (m = 1) binding to a single site with different cooperativity parameter values.

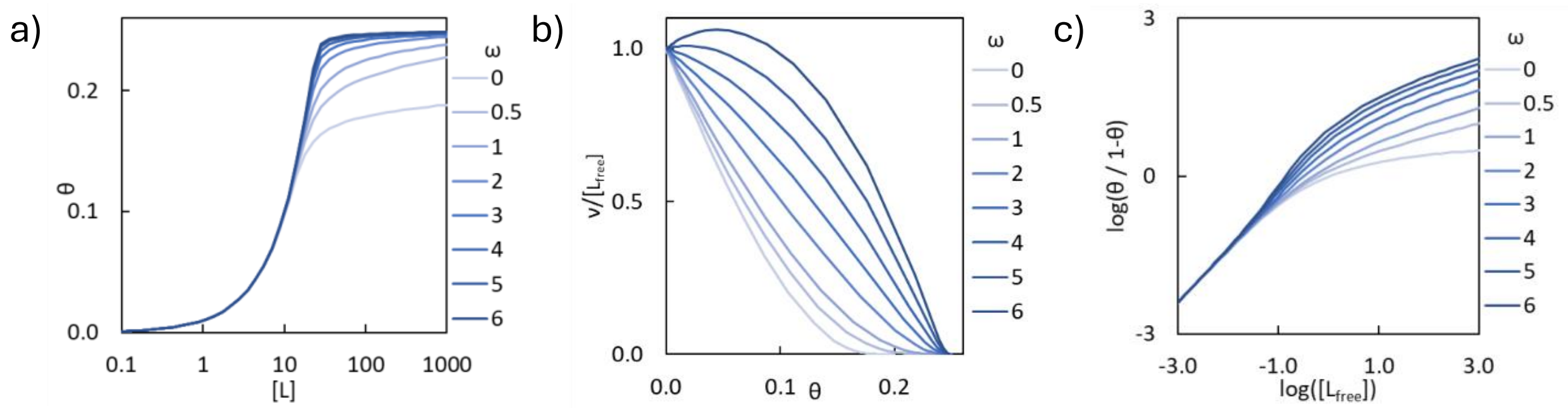

Figure S22. Representative (a) binding isotherm, (b) Scatchard plot, and (c) Hill plot for a ligand (m = 4) binding to a single site with different cooperativity parameter values.

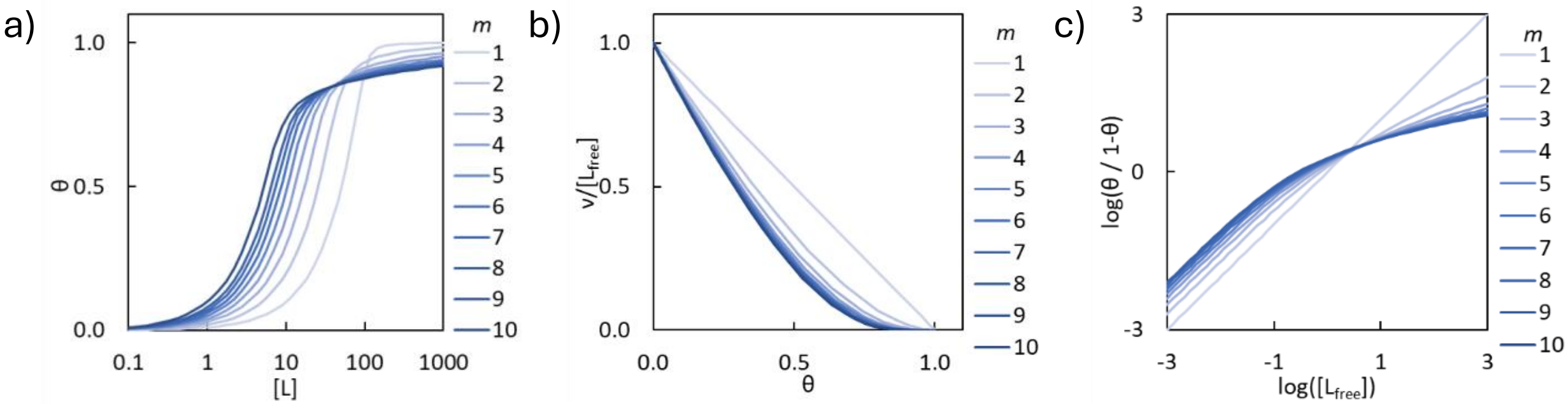

Figure S23. Representative (a) binding isotherm, (b) Scatchard plot, and (c) Hill plot for a non-cooperative ligand (ω = 1) binding to a single site with different ligand size values.

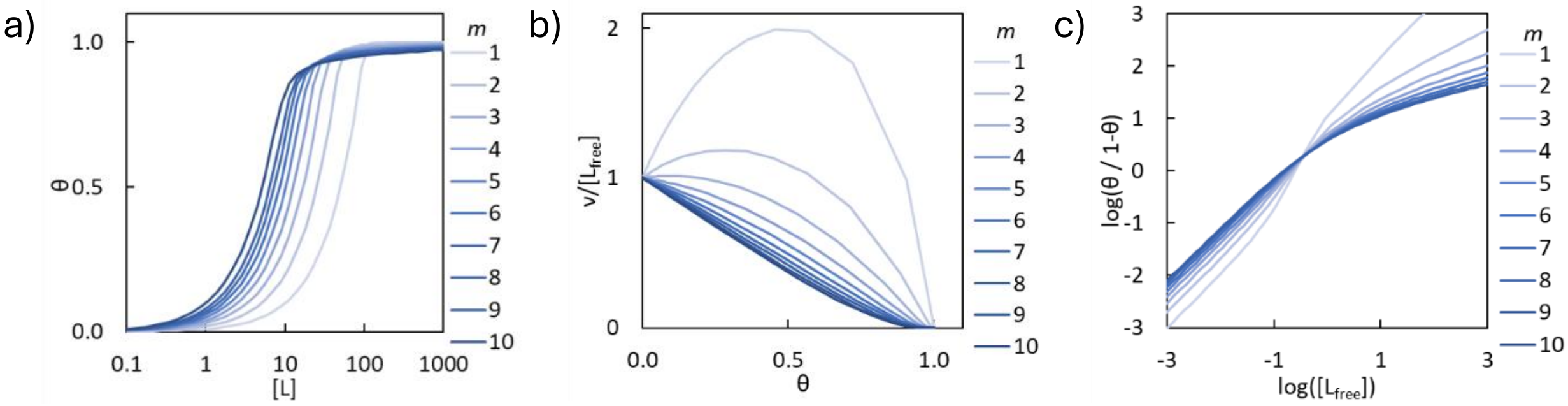

Figure S24. Representative (a) binding isotherm, (b) Scatchard plot, and (c) Hill plot for a cooperatively-binding ligand (ω = 4) binding to a single site with different ligand size values.

Overlapping new-site synthetic binding data

An *overlapping-new site* binding mode, where binding of one ligand creates a new site underpinned by ligand-ligand interactions, has also been suggested (Figure S25).[6] These structures were obtained using concentrations of ligand (50-500 μM) at least an order of magnitude greater than their dissociation constants. An overlapping-new site binding mode was modelled by introducing a second type of binding site for every ligand bound directly to the polymer. An overlapping-new site model demonstrated similar plotted features to an overlapping model except the Hill coefficient had a non-linear relationship with lattice site occupancy, reflecting the creation of additional binding sites upon ligand binding (Figure S26). Overlapping and overlapping-new site models are therefore unlikely to be distinguishable from saturation data alone.

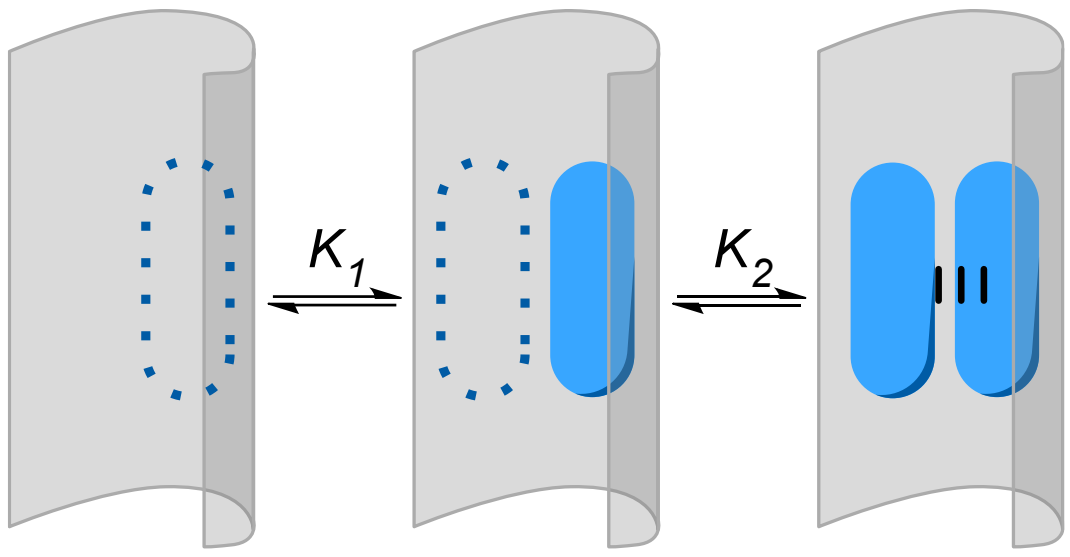

Figure S25. Overlapping-new site binding mode, where a bound ligand generates a new binding site. K is the binding constant, and ω is the cooperativity parameter.

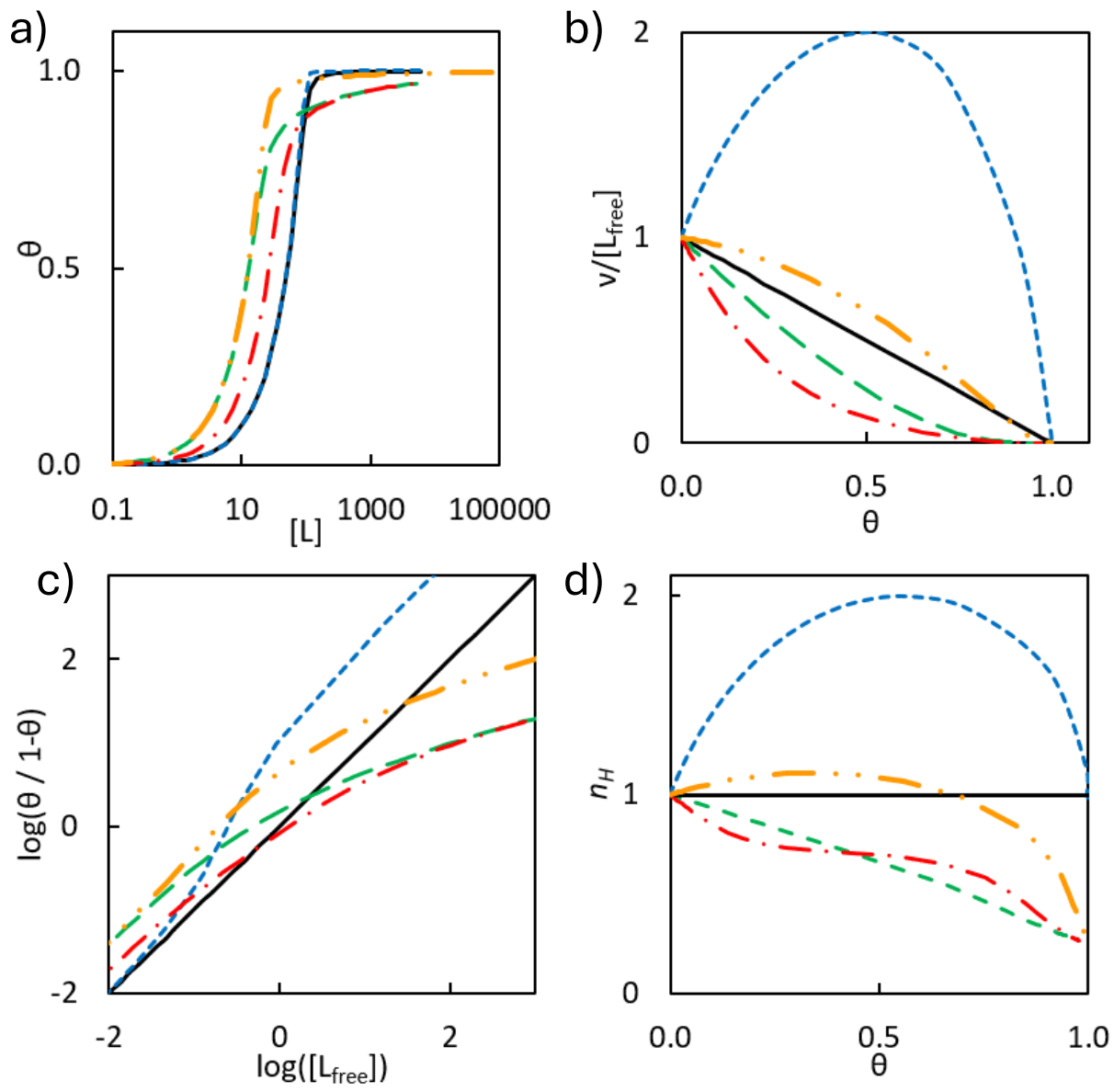

Figure S26. Representative (a) binding isotherms, (b) Scatchard plots, (c) Hill plots, and (d) local Hill coefficient plot for discrete binding (black line), interacting binding (m = 1, ω = 4, blue short-dash line), overlapping binding (m = 4, ω = 1, green long-dash line), overlapping-new site binding (m = 4, ω = 1, red dot-dash line), and interacting-overlapping binding (m = 4, ω = 1, orange dot-dot-dash line), calculated with K = 1 and a lattice site concentration of 100. ν is the average number of ligands bound per unit lattice.

Two-site synthetic binding data

As multiple binding grooves appear to exist on amyloid fibrils, isotherms were computed for a single type of ligand binding to two different types of binding sites. In the absence of any cooperativity and *m* = 1, a single ligand binding to two sites with different affinities yielded a concave-up Scatchard plot resembling overlapping binding (Figure S27a). The slope of the Hill plot had a local minima below one and returned to unity at low and high occupancy, whereas the Hill coefficient for single-site overlapping binding was monotonically decreasing with increasing lattice site occupancy (Figure S27b).

In other two-site models, a single cooperative site introduced concave-down character in Scatchard plots and a region in the Hill coefficient plot where n_H exceeds unity (Figure S27a-b). A single overlapping site produced concave-up character in Scatchard plots and a Hill coefficient less than unity at high lattice site occupancies (Figure S27a-b). Two cooperative sites produced a concave-down Scatchard plot with a shoulder and a Hill coefficient plot with two local maxima (Figure S27c-d). Two overlapping sites produced similar plots to the overlapping-new site model (Figure S27c-d). If one overlapping site and one cooperative site was present, the resultant Scatchard and Hill plots combined these features (Figure S27a-b). Characteristic features of Scatchard and Hill coefficient plots are therefore useful in identifying the underlying binding mode present (Table S1). More complex multi-site models will introduce additional features that would likely be challenging to distinguish.

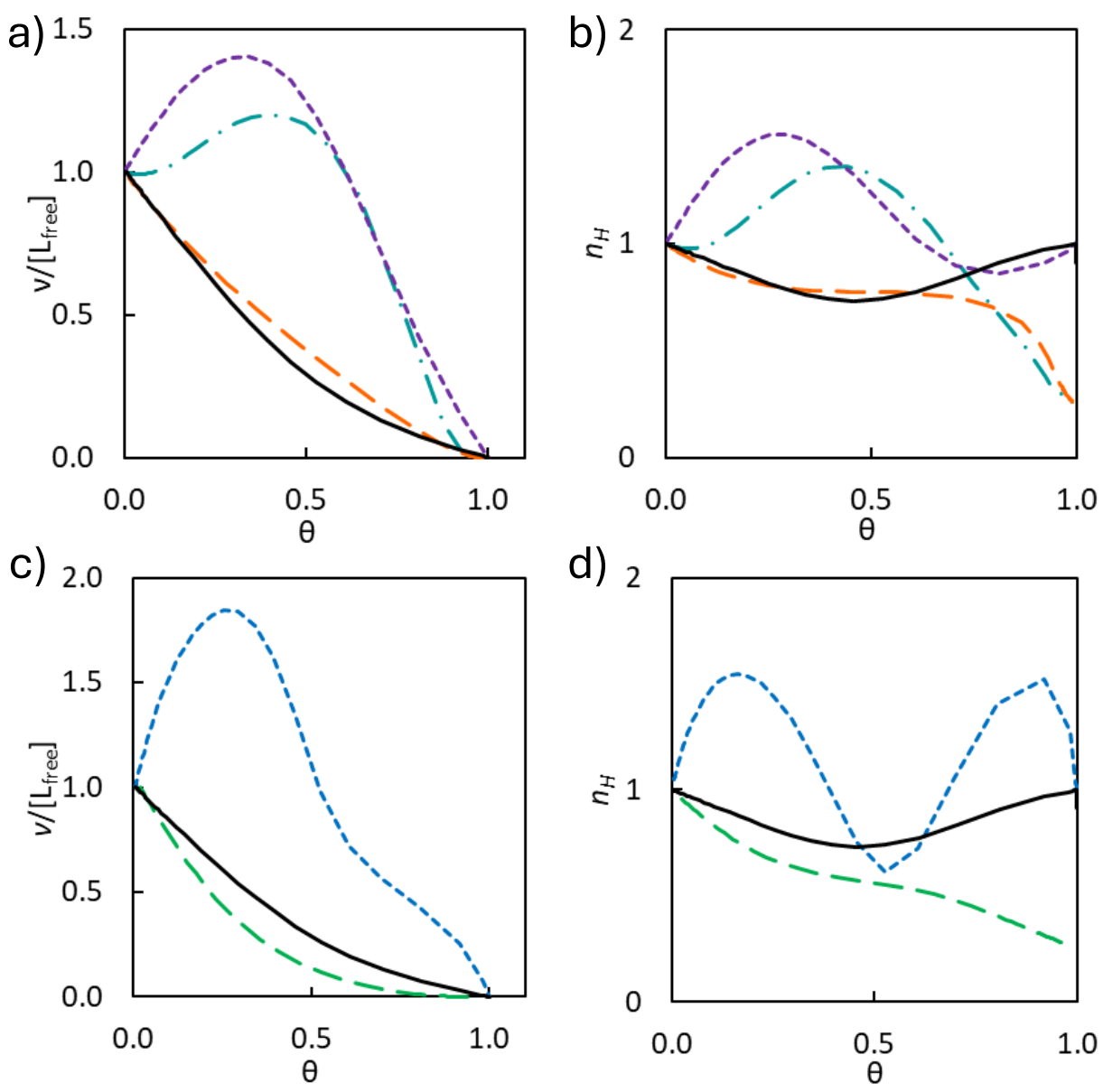

Figure S27. Representative plots for one-ligand, two-site binding models. (a) Scatchard plot and (b) Hill plot for the binding of a ligand to one interacting and one overlapping site (teal dash-dot line), one discrete and one interacting site (purple short-dash line), and one discrete and one overlapping site (orange long-dash line) with the same affinity (K_1 = K_2 = 1). (c) Scatchard plot and (d) Hill plot for the binding of a ligand to two discrete sites (black line), two interacting sites (blue short-dash line), and two overlapping sites (green

long-dash line) with different binding affinities ($K_1 = 1$, $K_2 = 10$). Data for two discrete sites (black line, $K_1 = 1$, $K_2 = 10$) are plotted as a reference. Overlapping binding was modelled with m = 4, and interacting binding was modelled with ω = 4.

Table S1. Expanded table of diagnostic features for different binding modes.

Scatchard Features	Local Hill Coefficient Features	Possible Binding Sites
Concave down	One maxima	1 interacting
	Two maxima	2 interacting
	Ona maxima, one minima	1 discrete + 1 interacting
Concave up	Linearly decreasing	1 overlapping
	Inflection point	1 overlapping new-site, or 2 overlapping, or 1 discrete + 1 overlapping
	One minima	2 discrete
Concave up and concave down sections	Concave up and concave down sections	1 interacting + 1 overlapping
Concave down or up (depending on magnitude of *m* and *ω*)	Concave down, $n_H = 1$ at $\theta = 0$ and $n_H = 0$ at $\theta = 1$	1 interacting-overlapping

Synthetic binding data generation

Unless otherwise specified, all synthetic binding data presented in the manuscript were generated using "*Model - Main Fitter.ipynb*", with a ligand size (*m*) of 1, a cooperativity parameter (ω) of 1, an association constant (K_a) of 1, and a receptor concentration of 100.

The following parameters were used in site_params to generate data presented in different figures:

For Figure 3:

- One discrete 1:1 binding site (equivalent to non-cooperative interacting binding):

```
site_params = [
    dict(n_A = 1, n_B = 1, res_tot=100, K_A=1, K_B=0, omega_AA=1, omega_AB=1, omega_BA=1, omega_BB=1)
]
```

- One interacting binding site:

```
site_params = [
    dict(n_A = 1, n_B = 1, res_tot=100, K_A=1, K_B=0, omega_AA=4, omega_AB=1, omega_BA=1, omega_BB=1)
]
```

- One overlapping binding site:

```
site_params = [
    dict(n_A = 4, n_B = 1, res_tot=100, K_A=1, K_B=0, omega_AA=1, omega_AB=1, omega_BA=1, omega_BB=1)
]
```

- One overlapping new-site binding site (called with new_site=True):

```
site_params = [
    dict(n_A = 4, n_B = 1, res_tot=100, K_A=10, omega_AA=1, K2=1)
]
```

For Figure S18:

- Two discrete 1:1 binding sites (equivalent to non-cooperative interacting binding):

```
site_params = [
    dict(n_A = 1, n_B = 1, res_tot=100, K_A=1, K_B=0,
omega_AA=1, omega_AB=1, omega_BA=1, omega_BB=1),
    dict(n_A = 1, n_B = 1, res_tot=100, K_A=10, K_B=0,
omega_AA=1, omega_AB=1, omega_BA=1, omega_BB=1)
]
```

- One interacting and one overlapping binding site:

```
site_params = [
    dict(n_A = 1, n_B = 1, res_tot=100, K_A=1, K_B=0,
omega_AA=4, omega_AB=1, omega_BA=1, omega_BB=1),
    dict(n_A = 4, n_B = 1, res_tot=100, K_A=1, K_B=0,
omega_AA=1, omega_AB=1, omega_BA=1, omega_BB=1)
]
```

- One discrete and one interacting binding site:

```
site_params = [
    dict(n_A = 1, n_B = 1, res_tot=100, K_A=1, K_B=0,
omega_AA=1, omega_AB=1, omega_BA=1, omega_BB=1),
    dict(n_A = 1, n_B = 1, res_tot=100, K_A=1, K_B=0,
omega_AA=4, omega_AB=1, omega_BA=1, omega_BB=1)
]
```

- One discrete and one overlapping binding site:

```
site_params = [
    dict(n_A = 1, n_B = 1, res_tot=100, K_A=1, K_B=0,
omega_AA=1, omega_AB=1, omega_BA=1, omega_BB=1),
    dict(n_A = 4, n_B = 1, res_tot=100, K_A=1, K_B=0,
omega_AA=1, omega_AB=1, omega_BA=1, omega_BB=1)
]
```

- Two interacting binding sites:

```
site_params = [
    dict(n_A = 1, n_B = 1, res_tot=100, K_A=1, K_B=0,
omega_AA=4, omega_AB=1, omega_BA=1, omega_BB=1),
    dict(n_A = 1, n_B = 1, res_tot=100, K_A=1, K_B=0,
omega_AA=4, omega_AB=1, omega_BA=1, omega_BB=1)
]
```

- Two overlapping binding sites:

```
site_params = [
    dict(n_A = 4, n_B = 1, res_tot=100, K_A=1, K_B=0,
omega_AA=1, omega_AB=1, omega_BA=1, omega_BB=1),
    dict(n_A = 4, n_B = 1, res_tot=100, K_A=1, K_B=0,
omega_AA=1, omega_AB=1, omega_BA=1, omega_BB=1)
]
```

Code for Isotherm Generation and Fitting.

An attached iPython notebook, "*Model - Main Fitter.ipynb*" is provided with code required to generate synthetic binding isotherms for the following models:

- One ligand binding to one or more types of sites
- Two ligands binding to one or more types of sites
- One ligand binding to one or more types of sites. Upon binding to a site, a secondary site is generated

An attached python file, "*speciation_1Dbinding.py*", is provided as a substitution for the "*speciation.py*" file incorporated in *Musketeer*. This code contains three new speciation classes to fit:

- Ligands binding to one type of site (SpeciationPolymer1D_1s)
- Ligands binding to two types of sites (SpeciationPolymer1D_2s)
- Ligands binding to one type of site, allowing for adjacent and non-adjacent ligands to have different fluorescence spectra (SpeciationPolymer1D_1s_adjacency)

A specific input format is required when entering concentrations in the Musketeer speciation table: enter columns of host concentrations first, followed by columns of guest concentrations.

When fitting data, an initial guess for binding affinity of 1,000,000 and an initial guess for host concentration of 1 μM was used. Free ligand spectra were input based on control titrations in the absence of any fibril, and bound ligand spectra were fitted. Fibril spectra was fixed to zero at all wavelengths (negligibly fluorescent).

Code was written with assistance from ChatGPT 5.2.

Comparing Fits

When fitting raw data, models were compared using either RMSE values generated by *Musketeer*,[8] or the Akaike Information Criterion corrected for small sample sizes (AICc). When using AICc the effective sample size was taken as the number of additions in the binding assay, each addition corresponding to a fluorescence emission spectrum obtained at a specific ligand concentration. The data fitting procedure generates a fitted optical brightness for the bound species at each wavelength; comparable fits were obtained when fitting only a single wavelength, and so each brightness spectrum was treated as a single effective parameter.

Comparing fitted dissociation constants for different values of *m*

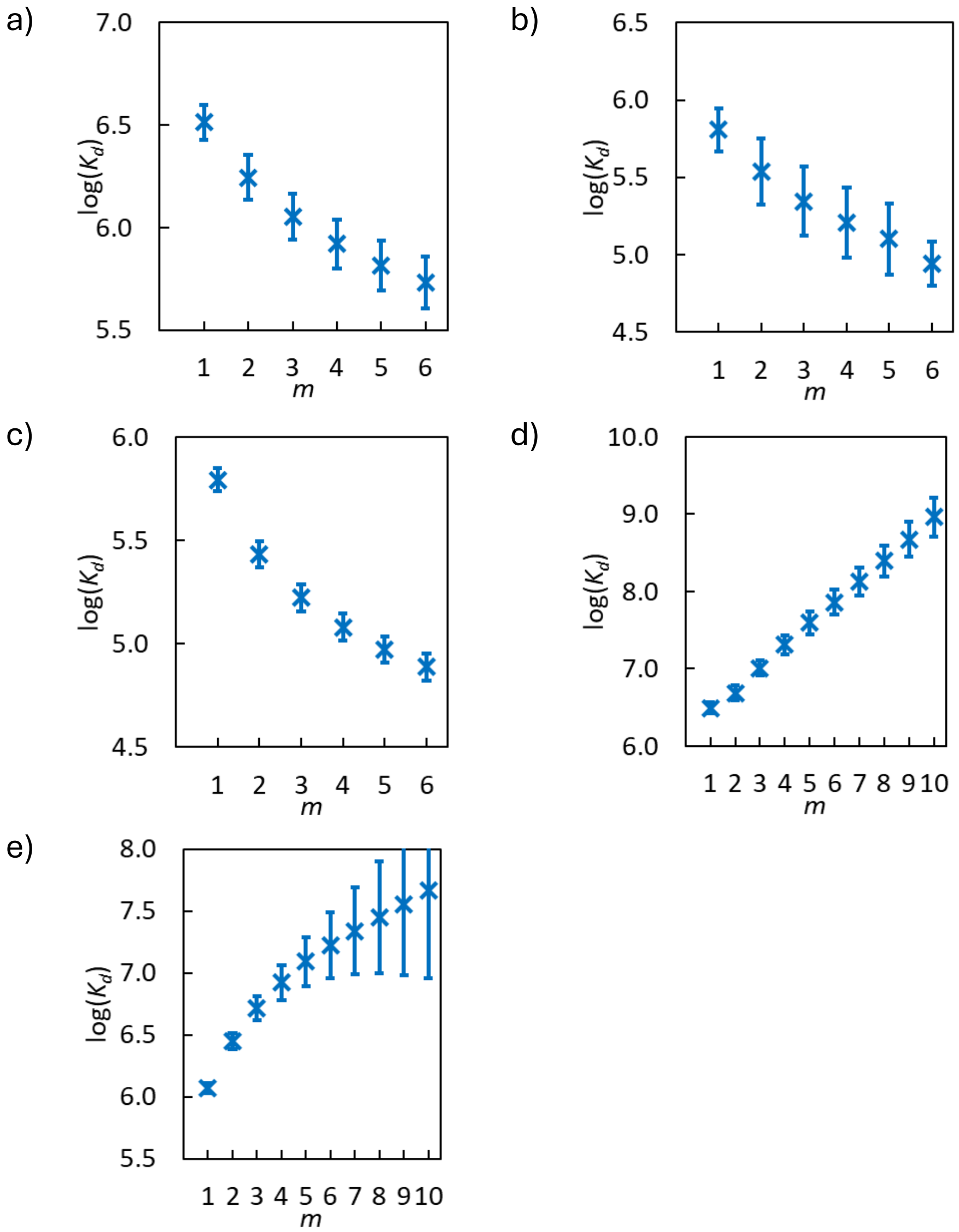

Figure S28. Plots of log(K_d) fitted to an overlapping binding model (ω = 1) with varying ligand size *m*. Data is shown for (a) **ThT** binding to PD fibrils; (b) **ThT** binding to αSyn-PBS fibrils; (c) **ThT** binding to αSyn-Tris fibrils; (d) **1** binding to αSyn-PBS fibrils; (e) **1** binding to αSyn-Tris fibrils. Data points are the average of at least three experimental measurements with 95% confidence intervals shown.

Symbols Used in Equations

$[r_{free}]$	The concentration of free lattice residues
$[s]_t$	Total concentration of binding sites
$[s]$	Concentration of free binding sites
$[L_\alpha]$	Free concentration of a ligand of type α
$[L_\alpha]_{tot}$	Total concentration of a ligand of type α
$[L_\alpha \cdot s]$	Concentration of a ligand of type α bound to site s
f	A free lattice residue
$b^i_{m_i}$	A lattice residue bound to the m_ith segment of a ligand of type i
K_α	The equilibrium binding constant for ligand α
ω_{ij}	The cooperativity parameter between a ligand of type i and a ligand of type j
n_H	The Hill coefficient
m_i	Size of ligand of type i in lattice units
ν_i	The average number of ligands of type i bound per unit lattice
θ_i	Coverage for ligand of type i
θ	Total coverage of the lattice
n_i	The number of ligands of type i bound
N	Total number of lattice units
T	Total number of types of ligands
g	The size of a gap in lattice units
$(P_g)_{ij}$	The probability that a gap between a ligand of type i and a ligand of type j is g lattice residues long
(P_g)	The probability that a gap being g lattice residues long
$\bar{s}_\alpha$	The average number of free binding sites per gap for ligand α
$(\bar{s}_{iso})^\alpha_{ij}$	The average number of isolated binding sites for ligand α in a gap flanked by a ligand of type i on the left and a ligand of type j on the right
$(\bar{s}_{sc})^\alpha_{ij}$	The average number of singly contiguous sites for ligand α in a gap flanked by a ligand of type i on the left and a ligand of type j on the right
$(\bar{s}_{dc})^\alpha_{ij}$	The average number of doubly contiguous binding sites for ligand α in a gap flanked by a ligand of type i on the left and a ligand of type j on the right
$(\bar{s}_{iso})^\alpha$	The average number of isolated binding sites for ligand α in a gap
$(\bar{s}_{sc})^\alpha$	The average number of singly contiguous binding sites for ligand α in a gap
$(\bar{s}_{dc})^\alpha$	The average number of doubly contiguous binding sites for ligand α in a gap

References

(1) Chisholm, T. S.; Hunter, C. A. Ligand Profiling to Characterize Different Polymorphic Forms of α-Synuclein Aggregates. *J. Am. Chem. Soc.* **2023**, *145* (49), 27030–27037. https://doi.org/10.1021/jacs.3c10521.

(2) Buell, A. K.; Galvagnion, C.; Gaspar, R.; Sparr, E.; Vendruscolo, M.; Knowles, T. P. J.; Linse, S.; Dobson, C. M. Solution Conditions Determine the Relative Importance of Nucleation and Growth Processes in α-Synuclein Aggregation. *Proc. Natl. Acad. Sci. U. S. A.* **2014**, *111* (21), 7671–7676. https://doi.org/10.1073/pnas.1315346111.

(3) Chisholm, T. S. The Morphology of α-Synuclein Fibrils Changes during Formation, Storage, and upon Exposure to Ligands. bioRxiv August 30, 2025, p 2025.08.29.672948. https://doi.org/10.1101/2025.08.29.672948.

(4) Villaluenga, J. P. G.; Brunete, D.; Cao-García, F. J. Competitive Ligand Binding Kinetics to Linear Polymers. *Phys. Rev. E* **2023**, *107* (2), 024401. https://doi.org/10.1103/PhysRevE.107.024401.

(5) McGhee, J. D.; von Hippel, P. H. Theoretical Aspects of DNA-Protein Interactions: Co-Operative and Non-Co-Operative Binding of Large Ligands to a One-Dimensional Homogeneous Lattice. *J. Mol. Biol.* **1974**, *86* (2), 469–489. https://doi.org/10.1016/0022-2836(74)90031-X.

(6) Shi, Y.; Murzin, A. G.; Falcon, B.; Epstein, A.; Machin, J.; Tempest, P.; Newell, K. L.; Vidal, R.; Garringer, H. J.; Sahara, N.; Higuchi, M.; Ghetti, B.; Jang, M. K.; Scheres, S. H. W.; Goedert, M. Cryo-EM Structures of Tau Filaments from Alzheimer's Disease with PET Ligand APN-1607. *Acta Neuropathol. (Berl.)* **2021**, *1*, 3. https://doi.org/10.1007/s00401-021-02294-3.

(7) Chisholm, T. S.; Hunter, C. A. Ligands for Protein Fibrils of Amyloid-β, α-Synuclein, and Tau. *Chem. Rev.* **2025**, *125* (11), 5282–5348. https://doi.org/10.1021/acs.chemrev.4c00838.

(8) O. Soloviev, D.; A. Hunter, C. Musketeer: A Software Tool for the Analysis of Titration Data. *Chem. Sci.* **2024**, *15* (37), 15299–15310. https://doi.org/10.1039/D4SC03354J.

(9) Liu, K.; Tao, Y.; Zhao, Q.; Xia, W.; Li, X.; Zhang, S.; Yao, Y.; Xiang, H.; Han, C.; Tan, L.; Sun, B.; Li, D.; Li, A.; Liu, C. Binding Adaptability of Chemical Ligands to Polymorphic α-Synuclein Amyloid Fibrils. *Proc. Natl. Acad. Sci.* **2024**, *121* (35), e2321633121. https://doi.org/10.1073/pnas.2321633121.

(10) RDKit: Open-Source Cheminformatics. http://www.rdkit.org.
